# A severe leakage of intermediates to shunt products in acarbose biosynthesis

**DOI:** 10.1038/s41467-020-15234-8

**Published:** 2020-03-19

**Authors:** Qinqin Zhao, Yuchang Luo, Xin Zhang, Qianjin Kang, Dan Zhang, Lili Zhang, Linquan Bai, Zixin Deng

**Affiliations:** 1 State Key Laboratory of Microbial Metabolism, School of Life Sciences and Biotechnology, Shanghai Jiao Tong University, 200240 Shanghai, China; 2 Joint International Research Laboratory of Metabolic and Developmental Sciences, Shanghai Jiao Tong University, 200240 Shanghai, China; 3 College of Life Science, Tarim University, Alar, 843300 Xinjiang China

**Keywords:** Genetic engineering, Metabolic engineering, Metabolic pathways

## Abstract

The α-glucosidase inhibitor acarbose, produced by *Actinoplanes* sp. SE50/110, is a well-known drug for the treatment of type 2 diabetes mellitus. However, the largely unexplored biosynthetic mechanism of this compound has impeded further titer improvement. Herein, we uncover that 1-*epi*-valienol and valienol, accumulated in the fermentation broth at a strikingly high molar ratio to acarbose, are shunt products that are not directly involved in acarbose biosynthesis. Additionally, we find that inefficient biosynthesis of the amino-deoxyhexose moiety plays a role in the formation of these shunt products. Therefore, strategies to minimize the flux to the shunt products and to maximize the supply of the amino-deoxyhexose moiety are implemented, which increase the acarbose titer by 1.2-fold to 7.4 g L^−1^. This work provides insights into the biosynthesis of the C_7_-cyclitol moiety and highlights the importance of assessing shunt product accumulation when seeking to improve the titer of microbial pharmaceutical products.

## Abstract (web summary)

Biosynthetic mechanism for the type 2 diabetes treatment drug acarbose is not fully revealed. Here, the authors show that shunt pathways and inefficient amino-deoxyhexose biosynthesis lead to 1-*epi*-valienol and valienol accumulation, and minimizing the flux to these shunt products can increase acarbose titer in *Actinoplanes* species.

## Introduction

The α-glucosidase inhibitor acarbose (**1**) is one of the prominent representatives of C_7_N-aminocyclitol-containing natural products and has been used for the clinical treatment of type 2 diabetes mellitus since it was introduced to the market in 1990^1,2^. This pseudosugar-containing oligosaccharide has competitive inhibitory activities toward intestinal maltase, sucrase, dextrinase, and glucoamylase, and thereby delays carbohydrate absorption and controls postprandial hyperglycemia^1,3^. It is produced in large-scale fermentation by *Actinoplanes* sp. obtained from rounds of mutagenesis and selection^1,4,5^. However, since type 2 diabetes mellitus becomes more prevalent worldwide, the market demand for **1** increases rapidly^6^, which encourages us to develop high-performance producers with improved productivity.

In recent years, comparative genome, transcriptome, and proteome analyses have provided systems-level understanding of *Actinoplanes* sp. SE50/110 and insights into the mechanisms of **1** overproduction^7–10^. In addition, highly efficient genetic manipulation systems have been established for *Actinoplanes* sp. SE50/110 and are successfully used to delete *melC2* (a tyrosinase gene) and *treY* (a maltooligosyltrehalose synthase gene), which eliminate the formation of eumelanin and the by-product component C, respectively^11–13^. These advances in the omics analysis and genetic toolbox development have paved the way to genetically engineer *Actinoplanes* sp. SE50/110 to become a more efficient biofactory of **1**^14–16^.

**Fig. 1:**
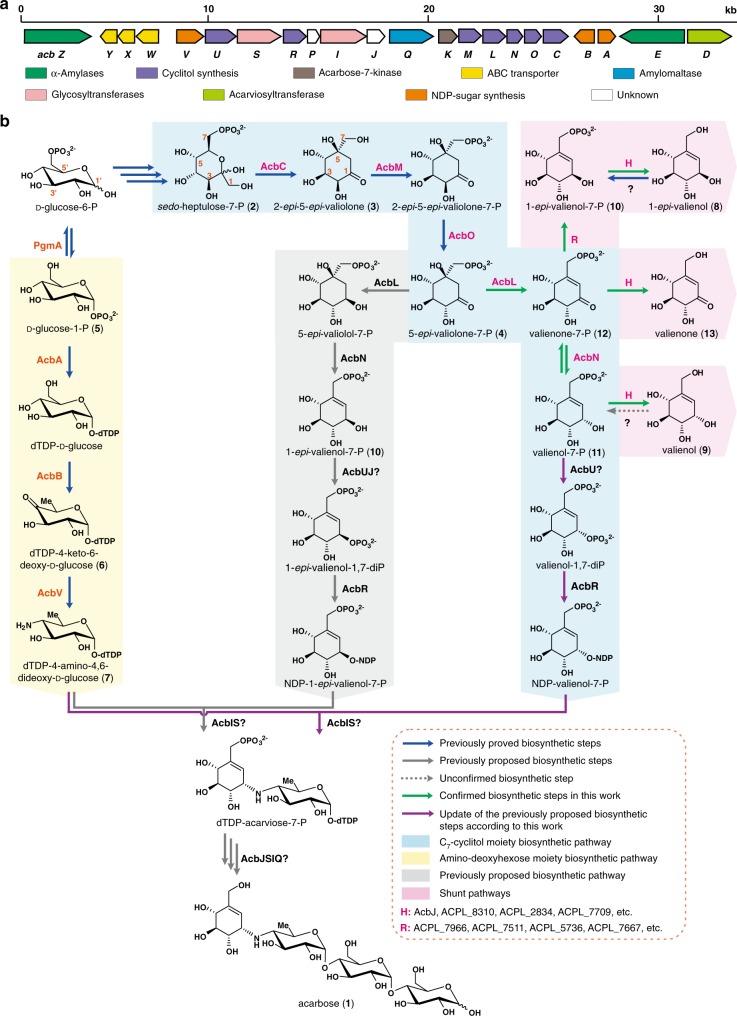
The biosynthesis of acarbose in *Actinoplanes* species. **a** The biosynthetic gene cluster of acarbose (**1**) (*acb* cluster, GenBank accession no. Y18523.4). **b** The biosynthetic pathway to **1**, including previously determined biosynthetic steps (blue arrow), possible conversion without experimental confirmation (gray dashed arrow), previously proposed biosynthetic steps (gray arrow), confirmed biosynthetic steps in this work (green arrow), and update of the previously proposed biosynthetic steps according to this work (purple arrow). The biosynthetic pathways to the C_7_-cyclitol moiety, the amino-deoxyhexose moiety, and the shunt products are highlighted in blue, yellow, and plum, respectively, and the previously proposed biosynthetic pathway to the C_7_-cyclitol moiety is highlighted in gray.

However, attempts to substantially improve the titer of **1** also require a clear understanding of the biosynthetic pathway to **1**, as this information will enable identification of potential targets for gene manipulation and biosynthetic flux modulation. The biosynthetic gene cluster of **1** (*acb* cluster) in *Actinoplanes* sp. comprises 22 open reading frames responsible for the biosynthesis and export of **1** and sugar metabolism^1,17,18^ (Fig. 1a; Supplementary Table 1). The structure of **1** consists of three moieties: an unsaturated C_7_-cyclitol, an amino-deoxyhexose, and a maltose. The biosynthesis of the C_7_-cyclitol moiety has been demonstrated to be initiated by the cyclization of *sedo*-heptulose-7-phosphate (*sedo*-heptulose-7-P, **2**) by AcbC to give 2-*epi*-5-*epi*-valiolone (**3**), which is subsequently phosphorylated by AcbM and isomerized by AcbO to give intermediate 5-*epi*-valiolone-7-P (**4**)^19–21^. On the other hand, the amino-deoxyhexose moiety has been shown to derive from glucose-1-P (**5**) catalyzed by D-glucose-1-P thymidylyltransferase (AcbA, abbreviated as G-1-PT) and dTDP-D-glucose 4,6-dehydratase (AcbB, 4,6-DH) to give dTDP-4-keto-6-deoxy-D-glucose (**6**), followed by transamination catalyzed by AcbV to give dTDP-4-amino-4,6-dideoxy-D-glucose (**7**)^1,22^. Further downstream, the pathway to **1** has been proposed to involve modifications of **4**, condensation of the C_7_-cyclitol moiety with **7**, and the attachment of maltose to form **1**, without experimental evidence (Fig. 1b)^1,21,23,24^. The limited knowledge of the biosynthetic pathway to **1** has made it difficult to identify critical biosynthetic bottlenecks and to prioritize engineering strategies for the overproduction of **1**.

In the present study, we identified two shunt products that are derived from the biosynthetic pathway of **1** in the fermentation broth of *Actinoplanes* sp. SE50/110. Upon systematic investigation of these products and their modes of formation, we further clarify the biosynthetic pathway to the C_7_-cyclitol moiety in **1**. Subsequently, we employ multiple metabolic engineering strategies to modulate the flux between the C_7_-cyclitol and the amino-deoxyhexose moieties and are able to substantially increase the titer of **1** and decrease the accumulations of the shunt products.

## Results

### Discovery of two main shunt products of acarbose

**Fig. 2:**
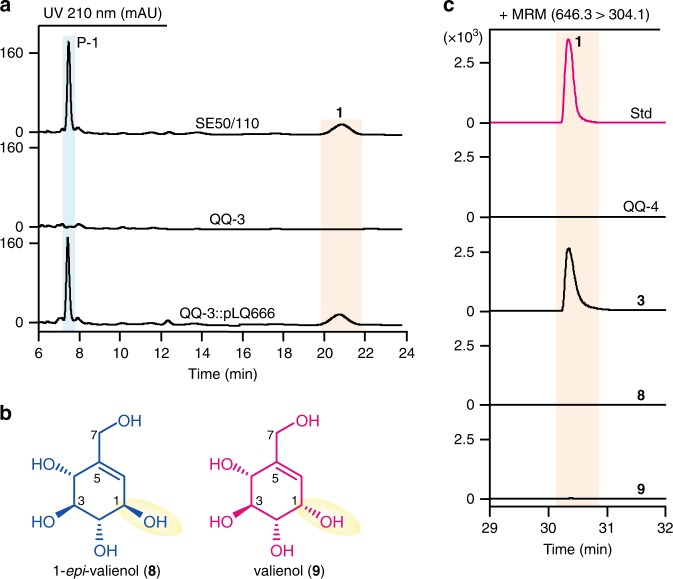
Discovery and identification of shunt products accumulated in the fermentation broth of *Actinoplanes* sp. SE50/110. **a** HPLC profiles of the parent strain *Actinoplanes* sp. SE50/110 (abbreviated as SE50/110), the ∆*acb* mutant QQ-3 and the complemented mutant QQ-3::pLQ666. **b** Structures of 1-*epi*-valienol (**8**) and valienol (**9**). **c** HPLC-QQQ/MS analysis of **1** after feeding with **3** (as positive control), **8** or **9** to the ∆*acbC* mutant QQ-4, and fermentation of QQ-4 without feeding was set as negative control. The standard (abbreviated as std) of **1** was also analyzed. Source data underlying Fig. 2c are provided as a Source Data file.

During the high-performance liquid chromatography (HPLC) analysis of the fermentation broth of *Actinoplanes* sp. SE50/110, we observed a predominant peak (P-1) at a retention time of 7.5 min, while acarbose (**1**) appeared at 20.8 min (Fig. 2a). To examine whether peak P-1 was related to **1**, the whole *acb* cluster (32.2 kb) was deleted in *Actinoplanes* sp. SE50/110, and the mutant was named QQ-3. Both P-1 and **1** disappeared in the fermentation broth of mutant QQ-3, and were restored by trans-complementation of the *acb* cluster cloned on fosmid pLQ666^13^ (Fig. 2a). The simultaneous disappearance and restoration of P-1 and **1** established a correlation between these two peaks.

Through purification and structural elucidation by nuclear magnetic resonance (NMR) spectroscopy, 1-*epi*-valienol (**8**) and valienol (**9**) were confirmed to be present in P-1 (Supplementary Figs. 1–10; Supplementary Table 2; Fig. 2b). To quantify the amounts in fermentation broth, both compounds were derivatized by *N*,*O*-bis(trimethylsilyl)trifluoroacetamide (BSTFA) and analyzed by gas chromatography-quadrupole mass spectrometry (GC-QMS) (Supplementary Fig. 11). Strikingly, **8** and **9** were found to be accumulated at 2.5 g L^−1^ (14.2 mM) and 1.6 g L^−1^ (9.1 mM), respectively, in a 4-day fermentation broth of *Actinoplanes* sp. SE50/110, whereas the titer of **1** was only 3.1 g L^−1^ (4.8 mM). To investigate whether **8** and **9** were hydrolytic products of **1**, **1** was fed to the *acbC*-deleted mutant QQ-4 (Supplementary Fig. 12). However, neither **8** nor **9** were detected, suggesting that they are not derived from **1** (Supplementary Fig. 13).

To examine whether **8** and **9** could be incorporated into **1**, both compounds and 2-*epi*-5-*epi*-valiolone (**3**), the precursor of the C_7_-cyclitol moiety in **1**^19,20^, were fed to the mutant QQ-4. While feeding with **3** resulted in substantial accumulation of **1**, no production of **1** was detected after feeding with **8** or **9** (Fig. 2c), suggesting that **8** and **9** are shunt products, not the precursors of or intermediates in **1** biosynthesis.

### Dephosphorylation of the proposed C_7_-cyclitol intermediates

On the basis of the proposed biosynthetic pathway to **1**^1^ (Fig. 1b), it may be postulated that **8** is derived from 1-*epi*-valienol-7-P (**10**) by a phosphatase. In order to biochemically validate this hypothesis, we prepared compound **10** by incubating **8** with ValC, a known C_7_-cyclitol kinase involved in the biosynthesis of validamycin in *Streptomyces hygroscopicus* var. *jinggangensis* 5008^25^. ValC was able to phosphorylate **8**, and the product **10** (*m/z* = 255.0308 [M-H]^−^) was confirmed. Moreover, ValC was able to catalyze the phosphorylation of **9** to give valienol-7-P (**11**) (Supplementary Figs. 14–16; Supplementary Table 3).

**Fig. 3:**
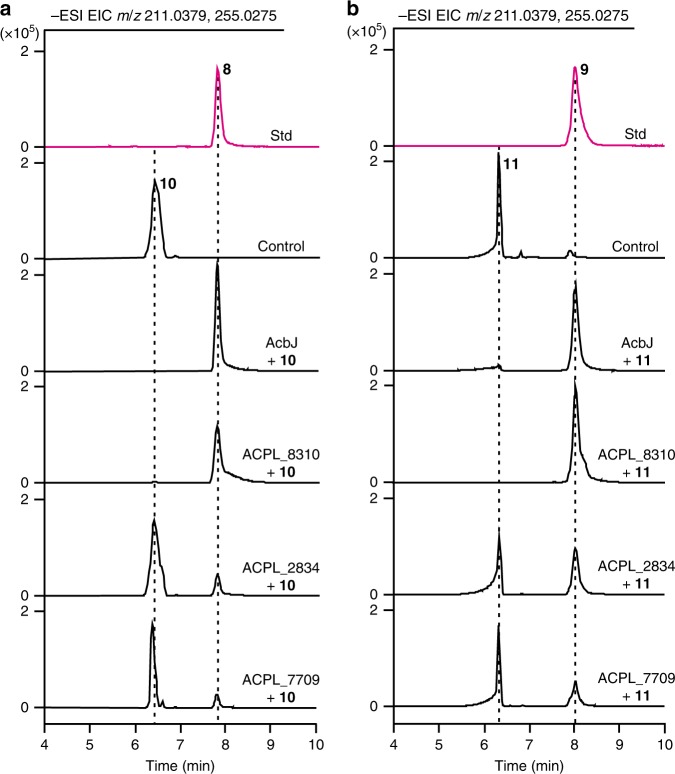
Identification of hydrolases involved in the dephosphorylation of **10** and **11**. **a**, **b** HPLC-TOF/MS analysis of the dephosphorylated products of **10** and **11** catalyzed by AcbJ, ACPL_8310, ACPL_2834, or ACPL_7709 in vitro, and the reaction without enzyme was set as a negative control. The standards (std) of **8** and **9** were also analyzed. All of the chromatograms show the simultaneous extraction of calculated ions *m*/*z* = 211.0379 [M + Cl]^−^ for **8** and **9** and *m*/*z* = 255.0275 [M-H]^−^ for **10** and **11**. Source data are provided as a Source Data file.

With both compounds **10** and **11** in hand, we decided to identify the enzymes that are responsible for the dephosphorylation. The AcbJ protein showed high sequence similarity to hydrolases of the haloacid dehalogenase-like (HAD) superfamily and was initially hypothesized to be responsible for the dephosphorylation of **10** (Supplementary Table 1). The gene was expressed in *Escherichia coli*, and the product was purified. AcbJ was able to efficiently dephosphorylate **10** to **8** (Fig. 3a). To further examine the function of AcbJ in vivo, the *acbJ* gene was inactivated in *Actinoplanes* sp. SE50/110 to give a mutant QQ-5. Compared with *Actinoplanes* sp. SE50/110, QQ-5 showed a 76.4% decrease of phosphatase activity, implying that AcbJ plays a primary role in the dephosphorylation of **10**. In addition, inactivation of *acbJ* abolished the production of **1**, which was partially restored by the trans-complementation of a cloned *acbJ* (Supplementary Fig. 17), indicating that AcbJ is essential for the biosynthesis of **1**. Moreover, the slight decrease in the accumulation of **8** and the residual phosphatase activity suggested that other enzymes, possibly encoded by genes located outside the *acb* cluster, may participate in the dephosphorylation of **10**.

To further identify other potential hydrolases, four candidate proteins (ACPL_8310, ACPL_8309, ACPL_6858, and ACPL_7709) with sequence homology to AcbJ were identified using the BLASTP program^26^, and four HAD superfamily hydrolases (ACPL_2834, ACPL_1897, ACPL_560, and ACPL_7310) with high expression levels were selected according to the transcriptomic analysis of *Actinoplanes* sp. SE50/110^8^ (Supplementary Table 4). Upon incubation of each purified enzyme with **10**, ACPL_8310, ACPL_2834, and ACPL_7709 were found to catalyze the dephosphorylation of **10** (Fig. 3a; Supplementary Fig. 18a), whereas ACPL_8309 showed weak catalytic activity (Supplementary Fig. 19a). Interestingly, **11** was also dephosphorylated to **9** by these five enzymes having activities toward **10** (Fig. 3b; Supplementary Figs. 18b and 19b). Moreover, the deletion of *acbJ* resulted in a 66.9% decrease of the accumulation of **9** (Supplementary Fig. 17c).

### Further clarification of C_7_-cyclitol moiety biosynthesis

**Fig. 4:**
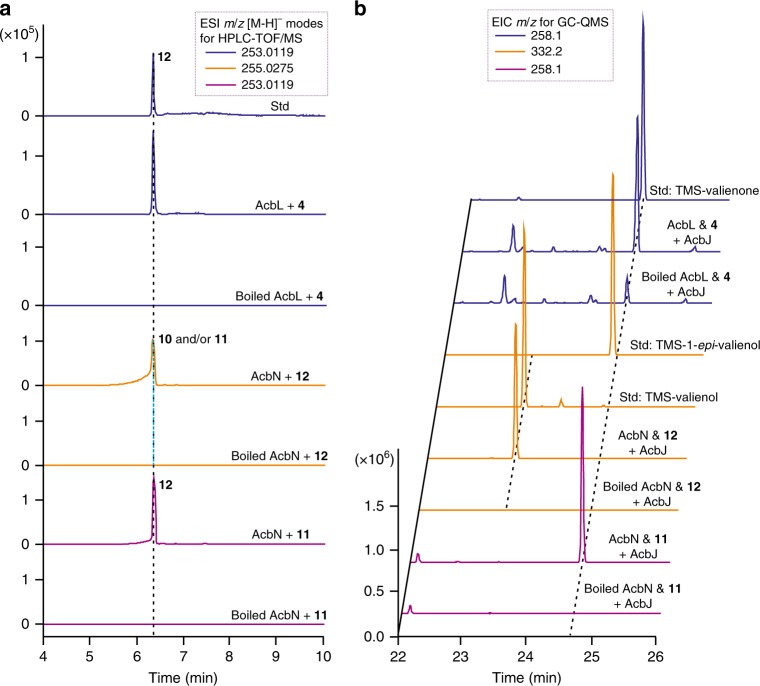
Further clarification of the C_7_-cyclitol moiety biosynthetic pathway by characterizing AcbL- and AcbN-catalyzed conversions. **a** HPLC-TOF/MS analysis of the standard (std) of **12** and the reaction products of AcbL with **4**, boiled AcbL with **4**, AcbN with **12**, boiled AcbN with **12**, AcbN with **11**, and boiled AcbN with **11**. All the chromatograms show the extraction of corresponding calculated ions. **b** GC-QMS confirmation of the reaction products after dephosphorylation by AcbJ. AcbJ was added to the reaction products of AcbL & **4**, boiled AcbL & **4**, AcbN & **12**, boiled AcbN & **12**, AcbN & **11**, and boiled AcbN & **11**. The standards (std) **13**, **8**, and **9** were also analyzed by GC-QMS after derivatization by BSTFA. All the chromatograms show the extraction of corresponding unique product ions. TMS is the abbreviation of trimethyl silicyl. For more details, see also Supplementary Figs. 11, 23. Source data are provided as a Source Data file.

Since ValC is capable of phosphorylating **8** and **9** to **10** and **11** in vitro (Supplementary Fig. 14), respectively, an attempt to recycle **8** and **9** in vivo was performed in mutant QQ-4::*valC* with the gene *valC* integrated in the chromosome. Based on the previously proposed biosynthetic pathway^1^ (Fig. 1b), the recycling of **8** was expected to lead to the production of **1** via the formation of the putative intermediate **10**. However, our feeding experiments with **8** and **9** revealed that only **9** can rescue the production of **1** in the QQ-4::*valC* mutant, suggesting that **11** is a true intermediate for the biosynthesis of **1** (Supplementary Fig. 20). To further clarify the biosynthetic pathway of **1**, we decided to revisit the proposed biochemical function of AcbL, which was annotated as a cyclitol dehydrogenase and proposed to catalyze the reduction of the C-1 carbonyl group of **4** to generate 5-*epi*-valiolol-7-P^1^ (Supplementary Table 1; Fig. 1b). Using sequential catalysis of AcbM and AcbO, compound **4** was prepared from **3**^21^, which was isolated from the fermentation broth of *S. hygroscopicus* var. *jinggangensis* TL01∆*valD*^27^ (Supplementary Fig. 21). Interestingly, when the purified AcbL was incubated with **4** and NADPH (nicotinamide adenine dinucleotide phosphate), a product with a deprotonated molecular ion of *m*/*z* = 253.0121 was detected (Fig. 4a), which was consistent with the calculated *m*/*z* of valienone-7-P (**12**, calc. 253.0119 [M-H]^−^)^25^ (Supplementary Fig. 22a). Furthermore, the reaction mixture was incubated with the phosphatase AcbJ, and the dephosphorylated product was identified as valienone (**13**) (Fig. 4b; Supplementary Figs. 22, 23). AcbL preferred to use NADPH as a cofactor in this reaction (Supplementary Fig. 24). These results indicated that AcbL catalyzes the dehydration of **4** to **12** (Fig. 1b). In addition, a trace amount of **13** (~20 mg L^−1^), likely derived from the intermediate **12** by dephosphorylation, was detected in the fermentation broth of *Actinoplanes* sp. SE50/110. Compound **13** is proposed to be another shunt product in acarbose biosynthesis (Fig. 1b; Supplementary Fig. 25).

AcbN, annotated as a NADPH-dependent oxidoreductase of the short-chain dehydrogenase family (Supplementary Table 1), was proposed to catalyze the reduction of **12**. To test this hypothesis, **12** was prepared from the phosphorylation of **13** by ValC (Supplementary Fig. 26; Supplementary Table 5). When purified AcbN was incubated with **12** and NADPH, a product with a deprotonated molecular ion at *m*/*z* = 255.0319 was detected, which is the same as that of **10** and **11** (calc. 255.0275 [M-H]^−^) (Fig. 4a; Supplementary Fig. 14c). To determine the configuration of AcbN-catalyzed product, AcbJ was subsequently added, and the dephosphorylated product was confirmed to be **9** by GC-QMS (Fig. 4b), suggesting that AcbN catalyzes the conversion of **12** to **11** (Fig. 1b). Furthermore, the product was purified and confirmed as **11** by NMR spectroscopy (Supplementary Figs. 27–30; Supplementary Table 5). NADPH is also the preferential cofactor in this reaction (Supplementary Fig. 31). To test whether AcbN catalyzes the reversible reaction, the purified AcbN was incubated with **11** and NADP^+^. The reaction gave a large amount of **12** (Fig. 4a, b). However, no product was detected in the incubation of AcbN with **10** (Supplementary Fig. 32), suggesting that AcbN specifically recognizes **11** as a substrate. These results indicated that AcbN is responsible for the interconversion between **12** and **11**, and the shunt product **9** is branched from **11** through dephosphorylation. Overall, the five biosynthetic steps from **2** to **11**, leading to the formation of the C_7_-cyclitol moiety of **1**, have been completely illustrated (Fig. 1b; Supplementary Fig. 33).

### Elucidation of the biosynthesis of 1-*epi*-valienol

**Fig. 5:**
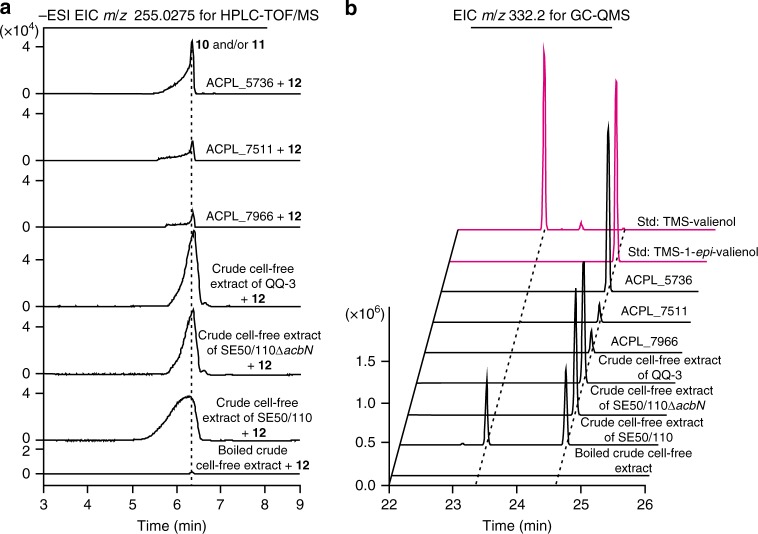
Elucidation of the biosynthetic pathway to the shunt product **8**. **a** HPLC-TOF/MS analysis of the reduction products of **12** by the crude cell-free extract of *Actinoplanes* sp. SE50/110 (abbreviated as SE50/110), SE50/110Δ*acbN* and QQ-3, purified ACPL_7966, ACPL_7511 and ACPL_5736 proteins, and the reaction with boiled crude cell-free extract (BCF) was set as a negative control. All the chromatograms show the extraction of calculated ion *m*/*z* = 255.0275 [M-H]^−^. **b** GC-QMS confirmation of the configuration of the reaction products after dephosphorylation by AcbJ. AcbJ was added to the reaction products of crude cell-free extract of *Actinoplanes* sp. SE50/110, SE50/110Δ*acbN*, and QQ-3, purified ACPL_7966, ACPL_7511, ACPL_5736 proteins, and control reaction. The standards (std) **8** and **9** were also analyzed by GC-QMS after derivatization by BSTFA. For more details, see also Supplementary Fig. 11. All the chromatograms show the extraction of unique product ion *m*/*z* = 332.2. TMS is the abbreviation of trimethyl silicyl. Source data are provided as a Source Data file.

Since AcbN has been shown to specifically catalyze the reduction of **12** to **11**, the shunt product **10** is likely to be derived from **12** by other oxidoreductases. To prove this hypothesis, the gene *acbN* was inactivated in *Actinoplanes* sp. SE50/110. The mutant, SE50/110Δ*acbN*, was not able to produce **9** and **1**, but still produced a large amount of **8**, indicating that **8** and **9** are synthesized via different routes (Supplementary Fig. 34). Subsequently, the crude cell-free extracts (CFEs) of mutant SE50/110Δ*acbN*, QQ-3 (with *acb* cluster deleted), and *Actinoplanes* sp. SE50/110 were individually incubated with **12**, and large amounts of **10** and/or **11** (*m*/*z* = 255.028, calc. 255.0275 [M-H]^−^) were detected (Fig. 5a; Supplementary Fig. 14c). The products were then dephosphorylated with AcbJ and analyzed by GC-QMS, and those from the SE50/110Δ*acbN* and QQ-3 CFE reaction mixtures gave **8**, while that from the *Actinoplanes* sp. SE50/110 CFE reaction mixture gave **8** and **9** (Fig. 5b). Furthermore, the product of the QQ-3 CFE reaction was purified and confirmed as **10** by NMR spectroscopy (Supplementary Figs. 35–39; Supplementary Table 6). Since there was no ORF in the *acb* cluster annotated as an oxidoreductase except for *acbN* (Supplementary Table 1), **12** is deduced to be reduced to **10** by enzymes encoded by genes located outside the *acb* cluster.

To identify the enzymes that are involved in this reaction, total proteins from the cell lysate of QQ-3 were fractionated into 34 fractions by anion exchange chromatography (Supplementary Fig. 40a), and each fraction was assayed for the reductase activity using **12** as substrate. Fractions 15 (F15) and 24 (F24) with highest reductase activity in the presence of NADPH and boiled cell-free extract (BCF), respectively, and F10 with undetectable activity were subjected to proteomic analysis (Supplementary Fig. 40b). The unique putative oxidoreductases with high reliability and abundance (Unique PepCount ≥4) in F15 and F24 were biochemically characterized (Supplementary Fig. 40c; Supplementary Table 7). We discovered that the ACPL_7966, ACPL_7511, and ACPL_5736 proteins in F15 and the ACPL_7667, ACPL_5740, and ACPL_4412 proteins in F24 showed reductase activity (Fig. 5a, b; Supplementary Table 7). These results confirmed that the shunt product **8** is branched from the biosynthetic intermediate **12** by sequential reduction and dephosphorylation (Fig. 1b; Supplementary Fig. 41).

### Minimization of the metabolic flux toward the shunt products

**Fig. 6:**
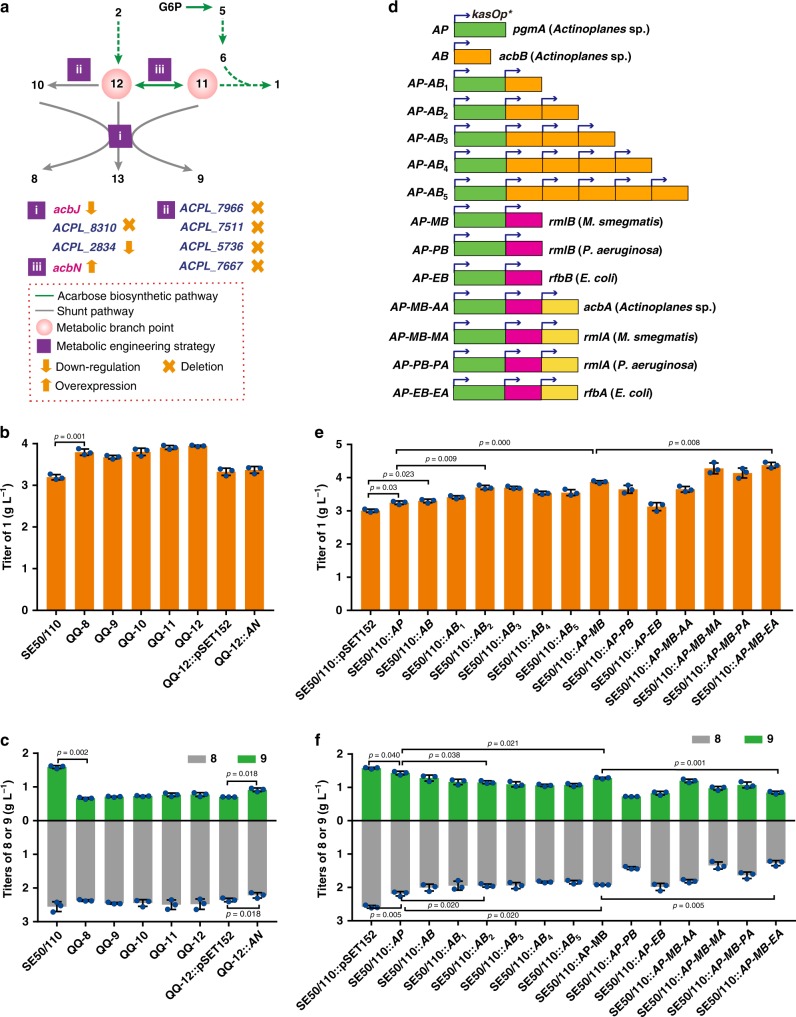
Combined metablic engineering strategies for minimizing the flux toward the shunt products and maximizing the supply of the amino-deoxyhexose moiety. **a** Schematic illustration of the strategies for diverting the metabolic flux of shunt products toward **1**. **b**, **c** The titers of **1**, **8**, and **9** of *Actinoplanes* sp. SE50/110 (abbreviated as SE50/110), QQ-8 (down-regulation of the *acbJ* expression by introducing kasOp*-*acbJ* in the chromosome of QQ-7), QQ-9 (deletion of *ACPL_7966* in QQ-8), QQ-10 (deletion of *ACPL_7511* in QQ-9), QQ-11 (deletion of *ACPL_5736* in QQ-10), QQ-12 (deletion of *ACPL_7667* in QQ-11), QQ-12::pSET152 (as control), and QQ-12::*AN* (overexpression of *acbN* in QQ-12) after fermentation for 4 days. For more details, see also Supplementary Figs. 42–44. **d** Schematic illustration of increasing the biosynthetic capacity of amino-deoxyhexose moiety by stepwise optimization of biosynthetic reactions at gene dosage or enzymatic activity. **e**, **f** The titers of **1**, **8**, and **9** of mutants carrying pSET152 (as control) and its derived plasmids containing cassettes *AP*, *AB*, *AP-AB*_1_, *AP-AB*_2_, *AP-AB*_3_, *AP-AB*_4_, *AP-AB*_5_, *AP-MB*, *AP-PB*, *AP-EB*, *AP-MB-AA*, *AP-MB-MA*, *AP-MB-PA*, or *AP-MB-EA* after fermentation for 4 days. Two-tailed paired *t* tests. Error bars, mean ± SD (*n* = 3 biological replicates). For more details, see also Supplementary Figs. 45, 46. Source data underlying Figs. 6b, c, e, f are provided as a Source Data file.

The parent strain *Actinoplanes* sp. SE50/110 produces 4.1 g L^−1^ (23.3 mM) of **8** and **9** and only 3.1 g L^−1^ (4.8 mM) of **1** after 4-day fermentation (Supplementary Fig. 11), suggesting that the shunt pathways lead to a severe leakage of C_7_-cyclitol intermediates **11** and **12**, which were considered as the key metabolic branch points in the biosynthesis of **1**. Therefore, we proposed that the titer of **1** might be improved by diverting the metabolic flux of the shunt products toward phosphorylated C_7_-cyclitols (**11** and **12**) using metabolic engineering strategies, such as reducing the phosphatase activity, decreasing the conversion of **12** to the shunt product **10**, and enhancing the conversion of **12** to intermediate **11** (Fig. 6a).

Even though AcbJ is the primary phosphatase involved in the dephosphorylation of **10** and **11**, it is essential for the biosynthesis of **1** and therefore cannot be deleted (Supplementary Fig. 17). Alternatively, the expression level of *acbJ* can be tuned down using a weaker promoter. Cassettes of *acbJ* under the control of promoters with different strengths were individually introduced into the *acbJ*-deleted mutant QQ-5. Using the kasOp* promoter, the phosphatase activity was reduced to 54.2% of that of the control strain SE50/110::pSET152, which resulted in increased titer and productivity (ratio of titer of **1** and dry cell weight) of **1** (Supplementary Fig. 42). The cassette kasOp*-*acbJ* was then introduced into the chromosome of QQ-7 (a QQ-5 derivative with the hydrolase gene *ACPL_8310* deleted and the expression of the hydrolase gene *ACPL_2834* down-regulated by replacing its native promoter with a weaker promoter *WVp*) (Supplementary Fig. 43) by homologous recombination. The resulting strain QQ-8 showed an 18.8% and a 30.3% increase in titer and productivity of **1**, respectively (Fig. 6b; Supplementary Fig. 44a). The strain also showed a 7.1% and a 58.3% decrease in the accumulations of **8** and **9**, respectively (Fig. 6c), a 59.8% decrease in phosphatase activity (Supplementary Fig. 44b), a 1.2-fold increase in the total amounts of endogenous **10** and **11** (Supplementary Fig. 44c), and an 8.8% decrease in biomass (Supplementary Fig. 44d).

In order to decrease the conversion of **12** to **10** and promote the conversion of **12** to **11**, *acbN* (*AN*) was overexpressed in QQ-12 (a QQ-8 derivative with successive deletion of the oxidoreductase genes *ACPL_7966*, *ACPL_7511*, *ACPL_5736*, and *ACPL_7667*) (Fig. 6a). Although no obvious titer improvement of **1** and change in biomass were observed, the accumulation of **9** was increased by 30.0%, whereas the accumulation of **8** was decreased by 5.8% (Fig. 6b, c; Supplementary Fig. 44e), suggesting that the overexpression of *acbN* could improve the competing utilization of **12** for the biosynthesis of **11**.

### Maximizing the biosynthesis of amino-deoxyhexose moiety

Even though the abovementioned engineering strategies led to a moderate titer increase of **1**, intracellular phosphorylated C_7_-cyclitols, including the intermediate **11**, were substantially accumulated in the mutant QQ-12 (Fig. 6a–c; Supplementary Fig. 44), implying that an inefficient utilization of these C_7_-cyclitol intermediates by downstream biosynthetic steps has become a major bottleneck during the production of **1** (Fig. 1b). To investigate the rate-limiting factors, transcription levels of genes involved in the biosynthesis of **1** were surveyed by analyzing the transcriptomes of *Actinoplanes* sp. SE50/110 collected at 24, 48, or 72 h during fermentation. The transcription of the phosphoglucomutase gene *pgmA* (*ACPL_2870*) and the 4,6-DH gene *acbB*, critical for the biosynthesis of **6**, was significantly lower than that of other genes in all three transcriptomes (Supplementary Fig. 45a). Therefore, *pgmA* and *acbB* were individually overexpressed under the control of the promoter kasOp* in *Actinoplanes* sp. SE50/110, resulting in improved transcription of the genes by 89.2- and 2.7-fold, respectively (Fig. 6d; Supplementary Fig. 45b–d). This increase in transcription of *pgmA* and *acbB* directly related to 8.1% and 9.7% titer increases of **1** (Fig. 6e), 16.0% and 15.0% productivity increases of **1** (Supplementary Fig. 46a), 15.1% and 22.3% accumulation decreases of **8**, and 9.4% and 20.4% accumulation decreases of **9** (Fig. 6f), respectively, when compared with that of the control strain SE50/110::pSET152. Meanwhile, overexpression of these genes did not show any significant effects on biomass accumulation (Supplementary Fig. 46b). These results suggested that insufficient phosphoglucomutase and 4,6-DH activities are among the limiting factors in the production of **1**.

Since the 2.7-fold improvement of *acbB* transcription was far from ideal, multiple copies of *acbB* (*AB*_*n*_) were co-overexpressed with *pgmA* (*AP*) in *Actinoplanes* sp. SE50/110 (Fig. 6d). The mutant carrying a cassette of *AP*-*AB*_*2*_ had 4.4- and 2.5-fold increases in *acbB* transcription and 4,6-DH activity, respectively, and showed the highest titer and productivity improvement of **1** (23.4% and 29.1%, respectively) (Fig. 6e; Supplementary Fig. 46a, c, d). The cassette also caused decreases in the accumulations of **8** (21.3%) and **9** (27.2%), decreases in the total amounts of endogenous **10** and **11** (10.4%), without any obvious changes in biomass, when compared with that of SE50/110::pSET152 (Fig. 6f; Supplementary Fig. 46b, e).

To avoid any genetic instability of the multiple copies of *acbB*, heterologous genes encoding 4,6-DHs with higher enzymatic activity from *Mycobacterium smegmatis* MC2 155 (*MB*), *Pseudomonas aeruginosa* M18 (*PB*), and *Escherichia coli* K12 W3110 (*EB*)^28–30^ were individually co-expressed with *AP* (Fig. 6d). The introduction of *AP*-*MB* cassette resulted in a 1.9-fold increase in 4,6-DH activity, the highest titer and productivity improvement of **1** (28.9% and 33.5%, respectively), decreases of **8** (25.6%) and **9** (18.8%) accumulations, decreases of the total amounts of endogenous **10** and **11** (23.4%), without any obvious changes in biomass, when compared with that of SE50/110::pSET152 (Fig. 6e, f; Supplementary Fig. 46a, d–g).

To further enhance the competing utilization of precursor **5** for the biosynthesis of **6**, cognate G-1-PT genes^31–33^ were individually co-expressed with *AP*-*MB* in *Actinoplanes* sp. SE50/110 (Fig. 6d). The introduction of the *AP-MB-EA* cassette resulted in a 3.5-fold increase in G-1-PT activity, substantial titer and productivity increases of **1** (45.7% and 56.9%, respectively), decreases in the accumulations of **8** (50.6%) and **9** (46.5%), and decreases in the total amounts of endogenous **10** and **11** (35.2%), and a 6.9% decrease in biomass, when compared with SE50/110::pSET152 (Fig. 6e, f; Supplementary Fig. 46a, e–h).

### Integration of the effective engineering strategies

**Fig. 7:**
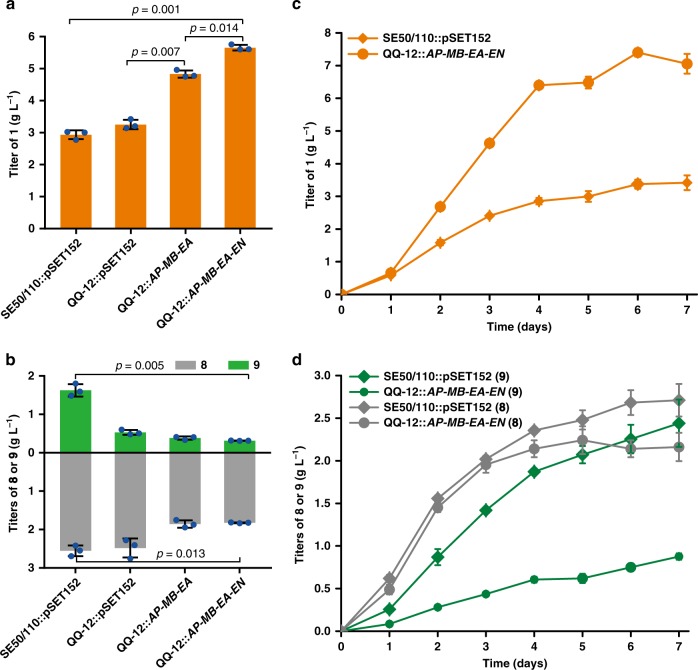
Integration of the effective engineering strategies. **a**, **b** The titers of **1**, **8**, and **9** of SE50/110::pSET152 (as a control), QQ-12::pSET152 (as a control), and QQ-12::*AP-MB-EA* and QQ-12::*AP-MB-EA-AN* after fermentation for 4 days. Two-tailed paired *t* tests. **c**, **d** Fed-batch fermentation of SE50/110::pSET152 (rhombus) and QQ-12::*AP-MB-EA-AN* (circle). Time courses of **1** (orange), **8** (gray), and **9** (green) titers were monitored. Error bars, mean ± SD (*n* = 3 biological replicates). For more details, see also Supplementary Fig. 47. Source data are provided as a Source Data file.

For a final combination of these effective metabolic engineering strategies, the cassettes *AP*-*MB-EA* and *AP*-*MB-EA*-*AN* were individually introduced into the mutant QQ-12. The expression of *AP*-*MB-EA* improved the titer of **1** by 64.8%, which was further increased by 17.0% when *AN* was introduced (Fig. 7a, b; Supplementary Fig. 47). The best engineered strain, QQ-12::*AP*-*MB-EA*-*AN*, produced 7.4 g L^−1^ of **1** in 6-day fed-batch fermentation, representing a 1.2-fold titer and 2.3-fold productivity improvement of **1**, a 20.2% decrease of **8**, and a 67.1% decrease of **9** (Fig. 7c, d). However, an unexpected 33.0% decrease in biomass occurred when compared with the control strain SE50/110::pSET152 (Supplementary Fig. 47c).

## Discussion

Acarbose (**1**) is one of the most widely used anti-diabetic drugs, yet its biosynthesis remained somewhat elusive despite a decades-long clinical application^1^. While previous studies on **1** biosynthesis have revealed the biosynthetic steps from **2** to **4**^20,21^, further downstream, the pathway has yet to be biochemically established. Recently, the formation of a cyclitol moiety in pyralomicin has been proposed to involve dehydration of **4** to **12** and then reduction of **12** to **11**. However, the functions of the proposed enzymes, PrlW (cyclitol oxidoreductase) and PrlV (cyclitol dehydrogenase), have not been experimentally characterized^34^. In the present study, clarification of the functions of AcbL and AcbN through metabolic analysis, chemical incorporation, and biochemical experiments not only made a breakthrough in the study of **1** biosynthesis, but also contributed to a better understanding of the biosynthesis of other C_7_N-aminocyclitols^34–36^.

Accordingly, the subsequent biosynthetic steps of C_7_-cyclitol moiety proposed in the previous study^1^ was updated with phosphorylation of **11** to valienol-1,7-diP and nucleotidylation of the product to NDP-valienol-7-P (Fig. 1b). Although AcbJ was demonstrated to be essential for the biosynthesis of **1** through gene deletion in *Actinoplanes* sp. SE50/110 (Supplementary Fig. 17), its definite function is still unclear. However, since AcbJ can catalyze the dephosphorylation of **11**, we speculate that it is responsible for the hydrolysis of the C-7 phosphate group of the proposed intermediates valienol-1,7-diP, NDP-valienol-7-P, or dTDP-acarviose-7-P (Fig. 1b). This will be a subject of our future investigation related to **1** biosynthesis.

In line with the results of previous feeding studies with ^13^C-labeled **8** and **13**, in which none of these compounds were incorporated into **1**^19^, our work showed that both compounds are shunt products branched from the biosynthetic intermediate **12** (Fig. 1b; Supplementary Fig. 33). Although **8** could be converted to **10** by kinase(s) in the CFE of *Actinoplanes* sp. SE50/110^37^, the reaction does not seem to be part of **1** biosynthesis or a salvage pathway to **1**, as our study showed that **10** is also a shunt product directly derived from **12** (Figs. 1b and 5). It has also been reported that the CFE of *Actinoplanes* sp. SE50/110 showed weak kinase activity toward **9** and presumably generated a low amount of **11**^37^. However, the production of **1** was not detected after feeding QQ-4 with **9** in our study, except when the C_7_-cyclitol kinase gene *valC* was introduced in the chromosome as in QQ-4::*valC* (Fig. 2c; Supplementary Fig. 20), suggesting that the weak kinase activity might be insufficient to produce detectable **1**.

The identified shunt products **8**, **9**, **10**, and **13** are branched from the metabolic intermediates **11** and **12** (Fig. 1b). The substrate promiscuities of the involved phosphatases and oxidoreductases control the branch points and thereby establish complicated crosstalks between the shunt pathways, **1** biosynthesis, and primary metabolism. In this network, AcbJ is a crucial switch that controls the metabolic flux toward **1** and shunt products. Likewise, the putative hydrolase (ACPL_8310), β-phosphoglucomutase (ACPL_2834), trehalose 6-phosphate phosphatase (ACPL_7709), gfo/Idh/MocA family oxidoreductase (ACPL_7966), inositol 2-dehydrogenase (ACPL_7511), glucose-6-phosphate 1-dehydrogenase (ACPL_5736), and acyl-CoA dehydrogenase (ACPL_7667) play important roles in primary metabolism and contribute to the biosynthesis of the shunt products as well. Accordingly, the similar shunt pathways were also speculated to exist in the biosynthetic pathways of other C_7_N-aminocyclitols^34,35^.

The high intracellular levels of phosphorylated metabolites are usually toxic and cause growth inhibition^38,39^. This is supported by the fact that deletion of *acbJ* resulted in a significant increase in intracellular phosphorylated C_7_-cyclitols (**10** and **11**) and obvious decrease in the biomass compared with that of *Actinoplanes* sp. SE50/110 (Supplementary Fig. 17). Meanwhile, the growth inhibition was gradually alleviated along with the increase in phosphatase activities and the decrease in total amounts of endogenous **10** and **11** due to the trans-complementation of *acbJ* under the control of promoters with different strengths (Supplementary Fig. 42). These evidences suggest that dephosphorylation of accumulated phosphorylated C_7_-cyclitols (**10** and **11**) by the phosphatases and efflux of the dephosphorylated products (**8** and **9**) are parts of a detoxification process in cells. However, it is still unclear why the deletion of *acbJ* resulted in a substantial decrease in the accumulation of **9**, but had no obvious effect on that of **8** (Supplementary Fig. 17c). We proposed that the accumulated intermediate **11**, caused by the blockage of **1** biosynthesis and low dephosphorylation activity in the *acbJ* deletion mutant, is reversely oxidized to **12** by AcbN and then reduced to **10** by the reductases (Fig. 1b). This may contribute to the relatively constant concentration of **8**. However, this hypothesis needs to be further confirmed through quantification of intracellular **10** and **11**.

We found that an imbalanced metabolic flux caused by the insufficient biosynthesis of amino-deoxyhexose moiety is another crucial factor for the leakage of C_7_-cyclitol intermediates. Therefore, employment of multiple strategies to reduce the metabolic flux to shunt products resulted in only a moderate increase of **1**, but caused a substantial increase in the intracellular concentrations of phosphorylated C_7_-cyclitols (Fig. 6a, b; Supplementary Fig. 44c). This agrees well with the result of a recent study, in which overexpression of *acbC* in *Actinoplanes* sp. SE50/110 led to an increase in intracellular **11**, rather than the production of **1**^40^.

Moreover, the low transcription of *acbB* seemed to be a general phenomenon, which was also found in the transcriptome analysis of *Actinoplanes* sp. SE50/110 cultured in different fermentation media^9^. In our work, two synthetic biology strategies were performed to adjust the activity of 4,6-DH: (1) balancing the transcription levels of *acbB* and other genes in the *acb* cluster by titrating gene copy numbers; (2) improving the 4,6-DH activity by recruiting heterologous enzymes with higher catalytic activities from *M. smegmatis*, *E. coli*, or *P. aeruginosa*^28–30^. Both strategies resulted in successful improvement of the biosynthetic capacity of amino-deoxyhexose moiety (Fig. 6e; Supplementary Fig. 46). The substantial titer improvement of **1** with highly efficient heterologous G-1-PTs^31–33^ further demonstrated that overexpression of enzymes that divert primary metabolites into secondary metabolism is a useful strategy^41^ (Fig. 6e). Therefore, these results demonstrated the potential of optimization of the biosynthetic pathway by employing various highly efficient heterogenous isoenzymes.

However, due to the promiscuous activities of the already identified and even more unidentified hydrolases and oxidoreductases^38,42–45^ involved in the accumulation of shunt products, ~58.4% shunt products (12.2 mM **8** and 4.3 mM **9**) still remained in the fermentation broth of the best engineered strain. Meanwhile, the 11.6 mM decreased accumulations of shunt products just resulted in a 6.2 mM increase of **1** titer (Fig. 7c, d), suggesting that the other 5.4 mM C_7_-cyclitol intermediates remain in the cell or are utilized by other unknown competing metabolic pathways (Supplementary Fig. 47d). These results implied the potential in maximizing the utilization of the C_7_-cyclitol intermediates for the titer improvement of **1**, with a better understanding of biosynthesis and expanded genetic toolbox for rational engineering^46,47^. In addition, the titer of **1** of the best engineered strain is promising for further improvement using optimized fermentation strategies^48–50^.

Taken together, systematic metabolic engineering strategies, minimizing the metabolic flux towards shunt products and maximizing the supply of amino-deoxyhexose moiety to balance the flux with C_7_-cyclitol moiety, were employed to promote the effective utilization of C_7_-cyclitol intermediates for the production of **1**. Our effort at uncovering and fine-tuning the biosynthetic network of **1** ensures an opportunity on understanding the biosynthesis of other C_7_N-aminocyclitols, diagnosing metabolic bottlenecks and enhancing yields through pathway engineering^17,24,34,35,51^.

## Methods

### Strains and their culture conditions

Strains, plasmids, and primers used in this study are listed in Supplementary Table 8, Supplementary Table 9, and Supplementary Data 1, respectively.

*Escherichia coli* DH10B was used for gene cloning, ET12567(pUZ8002) was used for the intergeneric conjugation between *E. coli* and mycelia of *Actinoplanes* sp., and BL21(DE3) was used for the heterologous expression of proteins. *Escherichia coli* were cultured with Luria-Bertani (LB) broth or on LB agar plates at 37 °C. *Actinoplanes* sp. SE50/110 and its derivatives were cultivated on STY agar medium (sucrose 3%, tryptone 0.5%, yeast extract 0.5%, casin hydrolysate 0.1%, K_2_HPO_4_·3H_2_O 0.1%, KCl 0.05%, FeSO_4_ 0.005%, agar 2%, pH 7.2) at 30 °C for 2–3 days, and then inoculated into 30 mL of SM broth (glucose 1.5%, maltose 1%, K_2_HPO_4_·3H_2_O 0.1%, glycerol 1%, malt extract 1%, tryptone 0.5%, yeast extract 0.5%, casin hydrolysate 0.1%, pH 7.2) in 250 mL baffled flasks for about 36 h on rotary shaker (30 °C, 220 r.p.m.). The medium for the seed culture contains 1.5% maltose, 1% glucose, 4% soya flour, 1% glycerol, 1% soluble starch, and 0.2% CaCO_3_, pH 7.2. The fermentation medium contains 5% maltose, 3% glucose, 1% soya flour, 0.3% glutamate, 0.3% yeast (Angel Yeast Co., Ltd), 0.1% K_2_HPO_4_·3H_2_O, 0.05% FeCl_3_, and 0.25% CaCO_3_, pH 7.2. When needed, antibiotics were added to final concentrations of 50 mg L^−1^ for apramycin and kanamycin, and 25 mg L^−1^ for chloramphenicol.

To assess the production of **1** and biomass of *Actinoplanes* sp. SE50/110 and its derivatives, 4-mL of SM culture was transferred to 40 mL of seed medium in 250 mL baffled flask and cultivated for 28–30 h on rotary shaker (30 °C, 220 r.p.m.). Then, 7.5-mL seed culture was inoculated to 50 mL of fermentation medium in 250 mL baffled flask and cultivated for another 4 days. Additionally, 1 g glucose was added to each flask on the second day. Then, the production of **1** and the biomass (dry cell weight) was analyzed after 4-day fermentation.

To assess the potential of the engineered strains, the fed-batch fermentation was conducted in baffled flasks using feeding strategy that 0.5 g glucose was added at 24 h, and then 0.75 g glucose and 0.125 g maltose were added every 24 h. The fermentation was continued for 7 days on a rotary shaker (30 °C, 220 r.p.m.).

### The involved chemicals and their sources

Synthesis of oligonucleotide primers and sequencing of PCR products were performed by Tsingke Biological Technology. 5-Flucytosine was purchased from Adamas. The derivatization reagent BSTFA:TMCS (99:1) was purchased from Sigma-Aldrich. The standards of valienol (**9**) (Supplementary Fig. 48a) and valienone (**13**) were synthesized by WuXi Apptec Co., Ltd. The standard of 1-*epi*-valienol (**8**) was purified from the fermentation broth of SE50/110Δ*acbN* and confirmed by NMR spectroscopy (Supplementary Fig. 48b). The standards of dTDP-D-glucose and dTDP-4-keto-6-deoxy-D-glucose were purchased from Carbosynth China Ltd. The molecular biology reagents were purchased from Thermo Fisher Scientific and Takara. Other biochemical reagents were purchased from Sinopharm Chemical Reagent Co., Ltd., Oxoid, Sangon Biotech, and Sigma-Aldrich.

### Manipulation of genes

For the deletion of the *acb* cluster (containing 32.2-kb region of DNA from *acbZ* to the upstream region of *acbD*), homologous flanking sequences (*acb*-L and *acb*-R) were amplified with corresponding primers (Supplementary Data 1) from the genome of *Actinoplanes* sp. SE50/110 (GenBank accession no. CP003170.1) and cloned into the *Actinomycete–E. coli* shuttle vector pLQ752 to give pLQ759, which was further verified by sequencing. Plasmids pLQ760, pLQ761, pLQ762, pLQ765, pLQ766, pLQ767, pLQ768, and pLQ769 were similarly constructed for the in-frame deletions of *acbC*, *acbJ*, *ACPL_8310*, *ACPL_7966*, *ACPL_7511*, *ACPL_5736*, *ACPL_7667*, and *acbN*, respectively.

To reduce the transcription of *ACPL_2834*, two homologous flanking sequences (*2834*d-L and *2834*d-R) and *WV* promoter (*WVp*) (Supplementary Data 2) of the *acb* cluster were amplified from the genome of *Actinoplanes* sp. SE50/110. The *WVp* and *2834*d-R were ligated by overlapping PCR and cloned into pLQ752 together with *2834*d-L to generate pLQ763. For reducing the *acbJ* transcription, two homologous flanking sequences (*acbJd*-L and *acbJd*-R) were amplified from the genome of *Actinoplanes* sp. SE50/110 and cloned into pLQ752. Subsequently, the kasOp*-*acbJ* was amplified from pSET152-kasOp*-*acbJ* and inserted between these two homologous flanking sequences to generate pLQ764.

The recombinant plasmids were transferred to ET12567(pUZ8002) and then introduced into *Actinoplanes* sp. SE50/110 by intergeneric conjugation. The methods of intergeneric conjugation, and mutant construction and selection were described in the Supplementary Method. The schematic representation of gene deletions and insertions in the chromosome and the verification of the mutants by PCR were shown in Supplementary Figs. 49–54. Meanwhile, the corresponding PCR products were further verified by DNA sequencing.

### Overexpression or complementation of genes

For the expression of single gene, promoters kasOp* and ermEp*, and endogenous promoters *gapAp*, *groLp*, and *WVp* were, respectively, amplified from pDR-4-K*, pIB139, and the genome of *Actinoplanes* sp. SE50/110 with corresponding primers (Supplementary Data 1–4)^52–54^. Meanwhile, gene *valC* was amplified from the genome of *S. hygroscopicus* var. *jinggangensis* 5008 (GenBank accession no. CP003275.1)^25^, and genes *acbJ*, *acbN*, *acbA*, *acbB*, and *pgmA* were amplified from the genome of *Actinoplanes* sp. SE50/110. The corresponding promoters and genes were ligated by overlapping PCR, cloned into pSET152^55^, and verified by sequencing.

For the construction of pSET152-*AP-AB*_*n*_, the kasOp*-*pgmA* (*AP*) was amplified using primers kasOp*-*Xba*I-F/*pgmA*-*Afl*II-R and then cloned into the pSET152-M to generate pSET152-*AP*. Subsequently, fragments of kasOp*-*acbB* (*AB*) with different flanking restriction sites of *Afl*II/*Bgl*II, *Bgl*II/*Kpn*I, *Kpn*I/*Nde*I, *Nde*I/*Sca*I, or *Sca*I/*Mfe*I were individually amplified using corresponding primers (Supplementary Data 1) and successively cloned into pSET152-*AP* to generate pSET152-*AP-AB*_1_, pSET152-*AP-AB*_2_, pSET152-*AP-AB*_3_, pSET152-*AP-AB*_4_, or pSET152-*AP-AB*_5_.

For the co-expression of the heterogenous genes encoding 4,6-DHs, they were, respectively, amplified from the genomes of *M. smegmatis* MC2 155 (*MB*, GenBank accession no. CP000480.1), *P. aeruginosa* M18 (*PB*, GenBank accession no. CP002496.1), and *E. coli* K12 W3110 (*EB*, GenBank accession no. AP009048.1) using corresponding primers, ligated with the kasOp* promoter by overlapping PCR, and then cloned into pSET152-*AP* to generate pSET152-*AP*-*MB*, pSET152-*AP*-*PB*, and pSET152-*AP*-*EB*. Subsequently, cognate G-1-PT genes *AA*, *MA*, *PA*, and *EA* were obtained in a similar way and further inserted into pSET152-*AP*-*MB* to generate pSET152-*AP*-*MB-AA*, pSET152-*AP*-*MB-MA*, pSET152-*AP*-*MB-PA*, and pSET152-*AP*-*MB-EA*, respectively.

The recombinant plasmids were transferred to ET12567(pUZ8002) and then introduced into *Actinoplanes* sp. SE50/110 and the corresponding derivatives by intergeneric conjugation. The exconjugants were selected, cultured, verified by PCR, and the corresponding PCR products were further verified by DNA sequencing (Supplementary Method 1).

### Purification and identification of compounds in P-1

For the preparation of compounds in P-1 (Fig. 2a), 1 L of fermentation broth of *Actinoplanes* sp. SE50/110 was harvested by centrifugation. The supernatant was adjusted to pH 3.0–4.0 with oxalic acid. After removing the precipitate by centrifugation, the supernatant was adjusted with ammonium hydroxide to pH 7.0 and mixed with anion exchange resin D201 (1 g for 10 mL broth) for about 1 h to remove pigments. Then, the mixture was filtered through Whatman filter paper and concentrated to 200 mL using vacuum evaporator at 40 °C. About 10-mL concentrated liquor was subjected to column filled with pre-treated aminopropyl-functionalized silica gel each time, and eluted with acetonitrile/water (70:30, v/v) to many fractions. As monitored by HPLC, fractions containing the target compounds were pooled, concentrated, and freeze-dried.

For subsequent purification, the powder was dissolved in 5-mL Milli-Q water and then subjected to Sephadex LH-20 (2 × 200 cm) column twice. The column was eluted with methanol/water (30:70, v/v), and fractions containing the target compounds were pooled. After concentration, the sample was separated again on Sephadex LH-20 column, and maltose was removed. For the removal of glucose residue, the sample was dissolved in water and treated with hexokinase at 30 °C for 1 h. The reaction product was freeze-dried, dissolved in 2–3 mL of Milli-Q water, and separated twice on Sephadex LH-20 column. Further separation was performed on analytical HPLC (Agilent series 1260, Agilent Technologies, USA) with Agilent ZORBAX SB-Aq (4.6 × 250 mm^2^, particle size 5 μm) at a flow rate of 1 mL min^−1^ using an elution buffer of water/acetonitrile (99.5:0.5, v/v). The purified compounds were freeze-dried.

Purified compounds were dissolved in D_2_O, and the one-dimensional (1D) (^1^H, ^13^C, and distortionless enhancement by polarization transfer) and 2D (^1^H-^1^H correlation spectroscopy [COSY], heteronuclear single-quantum correlation spectroscopy [HSQC], hetero-nuclear multiple-bond correlation spectroscopy [HMBC], and nuclear overhauser effect [NOESY]) NMR spectra were collected in D_2_O at 600 MHz (^1^H NMR) and 150 MHz (^13^C NMR) on Bruker Avance III 600 spectrometer (magnetic field strength with 14.09 T). The NMR data processing was performed using the software of MestReNova 9.0.1.

### Expression and purification of proteins

For the expression of ValC, gene *valC* was amplified from the genome of *S. hygroscopicus* var. *jinggangensis* 5008 and cloned into pET-30a^56^ to generate pET30a-*valC*. BL21(DE3) with the recombinant plasmid was cultured in LB medium containing kanamycin (50 µg/mL) at 37 °C to OD_600 nm_ of 0.6–0.8. Protein expression was induced with IPTG (isopropyl β-D-1-thiogalactopyranosid) (0.4 mM) at 16 °C for 12–16 h. Cells were harvested by centrifugation, re-suspended in buffer TN (25 mM Tris-HCl, pH 8.0, 300 mM NaCl), and lysed by sonication. The supernatant was separated from the cell debris by centrifugation at 15,777 × *g* for 45 min at 4 °C, and passed through pre-treated nickel-affinity chromatography. After pre-wash of the column with 50-mL washing buffer (25 mM Tris-HCl, pH 8.0, 300 mM NaCl, 25 mM imidazole), the target protein was eluted with elution buffer (25 mM Tris-HCl, pH 8.0, 300 mM NaCl, 250 mM imidazole). The purified protein was concentrated and exchanged with buffer TN using 10 kDa concentrator (Millipore Amicon Ultra), and checked by sodium dodecyl sulfate-polyacrylamide gel electrophoresis, and its concentration was determined by Bradford Protein Assay Kit (Sangon Biotech). The protein was used for enzymatic assay or stored in 10% glycerol at −80 °C.

The pET30a-*acbM*, pET30a-*acbN*, pET30a-*acbO*, and pET30a-*acbL* were expressed in BL21(DE3)/pGro7, which were cultured in LB supplemented with kanamycin (50 mg L^−1^), chloramphenicol (25 mg L^−1^), and l-arabinose (2 g L^−1^) in the beginning. Additionally, the procedures of expression and purification of these proteins were similar with that for ValC.

### Preparation and characterization of phosphorylated products

For the preparation of **10**, a 5-mL reaction mixture containing 25 mM Tris-HCl (pH 7.5), 10 mM MgCl_2_, 10 mM NH_4_Cl, 10 mM ATP, 4.5 mM **8**, and 6 µM ValC was incubated at 30 °C for 6 h. The reaction was stopped by adding two volumes of methanol followed with vigorous vortex to denature the proteins. The mixture was centrifuged at 15,777 × *g* for 10 min, and the supernatant was concentrated and freeze-dried. The powder was dissolved in 1-mL water and subjected to Sephadex LH-20 column. Fractions containing target compound were pooled. The ^1^H and ^13^C NMR spectra of the purified compound were collected in D_2_O at 600/700 MHz (^1^H NMR) and 150/175 MHz (^13^C NMR) on Bruker Avance III 600/AVANCE NEO 700 spectrometers (magnetic field strength with 14.09/16.44 T). The same method was used to prepare, purify, and characterize **11** and **12**.

### HPLC analysis of the titer of **1**

The supernatant of fermentation broth was obtained by centrifugation at 15,777 × *g* for 10 min and diluted for 5 folds. The concentration of **1** was analyzed by HPLC (Agilent series 1260, Agilent Technologies, USA) with Agilent ZORBAX NH_2_ column (4.6 × 250 mm^2^, particle size 5 μm) using an elution buffer of acetonitrile/phosphate buffer (65:35, v/v) at a flow rate of 1 mL min^−1^ and detected at 210 nm. The titer of **1** was calculated according to the corresponding standard curves and processed by the GraphPad Prism 7 and Origin 8.1 softwares.

### HPLC-TOF/MS analysis of the related compounds

The enzymatic reaction mixtures and related compounds were analyzed by HPLC-time-of-flight/mass spectrometry (HPLC-TOF/MS) (Agilent 1290-6500 Q-TOF) with Agilent ZORBAX SB-Aq (4.6 × 250 mm^2^, particle size 5 μm) at a flow rate of 0.4 mL min^−1^ using an elution buffer composed of (A) Milli-Q water and (B) methanol with the gradient elution procedures: 0–20 min, 0.5% B; 20–22 min, 0.5% B to 95% B; 22–27 min, 95% B; 27–28 min, 95% B to 0.5% B; 28–38 min, 0.5% B. The related compounds were detected in negative ion mode and their calculated *m/z* were listed in Supplementary Table 10.

### HPLC-QQQ/MS analysis of the related compounds

Production of **1** in feeding experiments, concentrations of endogenous **10** and **11**, and the products of enzymatic reactions were quantified by HPLC-triple quadrupole/mass spectrometry (HPLC-QQQ/MS) (Agilent 1100 series LC/MSD Trap System), working in the Multiple Reaction Monitoring model by employment of an unique precursor ion-to-product ion for each analyte to achieve highest selectivity and sensitivity. The parameters of polarity, precursor ion, product ion, fragmentor, and collision energy for the related compounds were individually optimized and listed in Supplementary Table 10. HPLC method was the same as that of HPLC-TOF/MS.

### GC-QMS analysis of the three shunt products

Twenty-microliter samples of pre-treated reaction mixtures or 50-fold diluted fermentation broth were dried in vacuum and dissolved in equal volume of pyridine. Ten-microliter solution was mixed with 20 µL of derivatization reagent (BSTFA:TMCS = 99:1) in the vitreous insert and put in a sealed GC vial rapidly, and then incubated at 70 °C for 45 min. The derived samples were analyzed by GC-QMS (Agilent Technologies, 6850 GC/5975C MSD) equipped with a capillary column (HP-5 ms; 30 m × 0.25 mm^2^). The oven program was as follows: 70 °C for 2 min, ramp to 130 °C at 5 °C/min, ramp to 180 °C at 10 °C/min, ramp to 285 °C at 5 °C/min, held for 5 min. The standards of **8**, **9**, and **13** were derivatized to TMS-1-*epi*-valienol, TMS-valienol, and TMS-valienone, respectively, by BSTFA (TMS is the abbreviation of trimethyl silicyl) and analyzed by GC-QMS. Their mass spectra were showed in Supplementary Table 10, Supplementary Fig. 11, and Supplementary Fig. 23. The same method was used for the quantitative analysis of **8** and **9** via GC-QQQ/MS (Thermo Scientific, TSQ 8000) by tracing their unique product ion *m*/*z* = 332.2. Their titers were calculated according to the corresponding standard curves and processed by the GraphPad Prism 7 and Origin 8.1 softwares.

### Analysis of biomass and time courses of cell growth

For analysis of biomass, the mycelia were harvested from 1-mL fermentation culture by centrifugation, washed with 0.25 M hydrochloric acid, and dried at 105 °C in oven until constant weight reached. The dry cell weight was detected with precision balance. To obtain the time courses of cell growth, 1-mL fermentation culture was sampled every 24 h, and the dry cell weight was measured. The fermentation lasted for 7 days, and 1 g glucose was added to each flask on days 2 and 4.

### Significance analysis of the amounts of **1** and shunt products

Two-tailed paired *t* tests were used for the statistical analysis, which was conducted by SPSS (Statistical Product and Service Solutions) software^57^. The software (IBM SPSS Statistics 20) compared the control groups with the corresponding experimental groups and gave *p* values. *P* < 0.05, *p* < 0.01, and *p* < 0.001 are defined as significant, moderately significant, and highly significant, respectively.

### In vitro enzymatic assays

For the enzymatic assay of ValC, a 20-µL reaction mixture, containing 25 mM Tris-HCl (pH 7.5), 10 mM MgCl_2_, 10 mM NH_4_Cl, 10 mM ATP, 2.5 mM **8** or **9**, and 6 µM enzyme, was incubated at 30 °C for 1 h. The reaction mixture with boiled ValC was set as control. The reaction was stopped by adding two volumes of methanol, followed by vigorous vortex to denature the proteins. The mixture was centrifuged at 15,777 × *g* for 20 min, and the supernatant was subjected to HPLC-TOF/MS analysis.

For the enzymatic assay of phosphatase AcbJ, a 20-µL reaction mixture, containing 25 mM Tris-HCl (pH 7.5), 10 mM MgCl_2_, 10 mM NH_4_Cl, 1.25 mM phosphorylated compounds (**10**, **11**, or **12**), and 3 µM enzyme, was incubated at 30 °C for 0.5 or 6 h. The reaction mixtures with boiled AcbJ and without enzyme were set as controls. The 6-h reaction mixture was analyzed by HPLC-TOF/MS and GC-QMS. The concentrations of dephosphorylated compounds (**8**, **9**, or **13**) in 0.5-h reaction mixture were quantified by GC-QQQ/MS to calculate enzymatic activity. One enzyme unit (U) is defined as the amount of dephosphorylated compound in nanomole produced in 1 min. The same procedure was used to analyze the enzymatic activities of phosphatases ACPL_8310, ACPL_2834, and ACPL_7709.

For the enzymatic assay of AcbL, substrate **3** was purified from the fermentation broth of TL01Δ*valD* following the separation procedure of **8** or **9**. A total of 2.5 mM of **3** was added to a reaction mixture, containing 25 mM Tris-HCl (pH 7.5), 10 mM MgCl_2_, 10 mM NH_4_Cl, 20 mM ATP, 1 mM, dithiothreitol (DTT), and 3 µM AcbM, and incubated at 30 °C for 12 h. The freeze-dried reaction residue was then dissolved in water with 1 mM DTT and 3 µM AcbO and incubated at 30 °C for 12 h to prepare **4**. The reaction was stopped by adding two volumes of methanol, and the supernatant was concentrated by rotary evaporation and freeze-dried. The powder was dissolved in equal volume of water and incubated with 10 µM Zn^2+^, 1 mM DTT, 1 mM NADPH, and 3 µM AcbL at 30 °C for 6 h. The reaction mixture with boiled AcbL was set as control. A portion of the reaction mixture was analyzed by HPLC-TOF/MS, and the remaining mixture was further incubated with 3 µM AcbJ for 2 h and detected by GC-QMS.

For the enzymatic assay of AcbN, reaction mixture, containing 25 mM Tris-HCl (pH 7.5), 10 mM MgCl_2_, 1.25 mM **12**, 1 mM NADPH, and 3 µM enzyme, was incubated at 30 °C for 6 h. The reaction mixture with boiled AcbN was set as control. A portion of the reaction mixture was analyzed by HPLC-TOF/MS, and the remaining mixture was further incubated with 3 µM AcbJ for 2 h and detected by GC-QMS. The similar procedure was used to characterize the reverse reaction by replacing **12** and NADPH with **11** and NADP^+^. To identify the optimal cofactors for the reversible conversion between **12** and **11**, the oxidation of NADH or NADPH and the reduction of NAD^+^ or NADP^+^ in the corresponding reaction mixtures were monitored by a spectrophotometer (UV-2550, Shimadzu) at *A*_340 nm_ for 6 min.

To examine whether the CFEs of *Actinoplanes* sp. SE50/110, SE50/110Δ*acbN* and QQ-3 catalyze the reduction of **12**, cells were harvested from 2-day fermentation broth through centrifugation, washed twice, re-suspended in buffer TN, and lysed by sonication. The soluble proteins were separated from the cell debris by centrifugation at 15,777 × *g* for 10 min at 4 °C. Subsequently, the reaction mixture containing 25 mM Tris-HCl (pH 7.5), 10 mM MgCl_2_, 1.25 mM **12**, 1 mM NADPH, and 15 g L^−1^ of crude enzymes was incubated at 30 °C for 6 h. The reaction mixtures with boiled CFEs were set as controls. A portion of reaction mixture was analyzed by HPLC-TOF/MS, and the remaining mixture was further incubated with 3 µM AcbJ for 2 h and detected by GC-QMS.

In addition, the method for enzymatic assay of the putative NADPH-dependent oxidoreductases was similar with that of AcbN. However, for the oxidoreductases using unknown or unstable cofactors, they were incubated with **12** in the presence of the supernatant of BCF of QQ-3. One enzyme unit (U) is defined as the amount of **11** in nanomole produced in 1 min.

### Purification and identification of the reaction products

For the purification of AcbN-catalyzed product, a 5-mL reaction mixture containing 25 mM Tris-HCl (pH 7.5), 10 mM MgCl_2_, 1.25 mM **12**, 5 mM NADPH, and 3 µM AcbN was incubated at 30 °C for 6 h. The reaction was stopped by adding two volumes of methanol followed with vigorous vortex to denature the proteins. The mixture was centrifuged at 15,777 × *g* for 10 min, and the supernatant was concentrated and freeze-dried. The powder was dissolved in 1-mL water and subjected to Sephadex LH-20 column. Fractions containing target compound were pooled. The same method was used to purify the reaction product catalyzed by the CFE of QQ-3 using **12** as substrate. The 1D (^1^H and ^13^C) and 2D (^1^H-^1^H COSY, HSQC, HMBC, and NOESY) NMR spectra of the purified compounds were collected in D_2_O at 700 MHz (^1^H NMR) and 175 MHz (^13^C NMR) on Bruker AVANCE NEO 700 spectrometer (magnetic field strength with 16.44 T).

### Feeding experiments

For the feeding experiments, 150 µL of seed cultures of *Actinoplanes* sp. SE50/110 or the derivatives were inoculated to 850-µL fermentation medium in 24-well deep well plates by adding 5 mg L^−1^ of **8** or **9**. After 48-h incubation on rotary microplate shaker (30 °C, 250 r.p.m.) (Parallel-bioreactor, China), the supernatant of the fermentation broth was diluted for 2 folds and analyzed by HPLC-QQQ/MS.

### The workflow for identification of the oxidoreductases

To prepare the cellular proteins of QQ-3, cells were harvested from 2-day fermentation broth by centrifugation, washed twice, re-suspended in buffer T (25 mM Tris-HCl, pH 8.0, 1 mM DTT), and lysed by sonication. The supernatant was separated from the cell debris by centrifugation at 15,777 × *g* for 45 min at 4 °C and filtered through a 0.5 µm membrane.

The fractionation of cellular proteins was performed on AKTA Explorer 100 (GE) with anion exchange chromatography HiTrap Q FF and HiTrap Q HP (GE). The prepacked columns were pre-treated according to the instructions. To enrich and pre-fractionate the target proteins, 70 mL of crude CFE (22.4 g L^−1^) were loaded to the HiTrap Q FF and eluted at a flow rate of 1 mL min^−1^ by (A) 25 mM Tris-HCl (pH 8.0) and (B) 25 mM Tris-HCl (pH 8.0), 1 M NaCl with the gradient elution procedures: 0–40 min, 0% B; 40–60 min, 10% B; 60–80 min, 20% B; 80–100 min, 30% B; 100–120 min, 40% B; 120–140 min, 50% B; 140–160 min, 60% B; 160–180 min, 70% B; 180–200 min 100% B. These fractions were collected, concentrated, and analyzed for the oxidoreductase activities. Since fractions eluted by 10–40% B showing enzymatic activity, they were pooled and desalted by 10 kDa concentrator (Millipore Amicon Ultra). For fine fractionation, the pooled fractions were subjected to HiTrap Q HP and separated into 34 fractions with a linear gradient elution by increasing the concentration of B from 10 to 40% in 120 min. The enzyme activities of these fractions were analyzed in the presence of NADPH or BCF.

The fractions with highest activity and without detectable activity were analyzed by Thermo Scientific™ Q Exactive™ hybrid quadrupole-Orbitrap mass spectrometer. Among the identified proteins, the annotated oxidoreductases with higher reliability and abundance (Unique PepCount ≥4) were chosen and analyzed. The candidates were heterologously expressed in *E. coli* and purified, and their enzymatic activities were monitored as described above.

### Transcriptome sequencing and quantitative real-time PCR analysis

For transcriptome sequencing of *Actinoplanes* sp. SE50/110, the cells were harvested from 1-mL fermentation culture at 24, 48, or 72 h by centrifuging at 4 °C, freezed in liquid nitrogen for 0.5–1 h, and stored at −80 °C. The RNA extraction, transcriptome sequencing, and data analysis were performed by Shanghai Biotechnology Corporation. The expression of each gene was calculated as fragments per kilobase of exon per megabase of library size.

Because the productivity of **1** and transcription of genes in *acb* cluster were highest at 48 h over the fermentation course of *Actinoplanes* sp. SE50/110 (Supplementary Fig. 55), we took a sample at 48 h to verify the transcription of related genes. The mycelia were harvested from 1-mL fermentation culture by centrifugation at 15,777 × *g* for 10 min at 4 °C, stored in liquid nitrogen for 0.5–1 h, re-suspended with 1 mL Redzol, and broken by high-speed homogenizer (65 Hz, 30 s, repeated three times) with glass beads. Then, total RNA of the samples was extracted according to the instruction given in the kit (SBS Genetech). The quality and concentration of total RNA were analyzed by NanoDrop 2000 spectrophotometer. The contaminated DNA was digested by DNase I (Thermo Fisher) according to the instructions. Then, the digested sample was used as a template for PCR amplification using *hrdB*-q-F/*hrdB*-q-R as primers (Supplementary Data 1) to verify whether DNA was completely removed. The reverse transcription was performed using RevertAid^TM^ H Minus First Strand cDNA Synthesis Kit (Thermo Fisher). The transcription of the related genes was monitored by quantitative real-time PCR using Maxima^TM^ SYBR Green/ROX qPCR Maxter Mix (Thermo Fisher)^58^. The relative transcription values of the target genes were calculated by the 2^-∆∆Ct^ method^59^ with the housekeeping gene *hrdB* (*ACPL_1268*) as an internal control.

### Analysis of enzyme activities in crude CFE

From 2-mL 2-day fermentation broth, mycelia of *Actinoplanes* sp. SE50/110 or its derivatives were harvested by centrifuged at 4 °C, washed twice, and re-suspended in 0.5 volume pre-cooling buffer (25 mM Tris-HCl, pH 8.0, 300 mM NaCl, 1 mM DTT). After lysis by sonication, the supernatant was obtained by centrifugation at 15,777 × *g* for 20 min at 4 °C. Concentrations of the total proteins in the extracts were determined with the Bradford Protein Assay Kit.

For the phosphatase activity analysis, 7 µL of crude enzyme solution was added to the mixture (20 µL) containing 25 mM Tris-HCl (pH 7.5), 10 mM MgCl_2_, 1.25 mM **10** or **11**, and 1 mM DTT, and then incubated at 30 °C for 4 h. The reaction was stopped by adding two volumes of methanol and analyzed by GC-QMS.

For the analysis of G-1-PT activity, 2 µL of crude enzyme solution was added to the mixture (20 µL) containing 50 mM Tris-HCl (pH 7.5), 12 mM MgCl_2_, 24 mM **5**, 6 mM dTTP, 0.12 U pyrophosphatase, and 1 mM DTT, and then incubated at 30 °C for 15 min^60^. The production of dTDP-D-glucose was analyzed by HPLC-QQQ/MS. One enzyme unit (U) is defined as the amount of dTDP-D-glucose in nanomole produced in 1 min.

For the analysis of 4,6-DH activity, 2 µL of crude enzyme solution was added to the mixture (20 µL) containing 50 mM Tris-HCl (pH 7.5), 0.4 mM dTDP-D-glucose, 1 mM NADP^+^, and 1 mM DTT, and then incubated at 30 °C for 45 min^61^. The production of dTDP-4-keto-6-deoxy-D-glucose was analyzed by HPLC-QQQ/MS. One enzyme unit (U) is defined as the amount of dTDP-4-keto-6-deoxy-D-glucose in nanomole produced in 1 min. The average specific activity in SE50/110::pSET152 was calculated and set to 1 as standard. The specific activities in related mutants were accordingly calculated.

### Analysis of the concentrations of endogenous **10** and **11**

After 2-day fermentation, two samples were, respectively, obtained from 1 mL of fermentation culture by centrifugation. One sample was washed with 0.25 M hydrochloric acid and dried for biomass measurement, and the other sample was rapidly washed twice with cold 0.9% NaCl and put in liquid nitrogen for 0.5–1 h. Then, the sample was re-suspended in equal volume of water, lysed by sonication, and rapidly mixed with 1-mL cold methanol. The supernatant was separated from the cell debris by centrifugation at 15,777 × *g* for 20 min at 4 °C and freeze-dried. The powder was re-suspended by 100 µL methanol/water solution (50:50, v/v), centrifuged at 15,777 × *g* for 20 min and subjected to HPLC-QQQ/MS analysis. The concentrations of **10** and **11** were calculated according to the biomass. The concentrations of **10** or **11** in SE50/110::pSET152 or *Actinoplanes* sp. SE50/110 were set to 1 as standards, and those of related mutants were accordingly calculated.

### Reporting summary

Further information on research design is available in the Nature Research Reporting Summary linked to this article.

## Supplementary information

Supplementary Information

Reporting Summary

Description of Additional Supplementary Files

Supplementary Dataset 1

Supplementary Dataset 2

Supplementary Dataset 3

Supplementary Dataset 4

## Data Availability

Data supporting the findings of this work are available within the paper and its Supplementary Information files. A reporting summary for this article is available as a Supplementary Information file. The datasets generated and analyzed during the current study are available from the corresponding author upon request. The source data underlying Figs. 2c, 3–5, 6b, c, e, f, and 7 are provided as a Source Data file. The sequences for all genes described in this manuscript are available in the GenBank databases under the accession numbers Y18523.4, CP003170.1, CP003275.1, CP000480.1, CP002496.1, and AP009048.1.

## Source Data

Source Data
