# Supplementary material for: A severe leakage of intermediates to shunt products in acarbose biosynthesis
Source: Nat Commun. 2020 Mar 19;11:1468. doi: 10.1038/s41467-020-15234-8 (PMC7081202; doi:10.1038/s41467-020-15234-8)
Supplement: Supplementary file 1 — Supplementary Information [file 41467_2020_15234_MOESM1_ESM.pdf]

A severe leakage of intermediates to shunt products in acarbose biosynthesis

Zhao *et al.*

Supplementary Method 1. Genetic manipulation of *Actinoplanes* sp. SE50/110 and corresponding derivatives[6].

Intergeneric conjugation between *E. coli* and *Actinoplanes* sp. SE50/110

A culture of the ET12567(pUZ8002) containing recombinant plasmid was grown overnight in LB with 50 mg L^{-1} apramycin, 25 mg L^{-1} chloramphenicol and 50 mg L^{-1} kanamycin, and then inoculated (1/20, *v/v*) to fresh LB broth and grown to OD_{600} of 0.8-1.0. Cells were washed for three times with an equal volume of LB, re-suspended in 0.5 volume of LB (about 10^7-10^8 CFU) and then diluted for 100 folds. *Actinoplanes* sp. SE50/110 was cultivated on STY plate for 2-3 days, inoculated to SM medium for a 36-h growth and subsequently transferred (1/10, *v/v*) to TSB medium for a further 8 to 12-h incubation. The mycelia were washed twice, re-suspended in equal volume of LB (about 10^6-10^7 CFU) and then diluted for 100 folds.

0.5-mL of mycelial dilution and 0.5-mL of *E. coli* dilution were mixed and spread on SFM agar plate (mannitol 2%, full-fat soya flour 2%, agar 2%) containing 10 mM $MgCl_2$. Then, SFM agar plates were incubated for 32 h at 30 °C and overlaid with 1 mL sterile water containing 1 mg apramycin and 0.5 mg trimethoprim. These plates were incubated at 30 °C for another 5-7 days.

Gene manipulations by homologous recombination

For the construction of *acb*-deleted mutant, the exconjugants were streaked to STY plates with 50 mg L^{-1} apramycin and 25 mg L^{-1} trimethoprim for 2-3 days. In order to promote the DNA recombination in the exconjugants, the mycelia from STY plate were inoculated to SM broth. Then a 36-h culture was transferred (1/10, *v/v*) to fresh SM broth for another 36-48-h cultivation. The mycelia were diluted for 10 folds and filtered with non-absorbent cotton

wool. The filtrate was diluted for 10^4-10^5 folds and cultivated for 4-5 days on STY plates with 50 mg L^{-1} 5-FC. The apramycin-sensitive colonies were selected by replica plating and verified by PCR using primers *acb*-V-F/*acb*-V-R. The mutants with double-crossover recombination (QQ-3) gave a 1.5-kb amplified product, whereas *Actinoplanes* sp. SE50/110 gave no amplified product (Supplementary Fig. 49a). Meanwhile, the corresponding PCR products were further verified by DNA sequencing.

The same method was used for construction of other gene-deleted mutants. The schematic representation of gene deletions and insertions in the chromosome and the verification of the mutants by PCR were shown in Supplementary Fig. 49-54.

Overexpression or complementation of genes using ØC31-derived integrative plasmid

For complementation of *acbJ* gene in QQ-5, the exconjugants were streaked to STY plates with 50 mg L^{-1} apramycin and 25 mg L^{-1} trimethoprim, cultivated for 2-3 days, and then transferred to SM liquid medium with 50 mg L^{-1} apramycin and cultivated for 36 h to isolate total DNA. The mutants were confirmed by PCR amplification using primers *kasOp*-Xba*I-F/*kasOp*-acbJ*-R. The PCR product was further verified by DNA sequencing. The similar method was used for the expression of other genes in corresponding *Actinoplanes* strains.

a

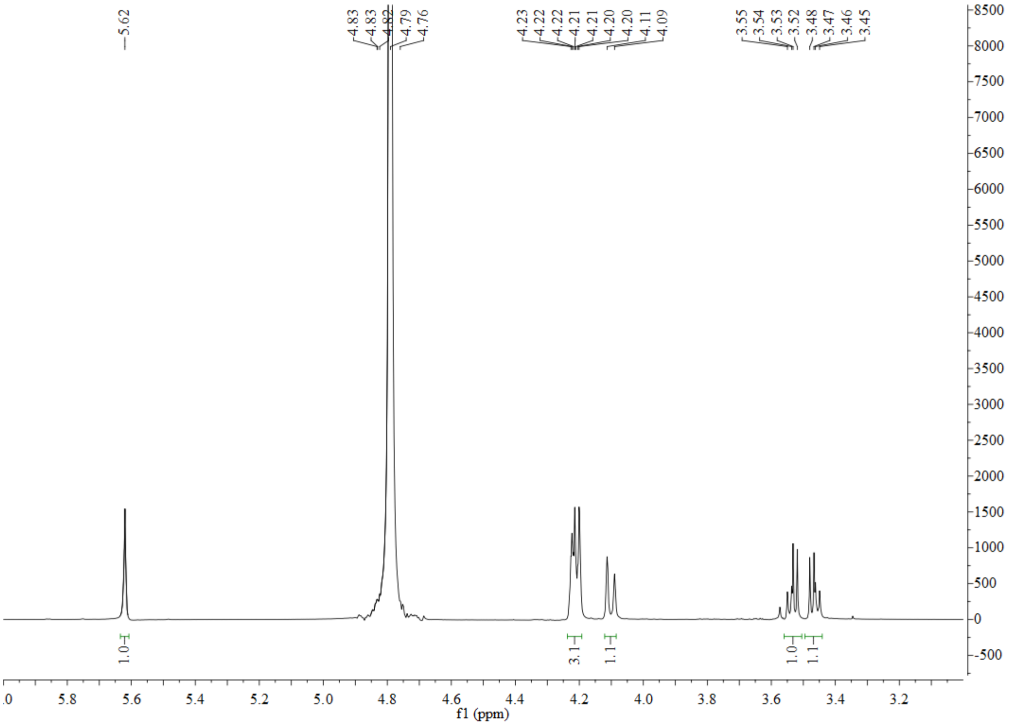

b

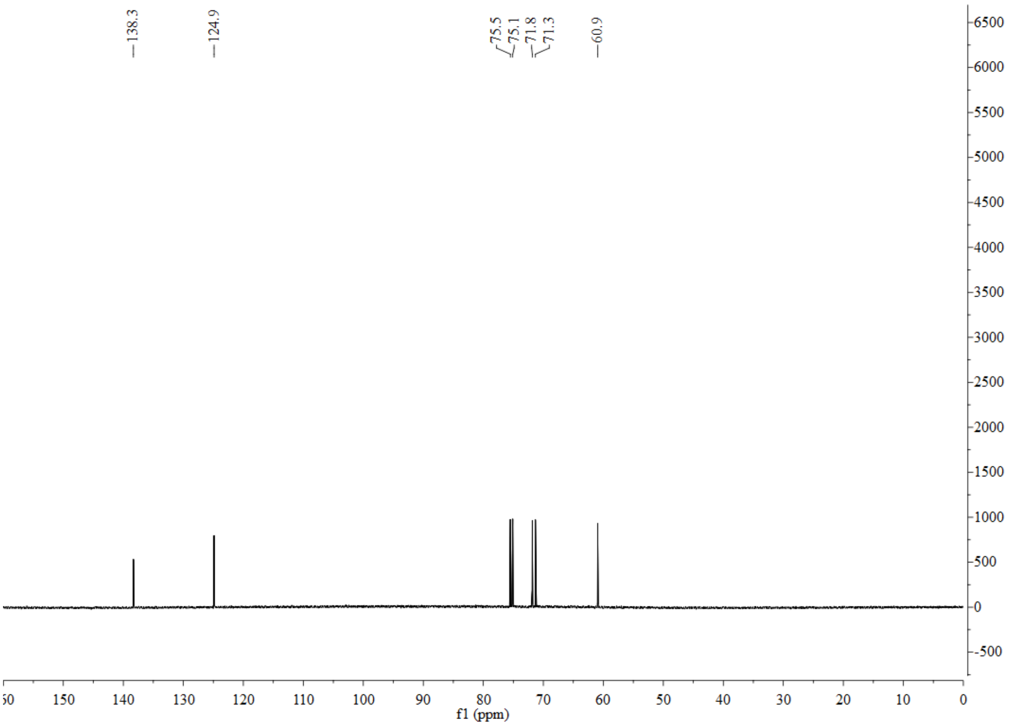

Supplementary Figure 1. ^{1}H and ^{13}C NMR spectra of 8. a ^{1}H NMR spectrum. **b** ^{13}C NMR spectrum. The NMR spectra were collected in D_2O at 600 (^{1}H NMR) and 150 MHz (^{13}C NMR) on Bruker Avance III 600 spectrometer (14.09 T).

a

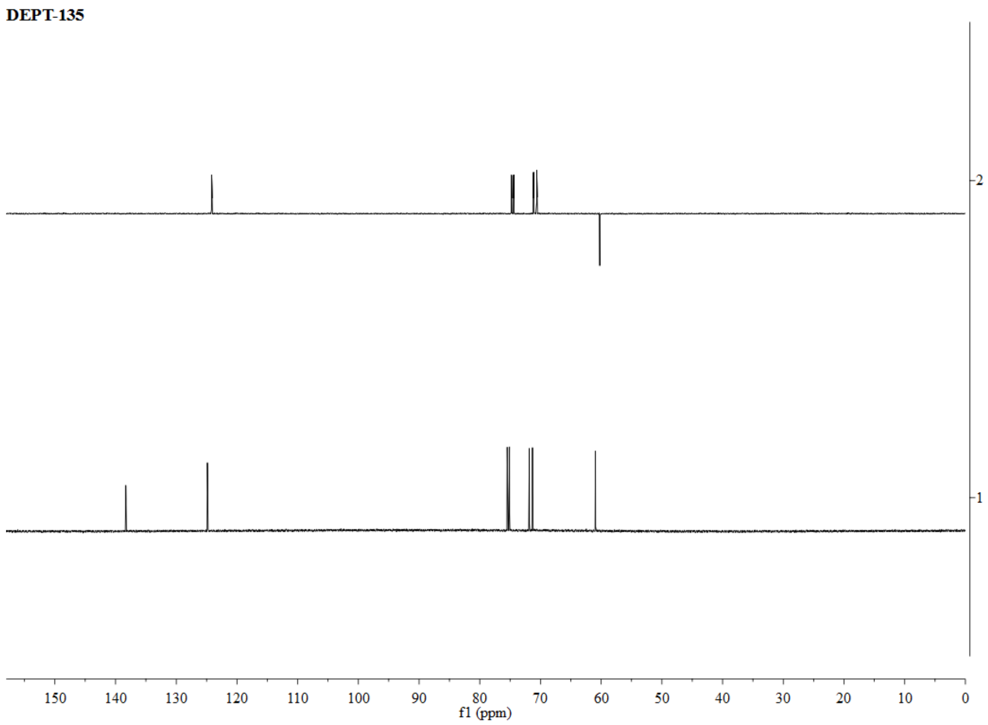

b

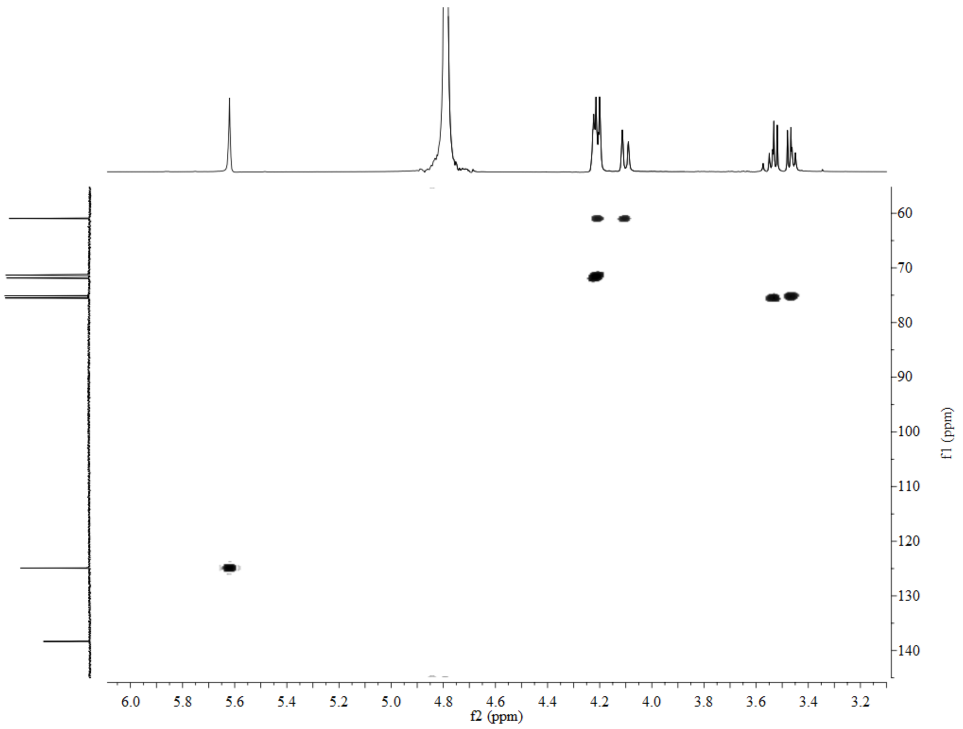

Supplementary Figure 2. DEPT and HSQC spectra of 8. a DEPT spectrum. **b** HSQC spectrum. The NMR spectra were collected in D_2O at 600 (^{1}H NMR) and 150 MHz (^{13}C NMR) on Bruker Avance III 600 spectrometer (14.09 T).

a

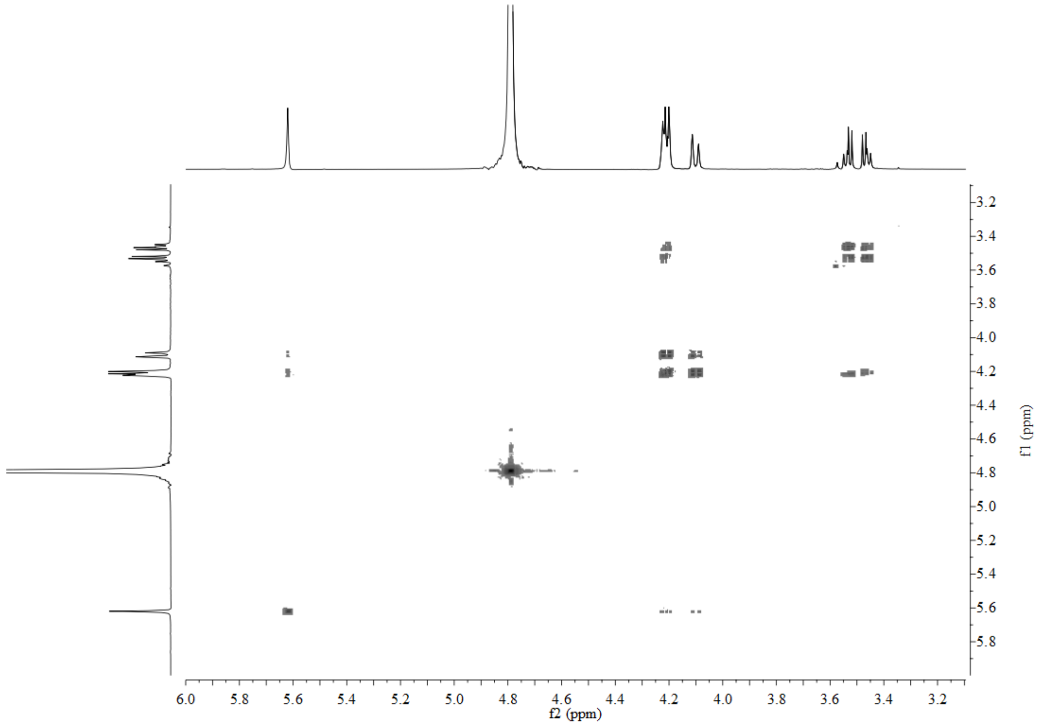

b

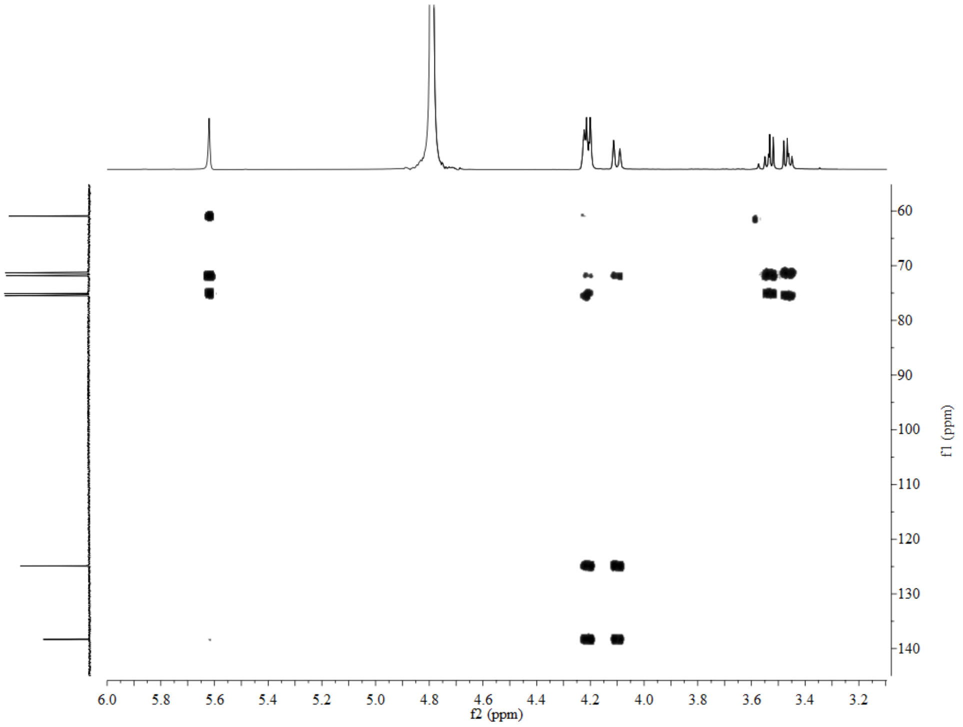

Supplementary Figure 3. ^1H-^1H COSY and HMBC spectra of 8. a ^1H-^1H COSY spectrum. **b** HMBC spectrum. The NMR spectra were collected in D_2O at 600 (^1H NMR) and 150 MHz (^{13}C NMR) on Bruker Avance III 600 spectrometer (14.09 T).

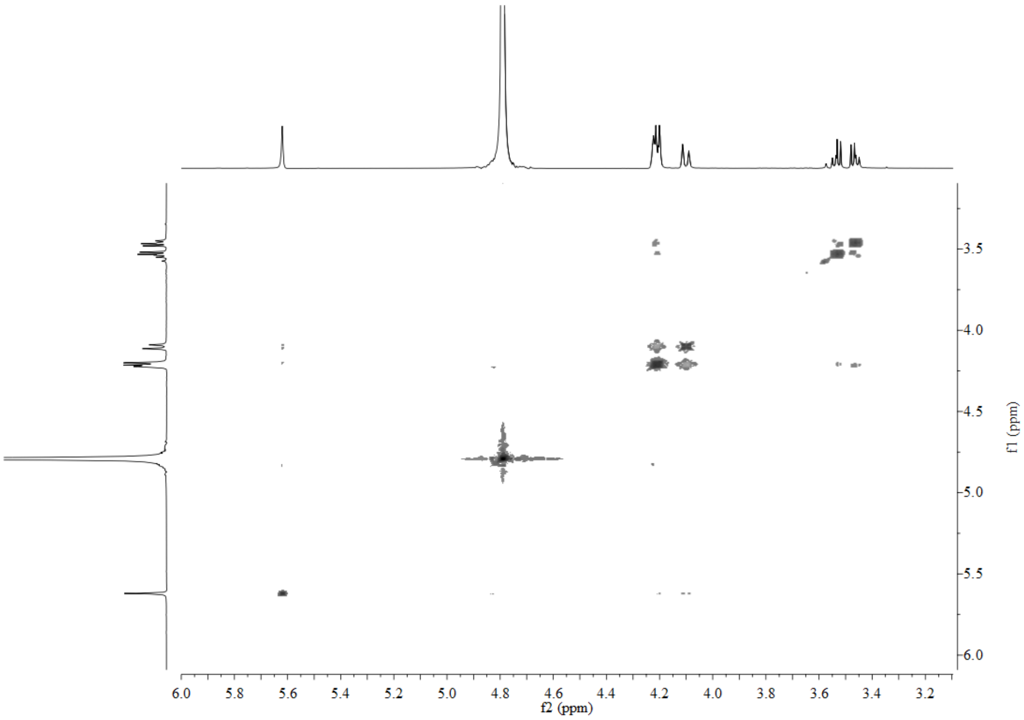

Supplementary Figure 4. NOESY spectrum of 8. The NMR spectrum was collected in D_2O at 600 (^1H NMR) and 150 MHz (^{13}C NMR) on Bruker Avance III 600 spectrometer (14.09 T).

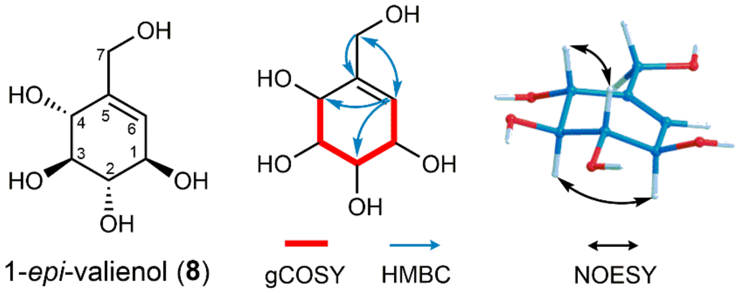

Supplementary Figure 5. Key 2D NMR correlations for 8. For more details, see also Supplementary Fig. 1-4.

a

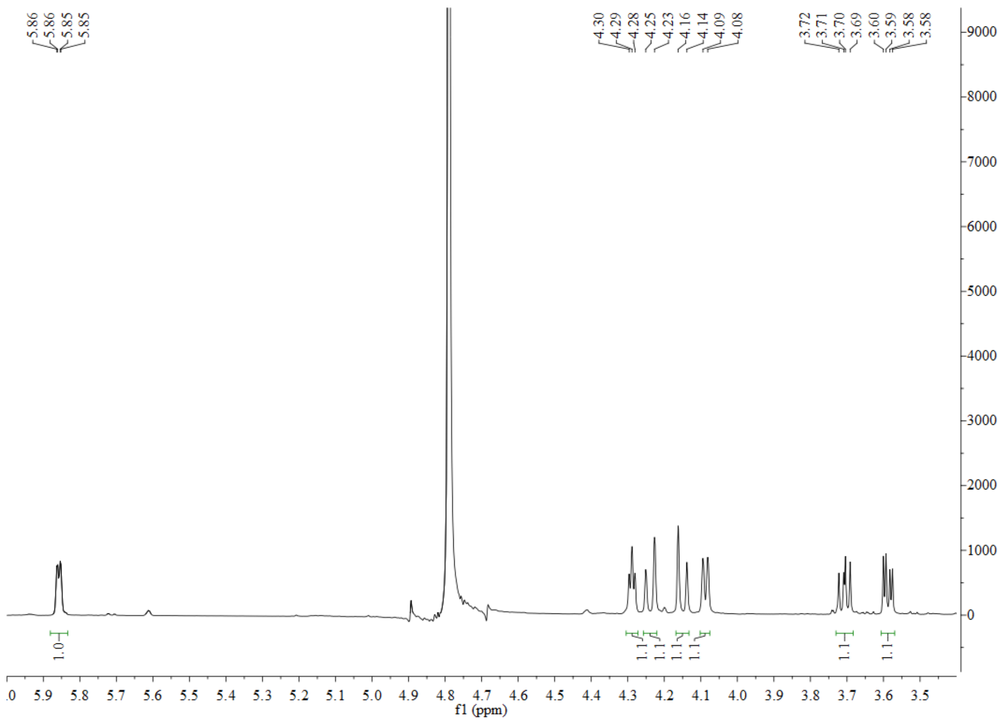

b

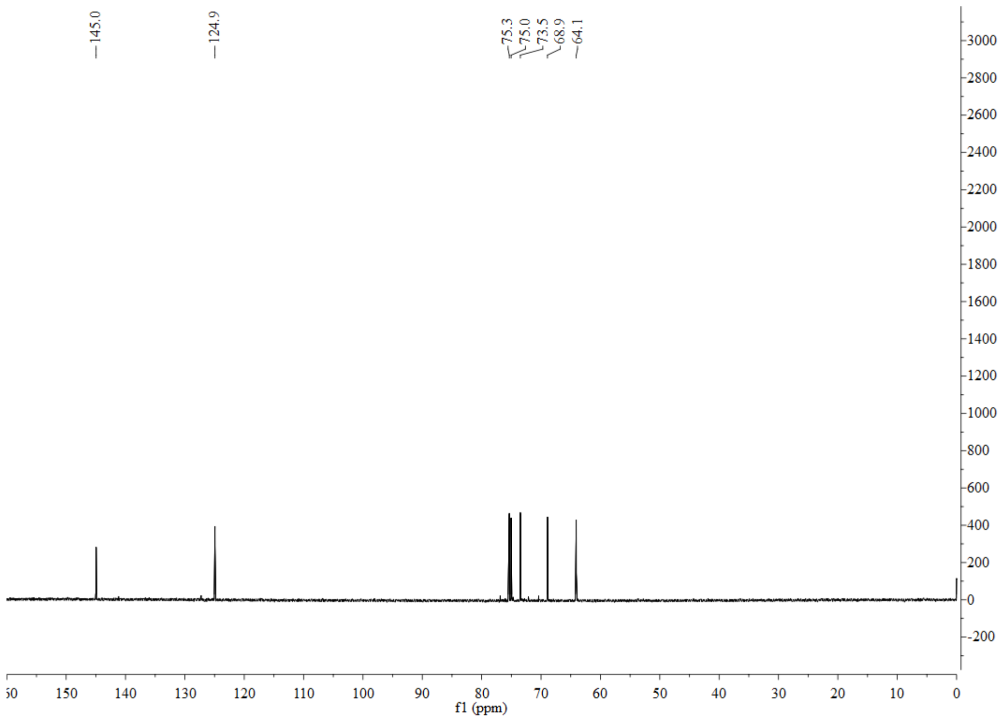

Supplementary Figure 6. ^{1}H and ^{13}C NMR spectra of 9. a ^{1}H NMR spectrum. **b** ^{13}C NMR spectrum. The NMR spectra were collected in D_2O at 600 (^{1}H NMR) and 150 MHz (^{13}C NMR) on Bruker Avance III 600 spectrometer (14.09 T).

a

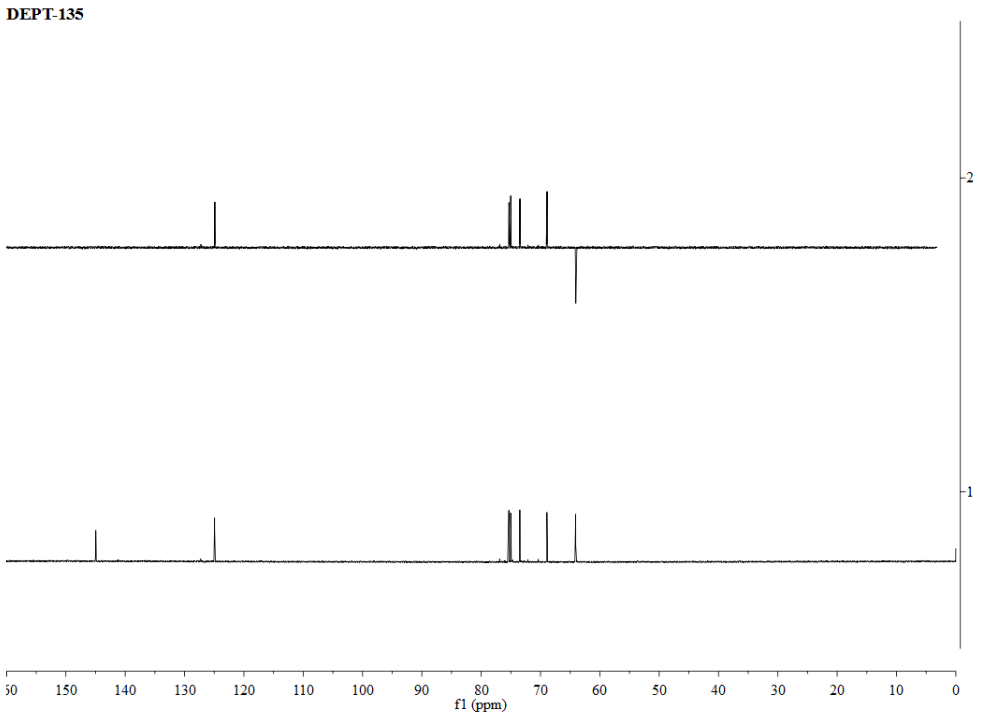

b

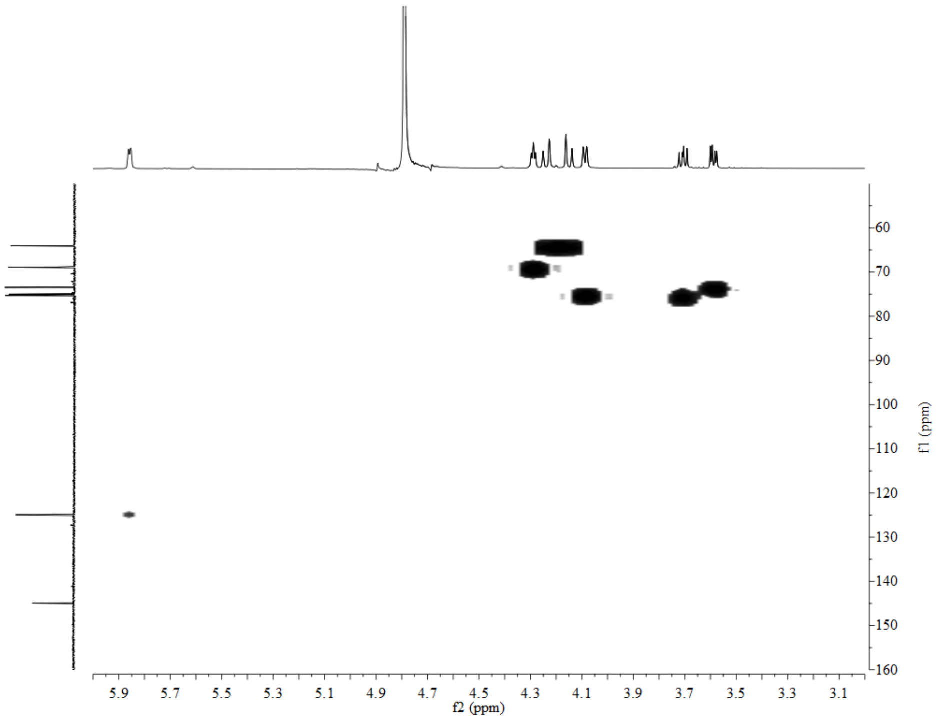

Supplementary Figure 7. DEPT and HSQC spectra of 9. a DEPT spectrum. **b** HSQC spectrum. The NMR spectra were collected in D_2O at 600 (^1H NMR) and 150 MHz (^{13}C NMR) on Bruker Avance III 600 spectrometer (14.09 T).

a

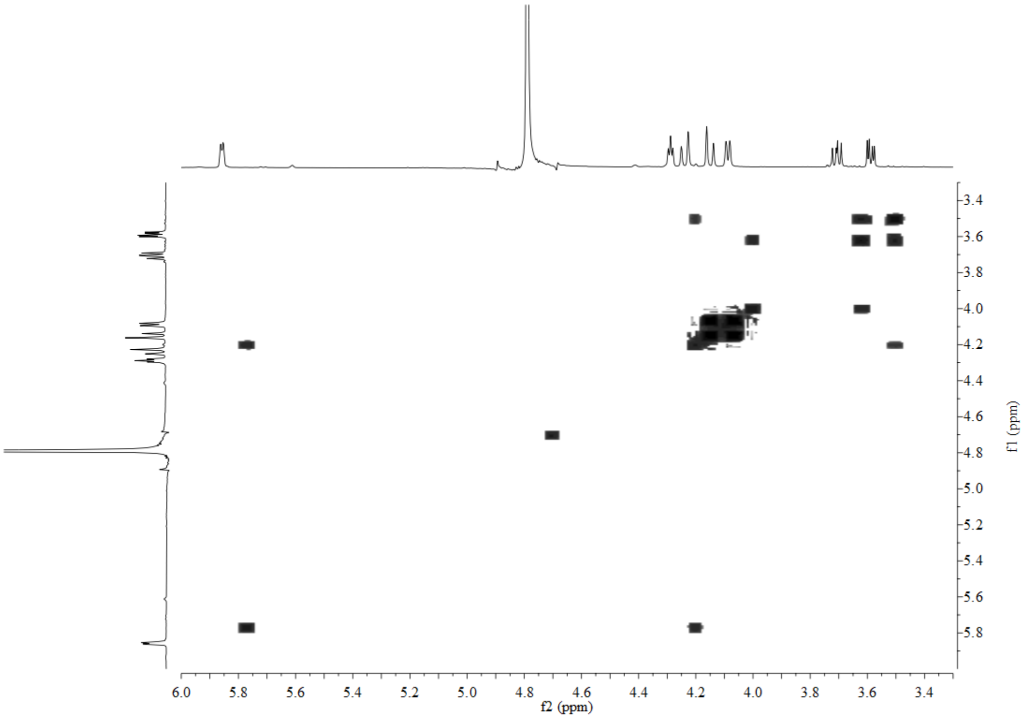

b

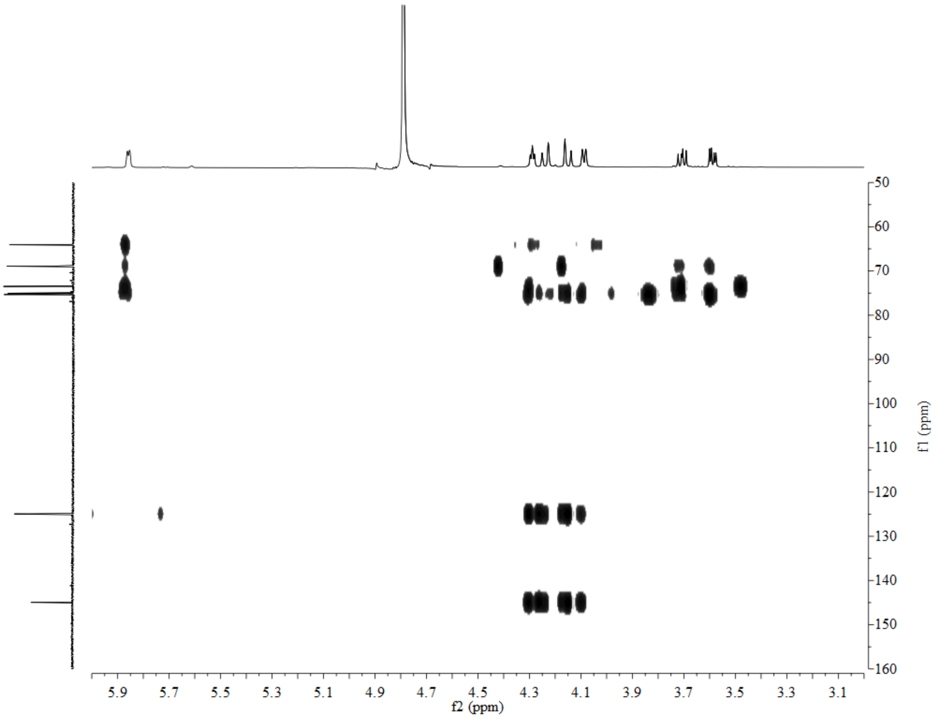

Supplementary Figure 8. ^{1}H-^{1}H COSY and HMBC spectra of 9. a ^{1}H-^{1}H COSY spectrum. **b** HMBC spectrum. The NMR spectra were collected in D_2O at 600 (^{1}H NMR) and 150 MHz (^{13}C NMR) on Bruker Avance III 600 spectrometer (14.09 T).

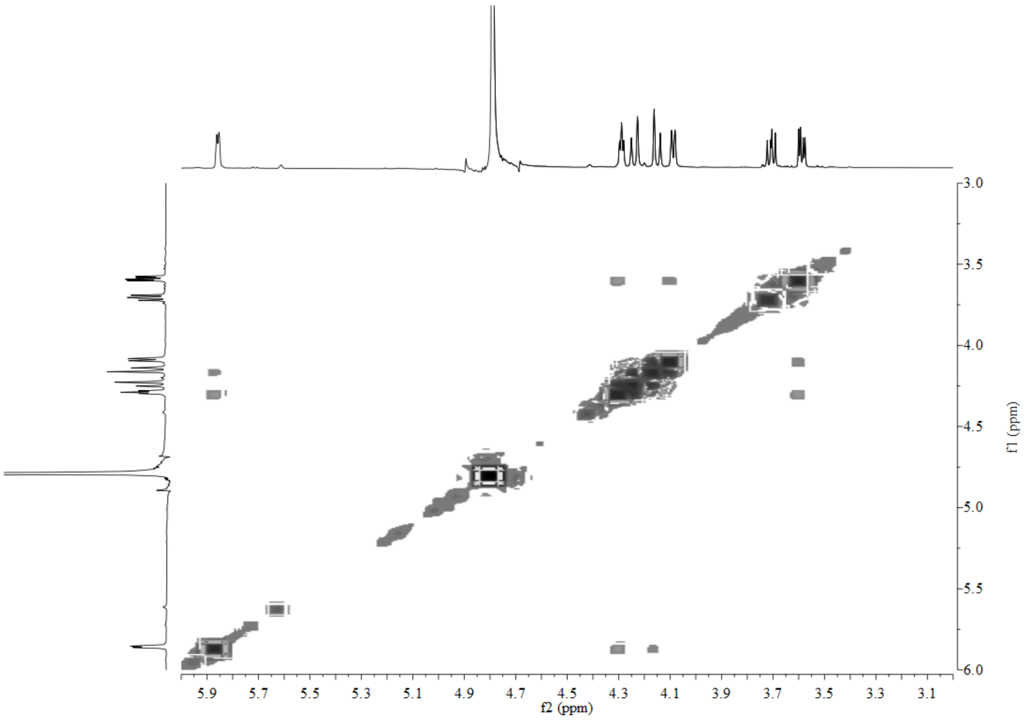

Supplementary Figure 9. NOESY spectrum of 9. The NMR spectrum was collected in D_2O at 600 (^1H NMR) and 150 MHz (^{13}C NMR) on Bruker Avance III 600 spectrometer (14.09 T).

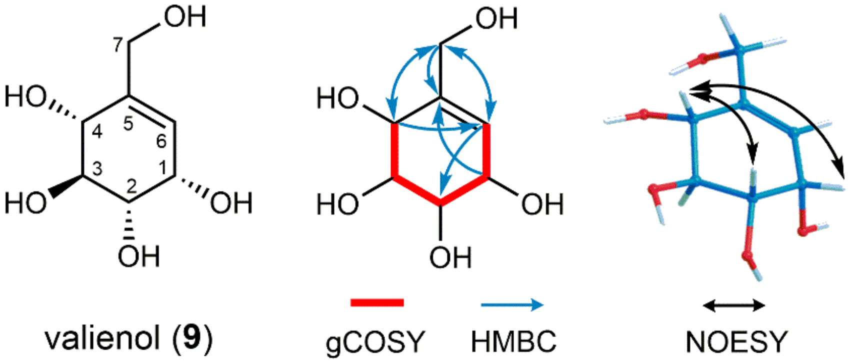

Supplementary Figure 10. Key 2D NMR correlations for 9. For more details, see also Supplementary Fig. 6-9.

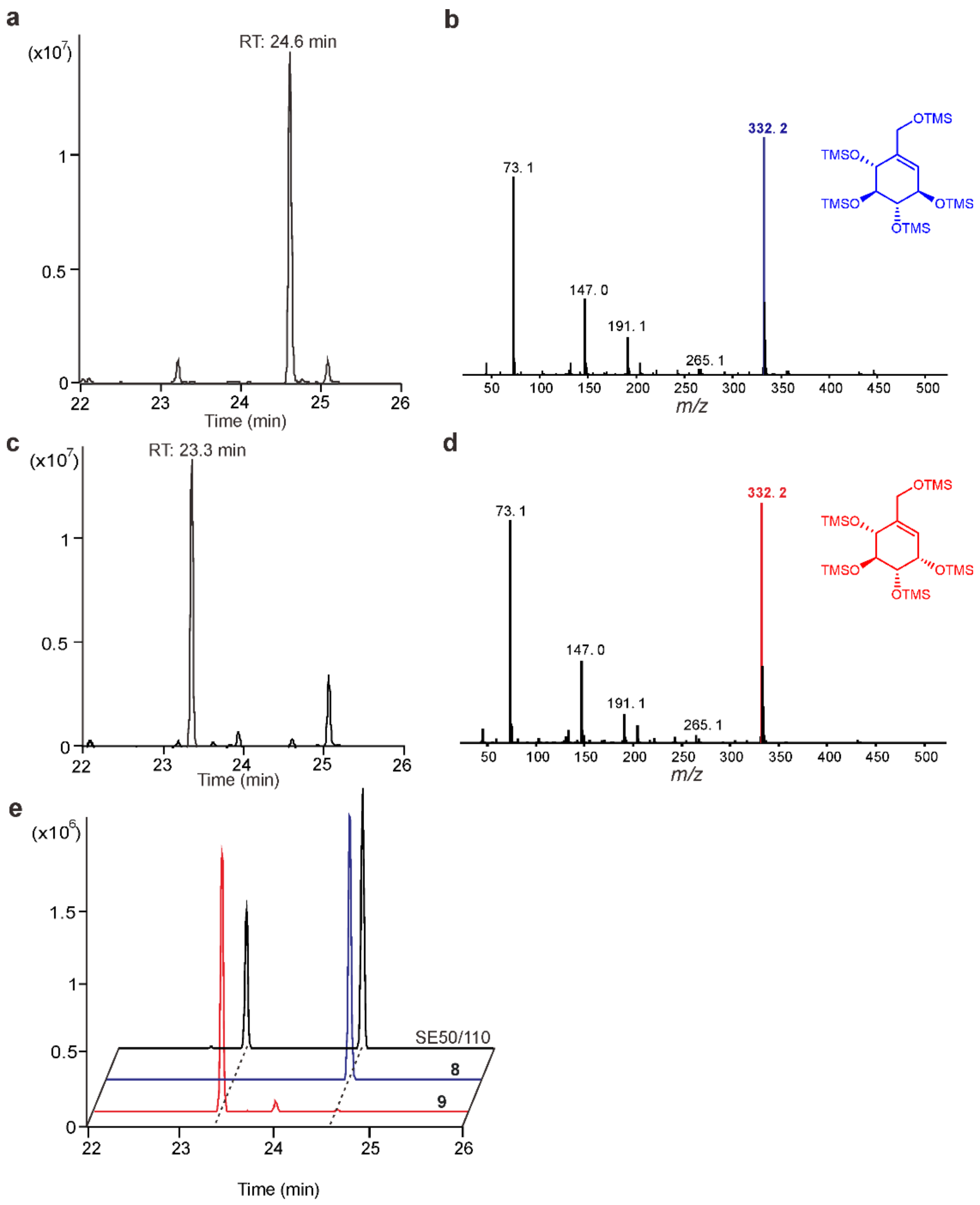

Supplementary Figure 11. GC-QMS analysis of 8 and 9 after derivatization by BSTFA. **a** The total ion chromatogram of TMS-1-*epi*-valienol. The retention time (RT) is 24.6 min. **b** The mass spectrum and structure of TMS-1-*epi*-valienol. The unique product ion *m/z*=332.2 with highest abundance is chosen for quantitative and qualitative analysis. **c** The total ion chromatogram of TMS-valienol. The RT is 23.3 min. **d** The mass spectrum and structure of TMS-valienol. **e** GC-QMS analysis of purified **8** and **9**, and their production in the fermentation broth of *Actinoplanes* sp. SE50/110 after

derivatization by BSTFA. The chromatograms show the extraction of unique product ion *m/z*=332.2. TMS is the abbreviation of trimethyl silicyl. Although TMS-1-*epi*-valienol and TMS-valienol have same mass spectra, their retention times are clearly different when analyzed by GC-QMS, which helps us to quantitatively and qualitatively analyze **8** and **9** according to the requirements in following works.

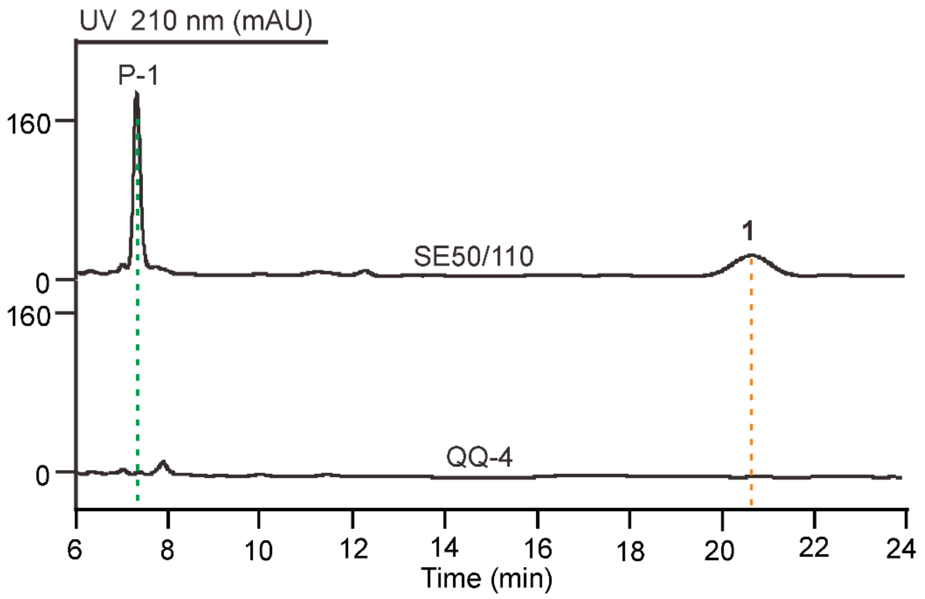

Supplementary Figure 12. Inactivation of *acbC* in *Actinoplanes* sp. SE50/110. HPLC profiles of parent strain *Actinoplanes* sp. SE50/110 and Δ*acbC* mutant QQ-4.

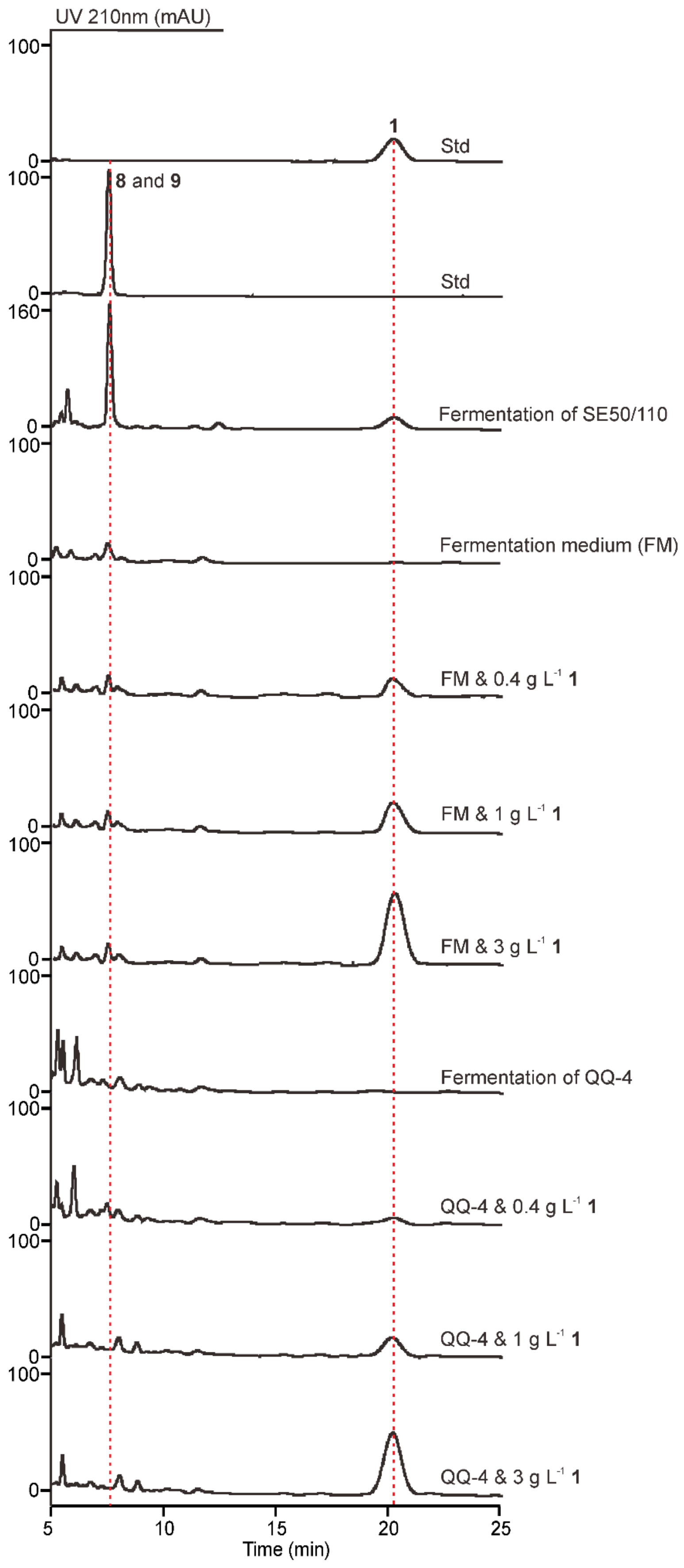
UV 210nm (mAU)
1
Std
8 and 9
Std
Fermentation of SE50/110
Fermentation medium (FM)
FM & 0.4 g L-1 1
FM & 1 g L-1 1
FM & 3 g L-1 1
Fermentation of QQ-4
QQ-4 & 0.4 g L-1 1
QQ-4 & 1 g L-1 1
QQ-4 & 3 g L-1 1
Time (min)

Supplementary Figure 13. HPLC analysis of the products after feeding of 1 to *acb*C-deleted mutant QQ-4. The HPLC profiles show the analysis of the standard (std) of **1**, the mixture of **8** and **9** with a molar ratio of 1:1 (as standard), the fermentation broth of *Actinoplanes* sp. SE50/110, the fermentation medium without inoculation (FM), FM with 0.4 g L^{-1} **1**, FM with 1 g L^{-1} **1**, FM with 3 g L^{-1} **1**, the fermentation broth of QQ-4, and the fermentation broth of QQ-4 fed with 0.4 g L^{-1}, 1 g L^{-1} or 3 g L^{-1} **1**. These results indicate that large amounts of **8** and **9**, accumulated in the fermentation broth of *Actinoplanes* sp. SE50/110, are not derived from the hydrolysis of **1** via enzymatic or non-enzymatic reactions.

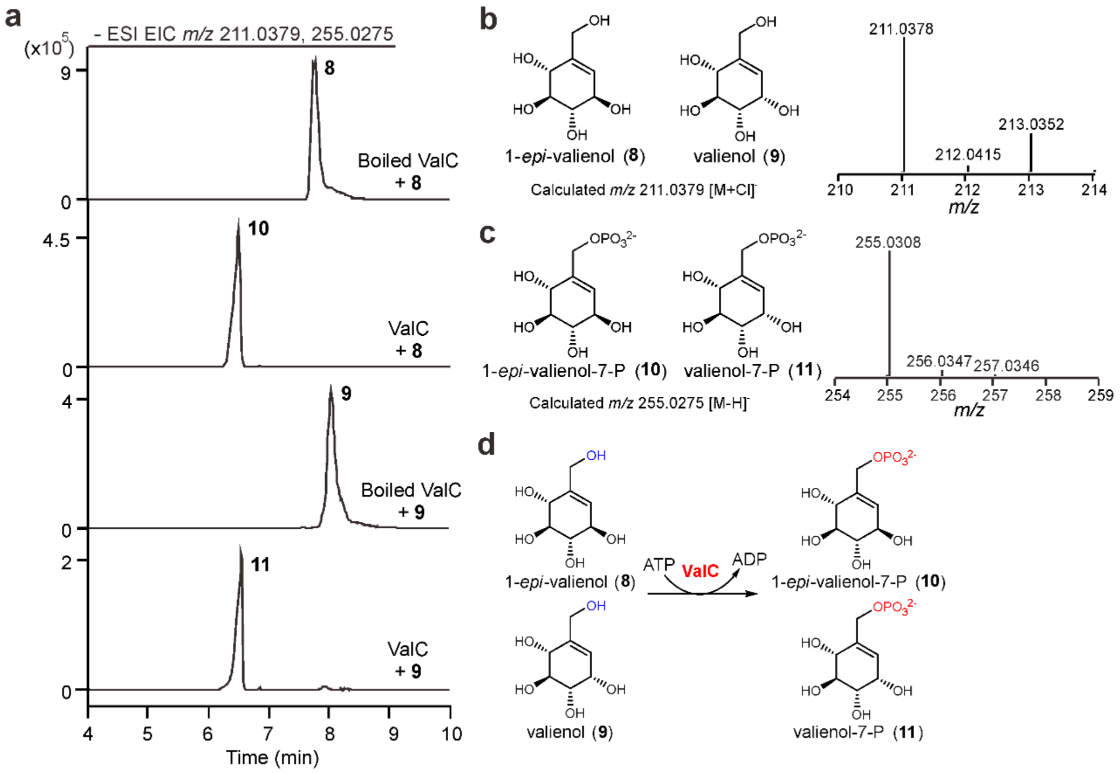

Supplementary Figure 14. ValC catalyzes the phosphorylation of 8 to 10 and 9 to 11. a HPLC-TOF/MS analysis of the reaction products of boiled ValC with **8**, ValC with **8**, boiled ValC with **9** and ValC with **9**. All of the chromatograms show the simultaneous extraction of calculated ions *m/z*=211.0379 $[M+Cl]^-$ for **8** and **9** and *m/z*=255.0275 $[M-H]^-$ for **10** and **11**. **b** The high resolution spectra of **8** and **9** analyzed by HPLC-TOF/MS. **c** The high resolution spectra of **10** and **11** analyzed by HPLC-TOF/MS. **d** Schematic illustration of the ValC-catalyzed phosphorylation of **8** to **10** and **9** to **11**.

a

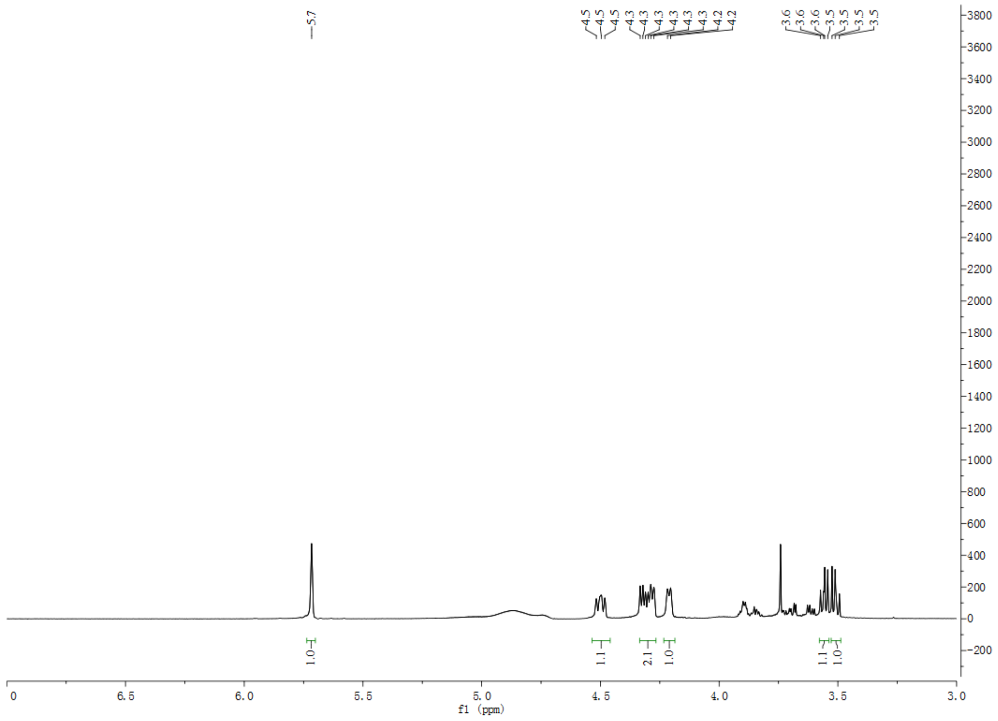

b

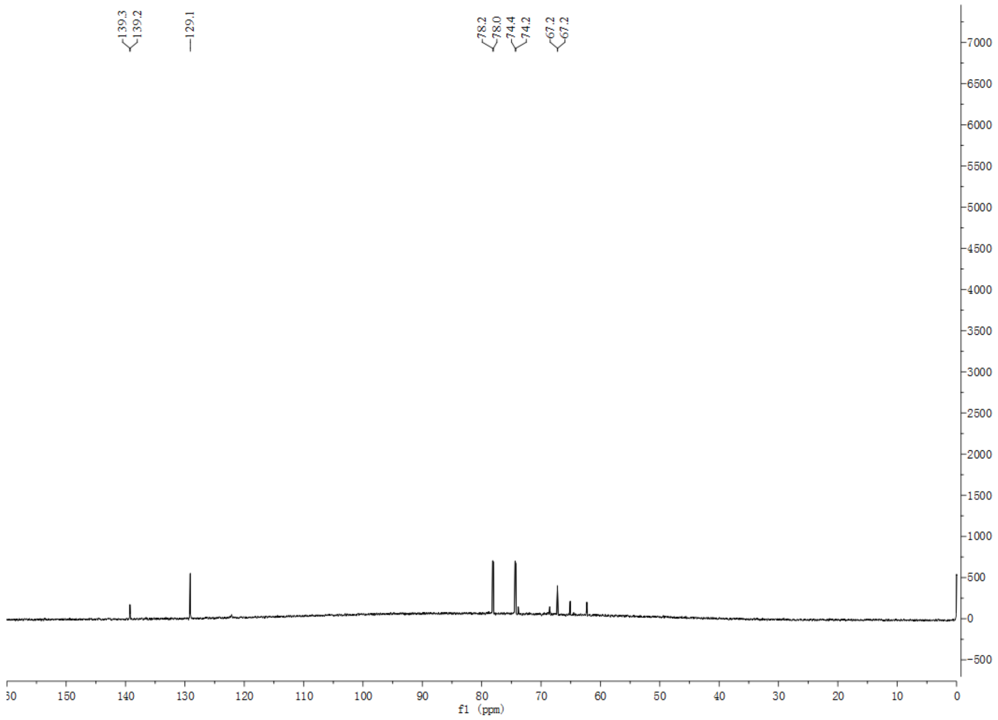

Supplementary Figure 15. ^{1}H and ^{13}C NMR spectra of 10. a ^{1}H NMR spectrum. **b** ^{13}C NMR spectrum. The NMR spectra were collected in D_2O at 600 (^{1}H NMR) and 150 MHz (^{13}C NMR) on Bruker Avance III 600 spectrometer (14.09 T).

a

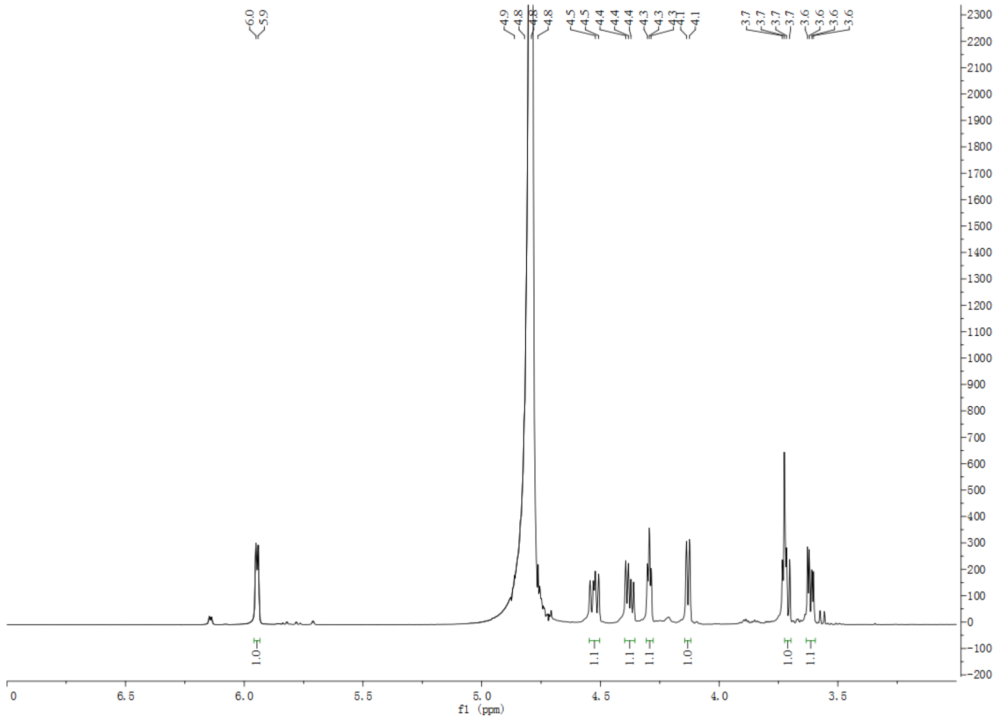

b

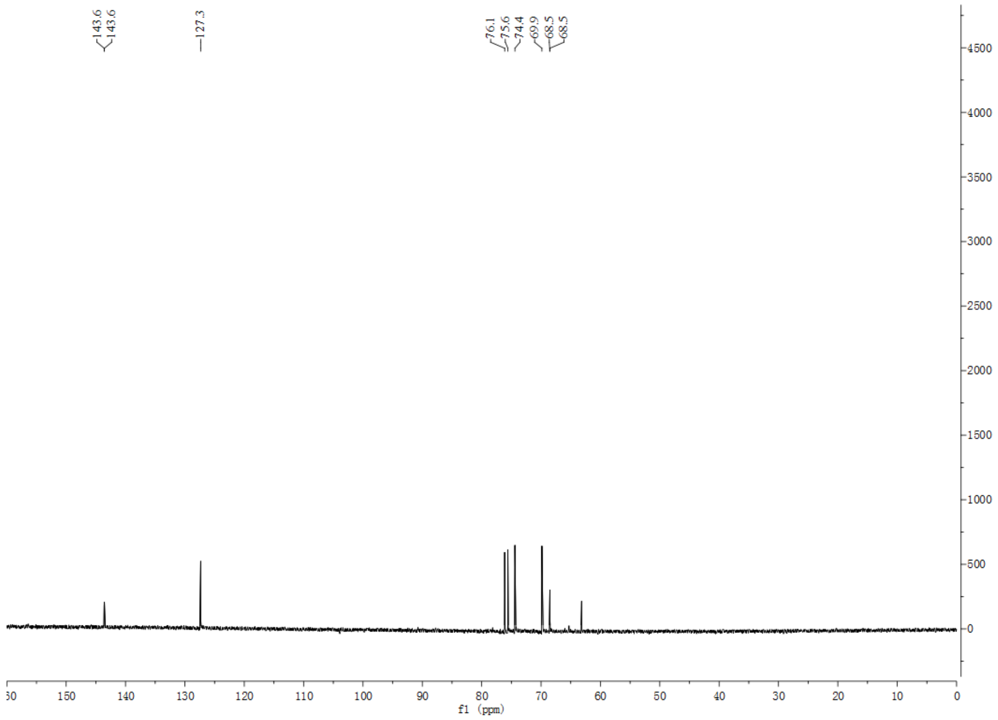

Supplementary Figure 16. ^{1}H and ^{13}C NMR spectra of 11. a ^{1}H NMR spectrum. **b** ^{13}C NMR spectrum. The NMR spectra were collected in D_2O at 600 (^{1}H NMR) and 150 MHz (^{13}C NMR) on Bruker Avance III 600 spectrometer (14.09 T).

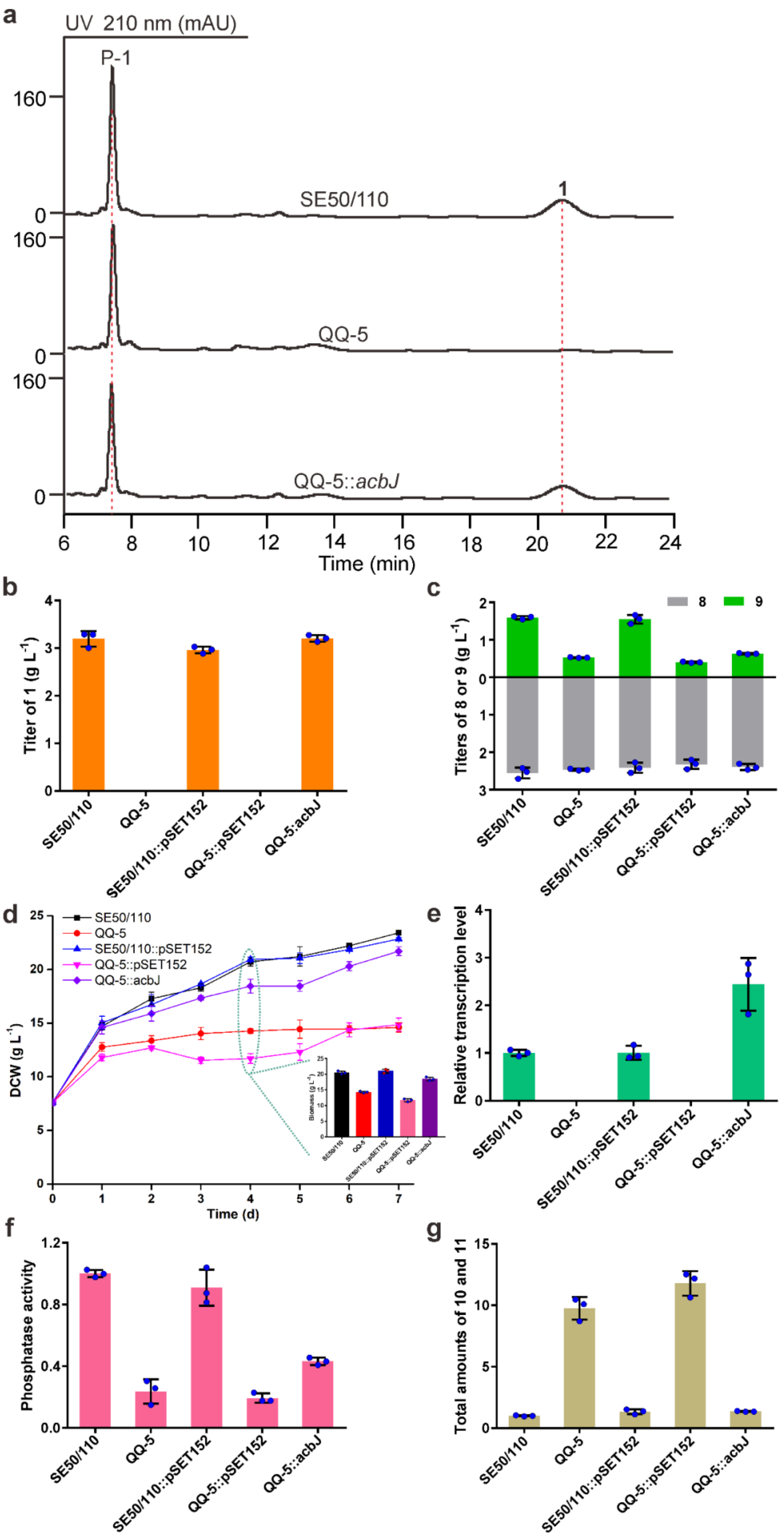

a
UV 210 nm (mAU)
P-1
160
0
SE50/110
1
QQ-5
QQ-5::acbJ
Time (min)
b
Titer of 1 (g L-1)
SE50/110
QQ-5
SE50/110::pSET152
QQ-5::pSET152
QQ-5:acbJ
c
Titers of 8 or 9 (g L-1)
8
9
QQ-5::acbJ
d
DCW (g L-1)
SE50/110
QQ-5
SE50/110::pSET152
QQ-5::pSET152
QQ-5::acbJ
Time (d)
e
Relative transcription level
f
Phosphatase activity
g
Total amounts of 10 and 11

Supplementary Figure 17. Inactivation and complementation of *acbJ* in mutant QQ-5. a HPLC profiles of parent strain *Actinoplanes* sp. SE50/110, Δ*acbJ* mutant QQ-5 and complemented mutant QQ-5::*acbJ*; **b, c** Analysis of the titers of **1**, **8** and **9** of SE50/110, QQ-5, SE50/110::pSET152 (as control), QQ-5::pSET152 (as control) or QQ-5::*acbJ* after 4-day fermentation. **d** The time courses of growth (including the biomass of day 4) of the involved strains. **e-g** Analysis of the transcription of *acbJ*, the phosphatase activity and the total amounts of **10** and **11** of the involved strains after 2-day fermentation. For complementation, *acbJ* was cloned under the control of *kasOp** promoter**.** *Actinoplanes* sp. SE50/110 and dry cell weight are abbrevieted as SE50/110 and DCW, respectively. Error bars, mean ± SD (n=3 biological replicates).

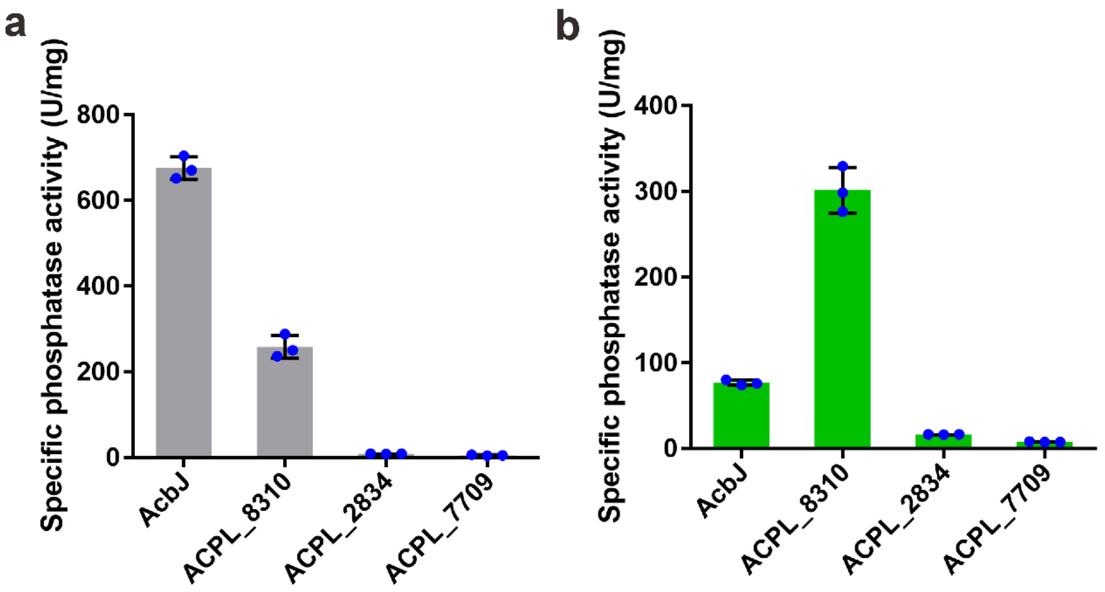

Supplementary Figure 18. Comparison of the catalytic activities of the phosphatases. Specific phosphatase activities of the involved proteins on **10** (**a**) or **11** (**b**). Error bars, mean ± SD (n=3 biological replicates).

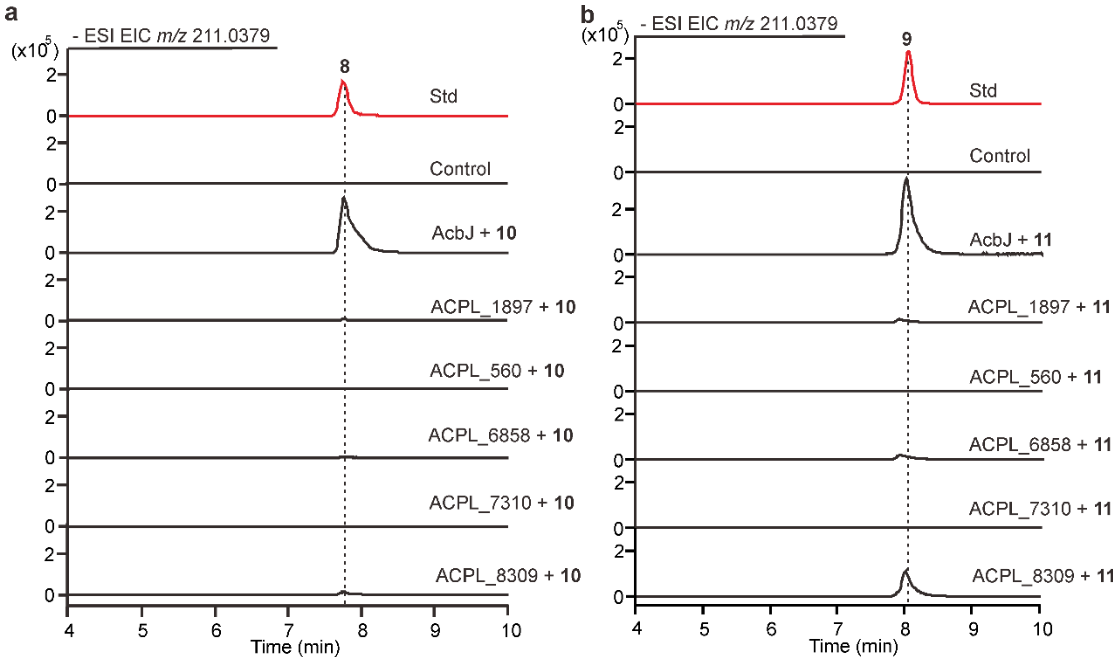

Supplementary Figure 19. Identification of hydrolases involved in the dephosphorylation of 10 and 11. a, b HPLC-TOF/MS analysis of the dephosphorylated products of **10** and **11** catalyzed by AcbJ (as positive control), ACPL_1897, ACPL_560, ACPL_6858, ACPL_7310 or ACPL_8309, with the reaction without enzyme as negative control. The standards (Std) of **8** and **9** were also analyzed. All the chromatograms show the extraction of calculated ion *m/z*=211.0379 $[M+Cl]^-$. For more details, see also Supplementary Table 4.

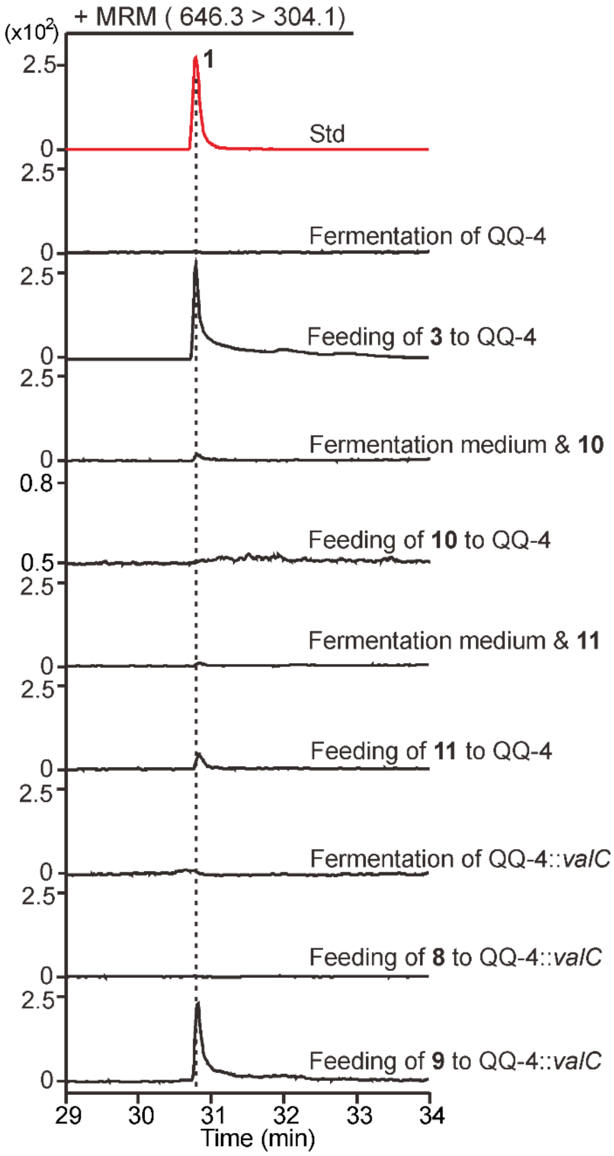

Supplementary Figure 20. Identification of the true intermediate for the biosynthesis of 1. HPLC-QQQ/MS analysis of **1** after feeding of **10** or **11** to QQ-4 or feeding of **8** or **9** to QQ-4::*valC*. Feeding of **3** to QQ-4 is positive control. The fermentation of QQ-4 (without feeding), fermentation medium with **10** (without inoculation), fermentation medium with **11** (without inoculation) and fermentation of QQ-4::*valC* (without feeding) are negative controls. Meanwhile, the standard (std) of **1** was also analyzed. In order to identify the true intermediate for biosynthesis of **1**, two methods, feeding of **10** or **11** to QQ-4 or feeding of **8** or **9** to QQ-4::*valC*, were initially carried out. Feeding of **11** to QQ-4 and feeding of **9** to QQ-4::*valC* resulted in the production of **1**. In addition,

the production of **1** was not detected after feeding of **10** to QQ-4 or feeding of **8** to QQ-4::*valC*. These results suggest that **11** is an intermediate for the biosynthesis of **1**. However, the production of **1** after feeding of **11** to QQ-4 is obviously lower than feeding of **9** to QQ-4::*valC*. Therefore, feeding of **8** or **9** to QQ-4::*valC* was a preferable method.

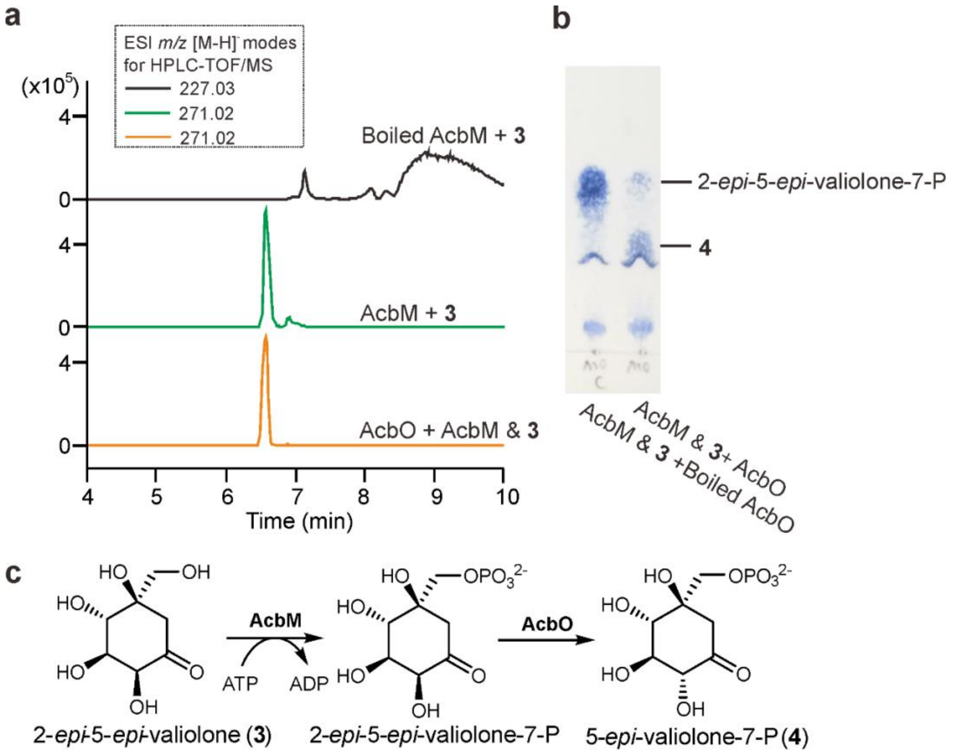

Supplementary Figure 21. Preparation of 4 from 3 by the sequential catalysis of AcbM and AcbO. a HPLC-TOF/MS analysis of the reaction products of boiled AcbM with **3** (as control), AcbM with **3** and AcbO with AcbM-catalyzed product. The chromatograms show the extraction of calculated ion *m/z*=227.0328 $[M-H]^-$ (**3**) or *m/z*=271.0224 $[M-H]^-$ (2-*epi*-5-*epi*-valiolone-7-P and **4**). **b** Thin layer chromatography (TLC) analysis of the catalytic product of AcbO. Similar results were obtained in three independent experiments. **c** Schematic illustration of the sequential catalysis of AcbM and AcbO[1,2].

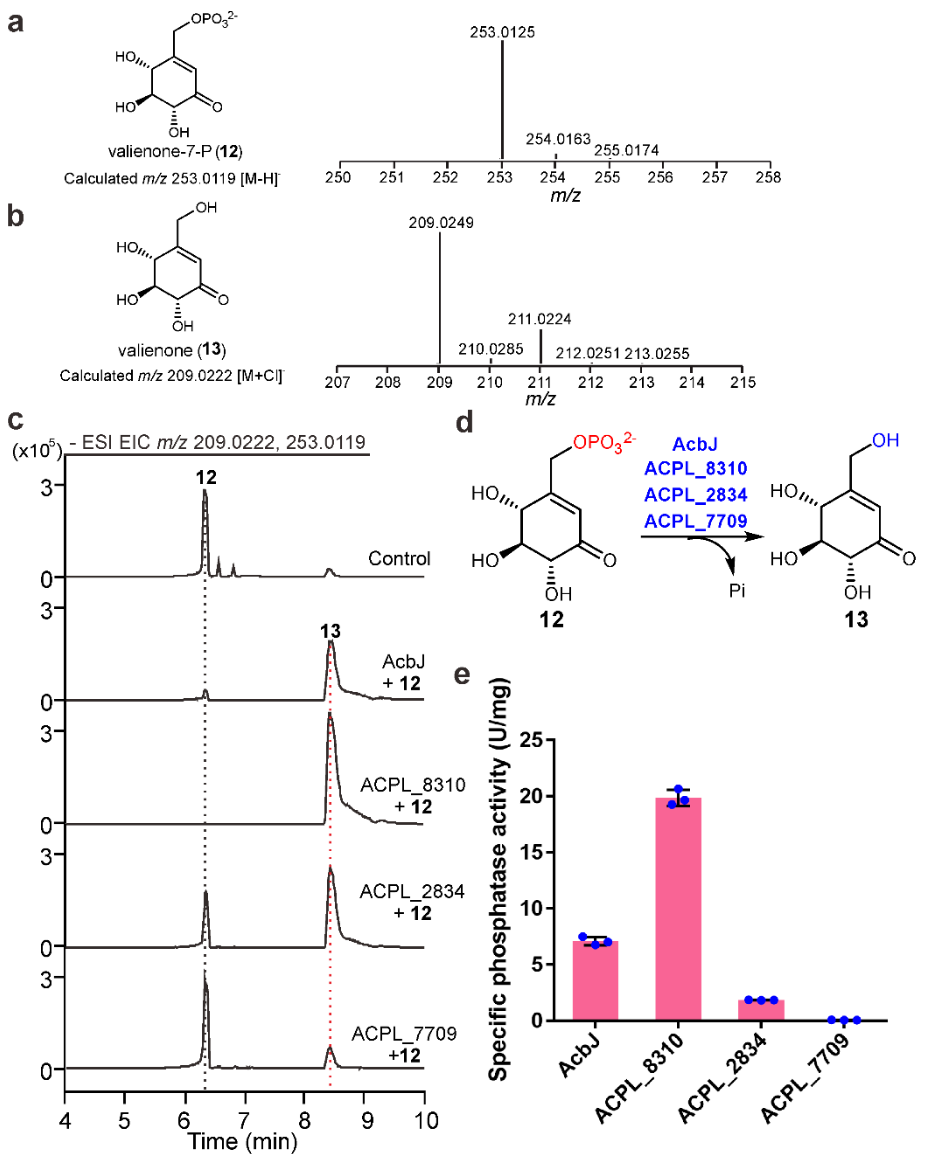

Supplementary Figure 22. Dephosphorylation of 12 to 13 by phosphatases. a, b The high resolution spectra of **12** and **13** analyzed by HPLC-TOF/MS. **c** HPLC-TOF/MS analysis of the *in vitro* dephosphorylation of **12** by AcbJ, ACPL_8310, ACPL_2834 or ACPL_7709, with the reaction without enzyme as negative control. All the chromatograms show the simultaneous extraction of calculated ion *m/z*=209.0222 [M+Cl]- and *m/z*=253.0119 [M-H]-. **d** Schematic illustration of the dephosphorylation of **13** to **12** by AcbJ, ACPL_8310, ACPL_2834 or ACPL_7709. **e** The specific activities of AcbJ, ACPL_8310, ACPL_2834 or ACPL_7709 on **12**. Error bars, mean ± SD (n=3 biological replicates).

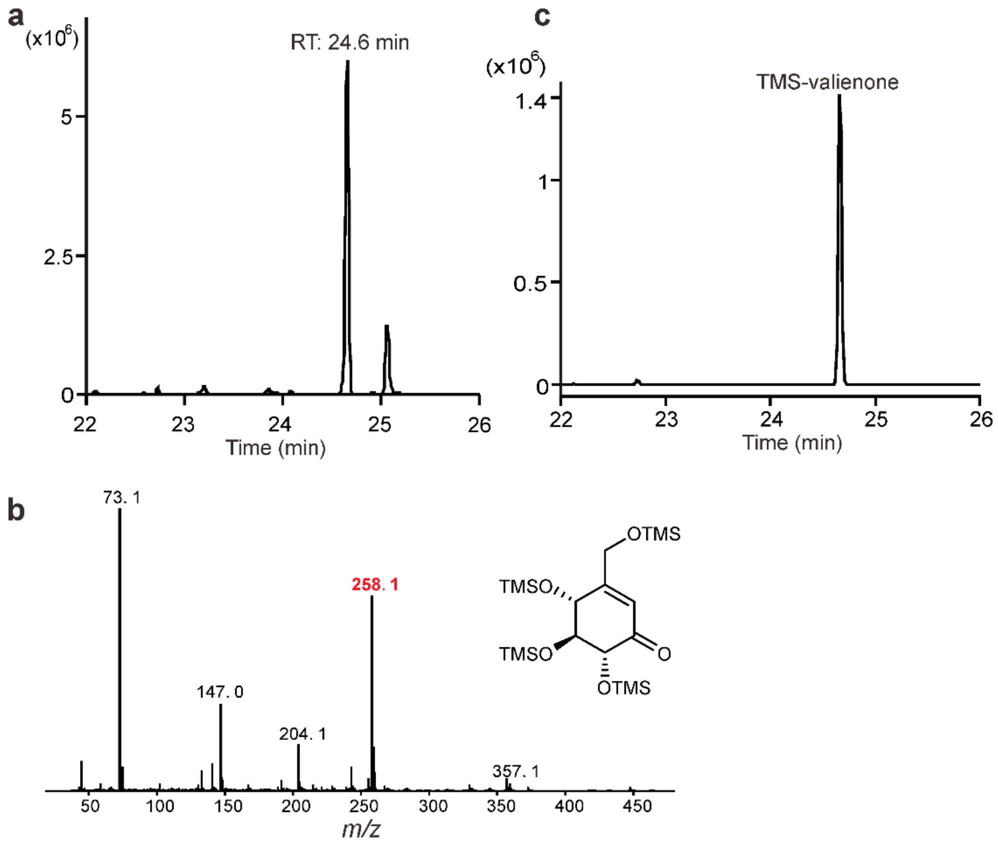

Supplementary Figure 23. GC-QMS analysis of standard 13 after derivatization by BSTFA. a The total ion chromatogram of TMS-valienone. The retention time (RT) is 24.6 min. **b** The mass spectrum of TMS-valienone. The unique product ion *m/z*=258.1 was chosen for quantitative and qualitative analysis. **c** The chromatogram shows the extraction of unique product ion *m/z*=258.1. TMS is the abbreviation of trimethyl silicyl.

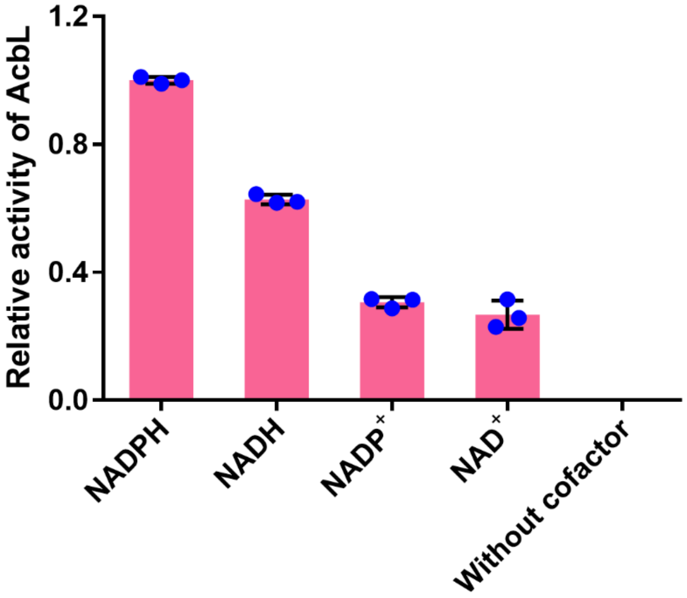

Supplementary Figure 24. The optimal cofactors involved in the conversion of 4 to 12 catalyzed by AcbL. The average enzyme activity of AcbL with NADPH is set to 1 as standard, and the activities with NADH, $NADP^+$ and NAD^+ are accordingly calculated. Error bars, mean ± SD (n=3 biological replicates).

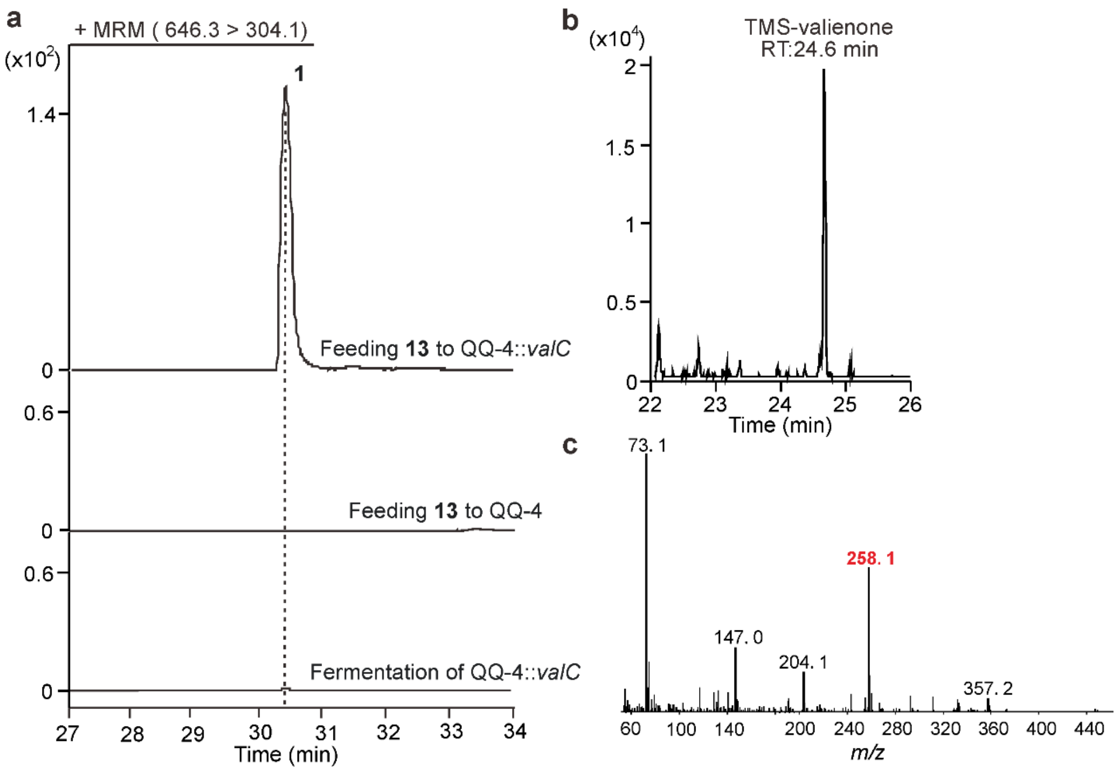

Supplementary Figure 25. Valienone is the shunt product derived from intermediate 12 by dephosphorylation. **a** HPLC-QQQ/MS analysis of **1** after feeding valienone (**13**) to the mutant QQ-4 and QQ-4::*valC*, and the fermentation of QQ-4::*valC* without feeding is set as negative control. **b** GC-QMS analysis of the fermentation broth of *Actinoplanes* sp. SE50/110 after derivatization by BSTFA. The chromatogram shows the extraction of unique product ion *m/z*=258.1. **c** The mass spectrum of TMS-valienone. TMS is the abbreviation of trimethyl silicyl. The retention time and mass spectrum of TMS-valienone in the fermentation broth of *Actinoplanes* sp. SE50/110 are in accordance with the standard shown in Supplementary Fig. 23.

a

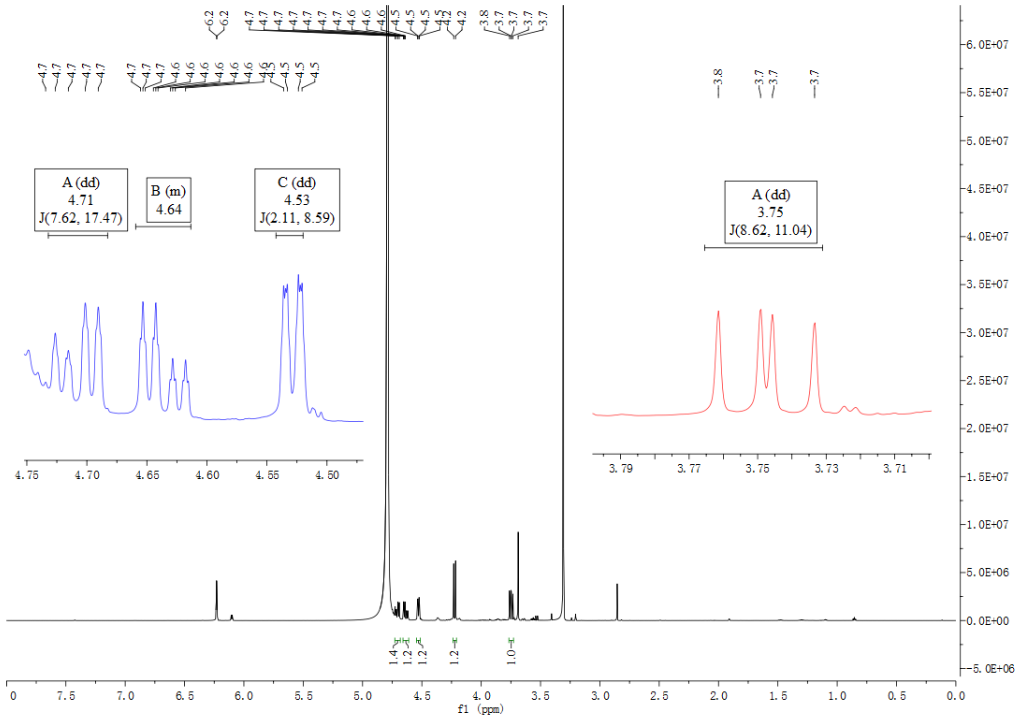

b

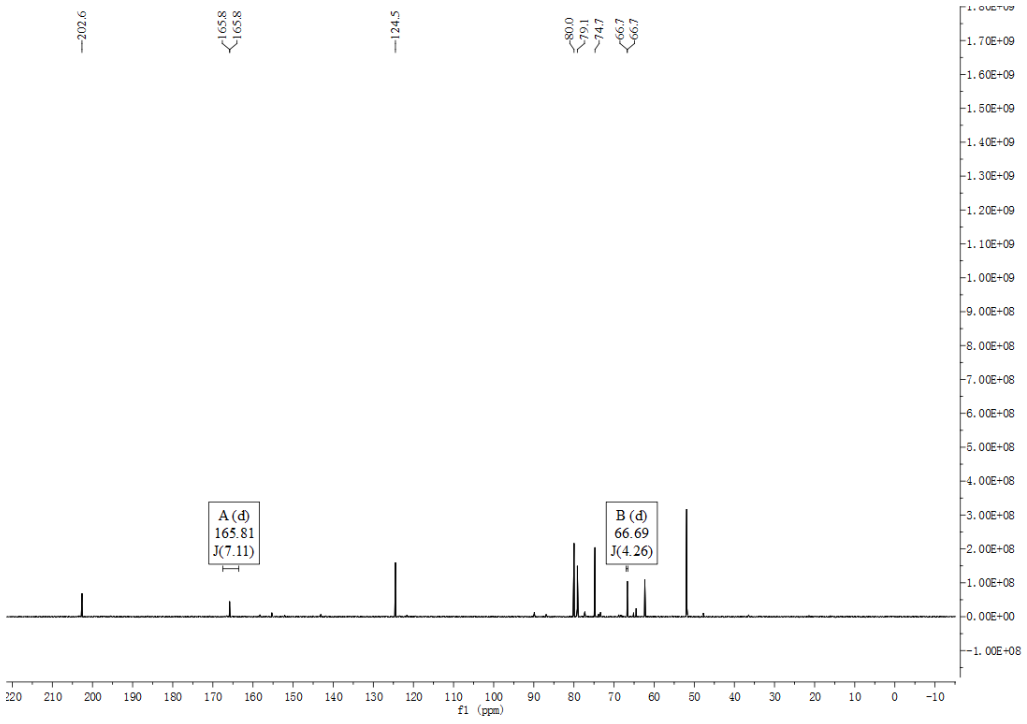

Supplementary Figure 26. ^{1}H and ^{13}C NMR spectra of 12. **a** ^{1}H NMR spectrum. **b** ^{13}C NMR spectrum. The NMR spectra were collected in D_2O at 700 (^{1}H NMR) and 175 MHz (^{13}C NMR) on Bruker Avance NEO 700 spectrometer (16.44 T).

a

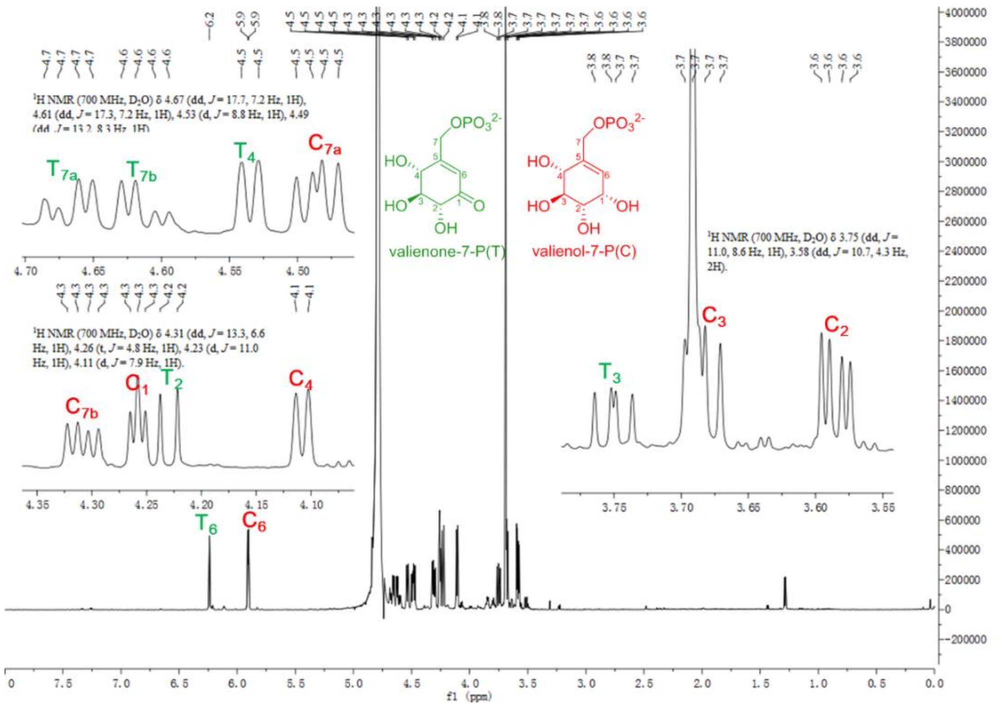

b

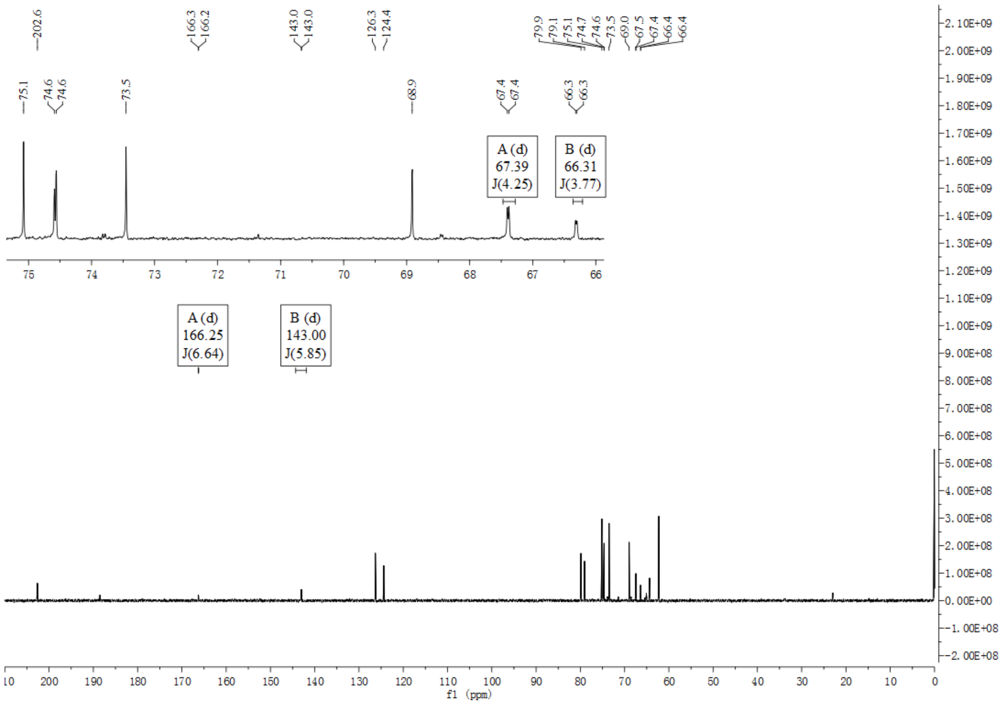

Supplementary Figure 27. Blended ^{1}H and blended ^{13}C NMR spectra of AcbN-catalyzed product using 12 as substrate. a Blended ^{1}H NMR spectrum of **12** and **11**. **b** Blended ^{13}C NMR spectrum of **12** and **11**. The NMR spectra were collected in D_2O at 700 (^{1}H NMR) and 175 MHz (^{13}C NMR) on Bruker Avance NEO 700 spectrometer (16.44 T). Comparison of 1D NMR data

of valienol-7-P (**11**) (Supplementary Fig. 16) and valienone-7-P (**12**) (Supplementary Fig. 26) with the blended NMR spectra of **12** and **11** suggested that valienol-7-P (**11**) is the main component (reaction product) in that mixture, in which **12** is the residual substrate.

a

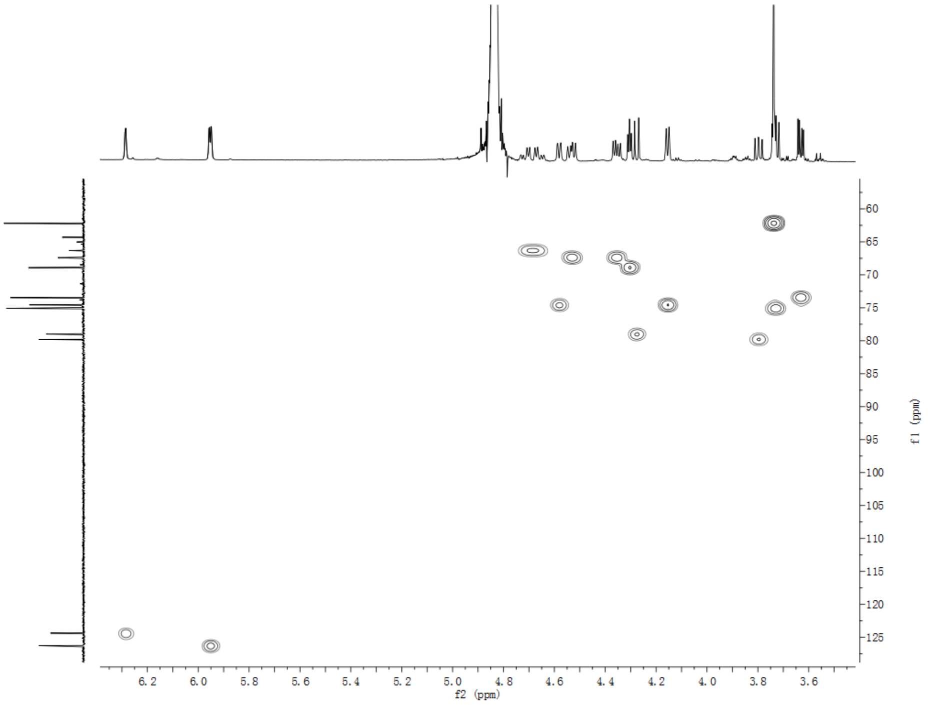

b

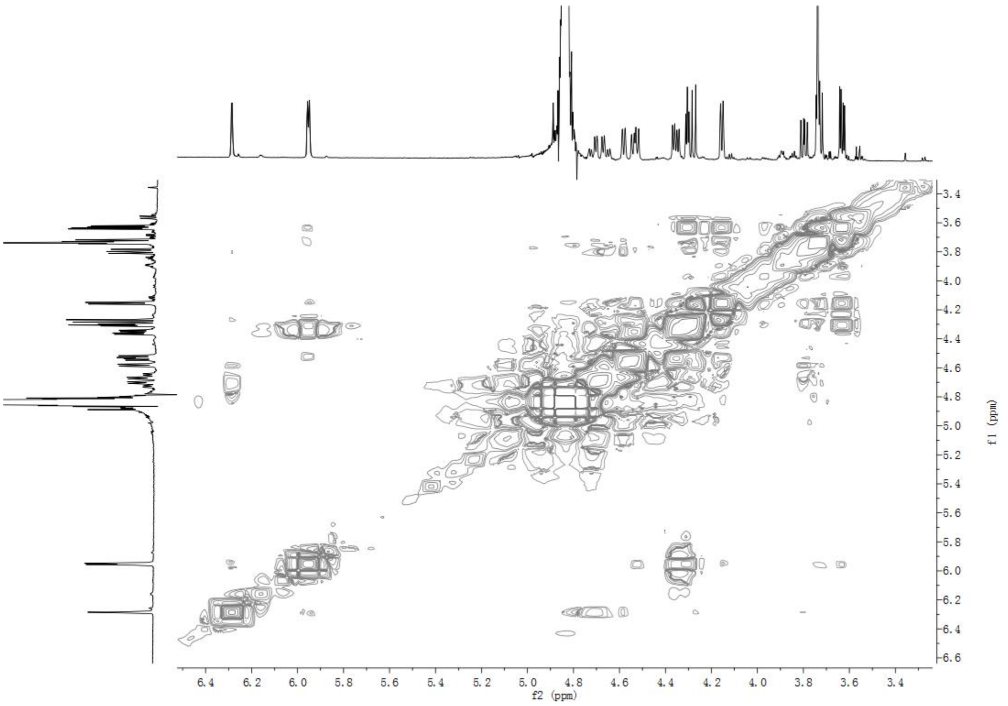

Supplementary Figure 28. Blended HSQC and blended NOESY spectra of AcbN-catalyzed product using 12 as substrate. a Blended HSQC spectrum of **12** and **11**. **b** Blended NOESY spectrum of **12** and **11**. The NMR spectra were collected in D_2O at 700 (^1H NMR) and 175 MHz (^{13}C NMR) on Bruker Avance NEO 700 spectrometer (16.44 T).

a

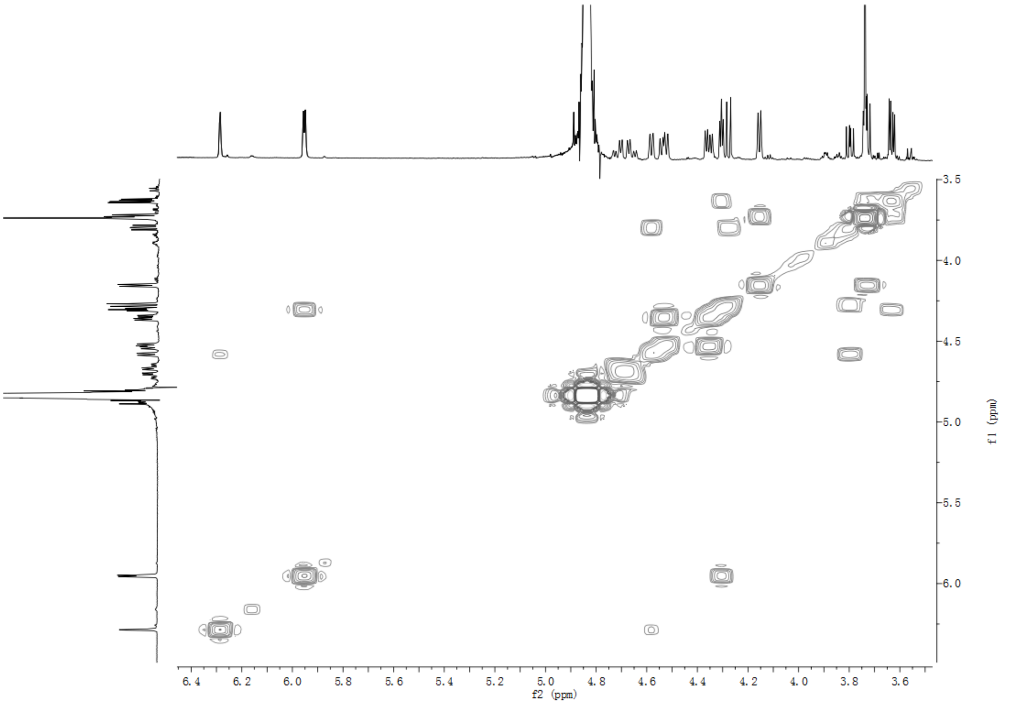

b

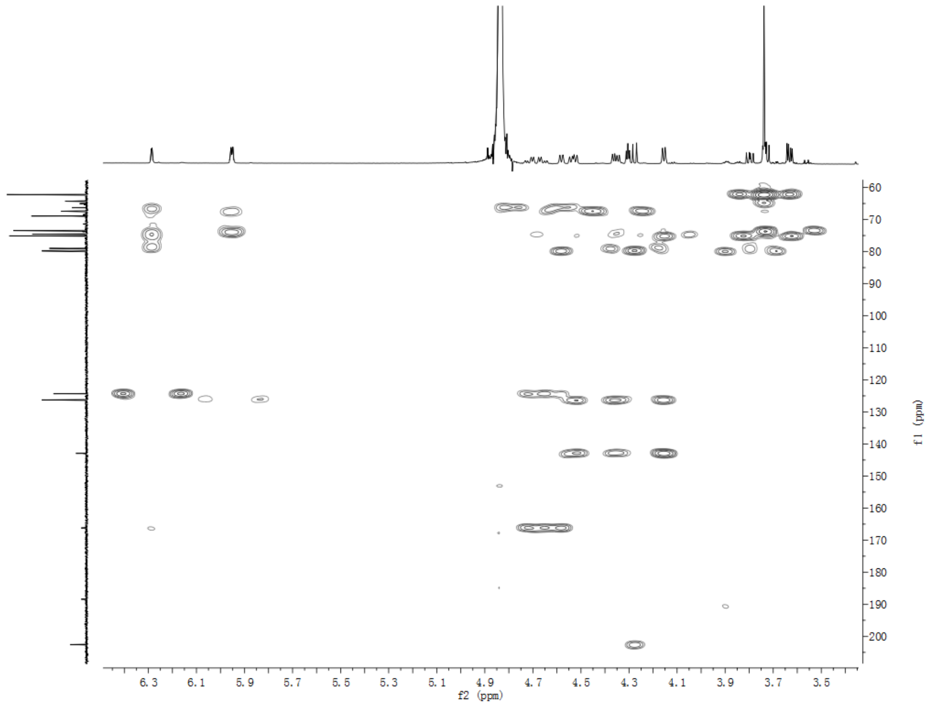

Supplementary Figure 29. Blended ^{1}H-^{1}H COSY and blended HMBC spectra of AcbN-catalyzed product using 12 as substrate. a Blended ^{1}H-^{1}H COSY spectrum of **12** and **11**. **b** Blended HMBC spectrum of **12** and **11**. The NMR spectra were collected in D_2O at 700 (^{1}H NMR) and 175 MHz (^{13}C NMR) on Bruker Avance NEO 700 spectrometer (16.44 T).

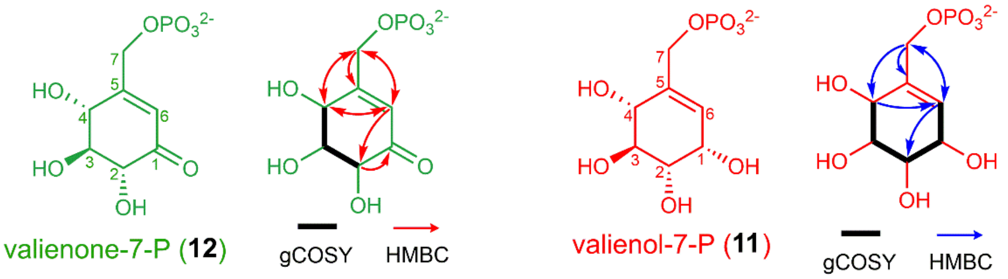

Supplementary Figure 30. Key 2D NMR correlations for 12 and 11. For more details, see also Supplementary Fig. 27-29.

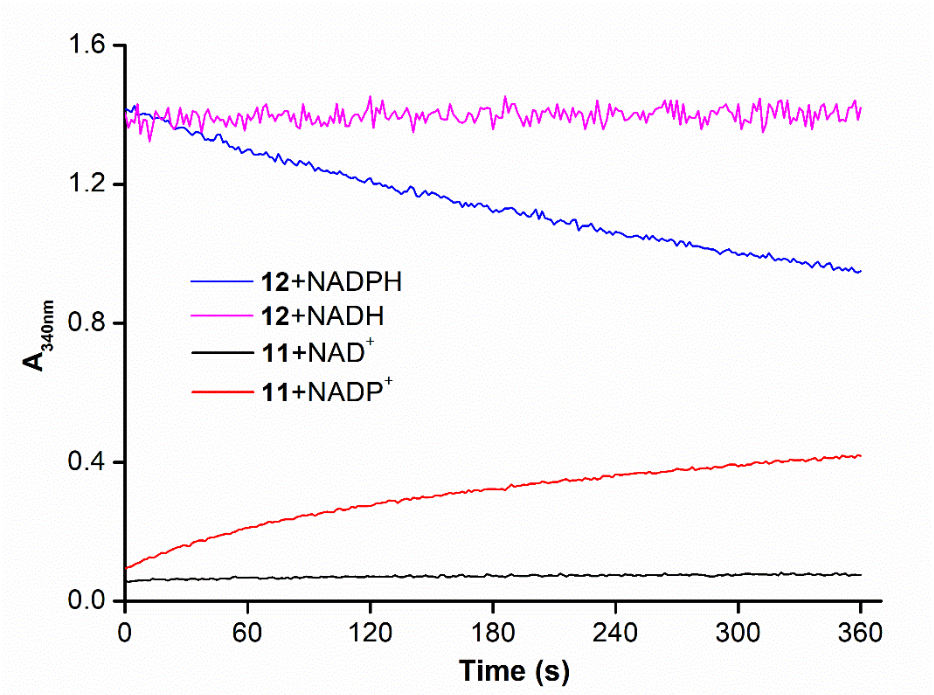

Supplementary Figure 31. The optimal cofactors involved in the reversible conversion between 12 and 11 catalyzed by AcbN. The blue and plum lines show the oxidation of NADPH and NADH, respectively, when incubated with AcbN and **12**. The red and black lines show the reduction of $NADP^+$ and NAD^+, respectively, when incubated with AcbN and **11**.

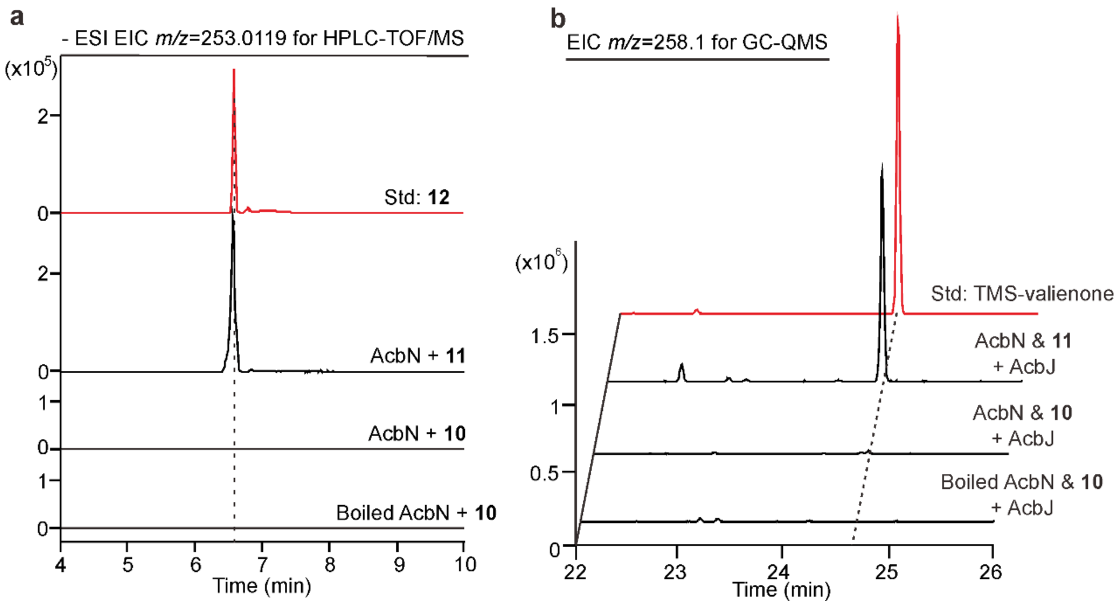

Supplementary Figure 32. Analysis of the reaction products catalyzed by AcbN using 10 as substrate. a HPLC-TOF/MS analysis of the reaction products of AcbN with **11** (as a positive control), AcbN with **10** and boiled AcbN with **10** (as a negative control). The standard (std) of **12** was also analyzed. All the chromatograms show the extraction of calculated ion *m/z*=253.0119 $[M-H]^-$ of **12**. **b** GC-QMS further determination of the reaction products after the dephosphorylation by AcbJ. AcbJ was added to the reaction mixtures of AcbN & **11**, AcbN & **10** and boiled AcbN & **10**. The standard (std) of **13** was also analyzed after derivatization by BSTFA. TMS is the abbreviation of trimethyl silicyl. All the chromatograms show the extraction of unique product ion *m/z*=258.1 of TMS-valienone. To investigate whether **10** is a substrate for AcbN, purified **10** was incubated with AcbN and $NADP^+$. **12** (*m/z*=253.0119 $[M-H]^-$) was not detected by HPLC-TOF/MS. Then, the reaction mixture was further incubated with AcbJ, and the dephosphorylated product **13** was not detected by GC-QMS. These results indicated that AcbN could not catalyze the conversion of **10** to **12**.

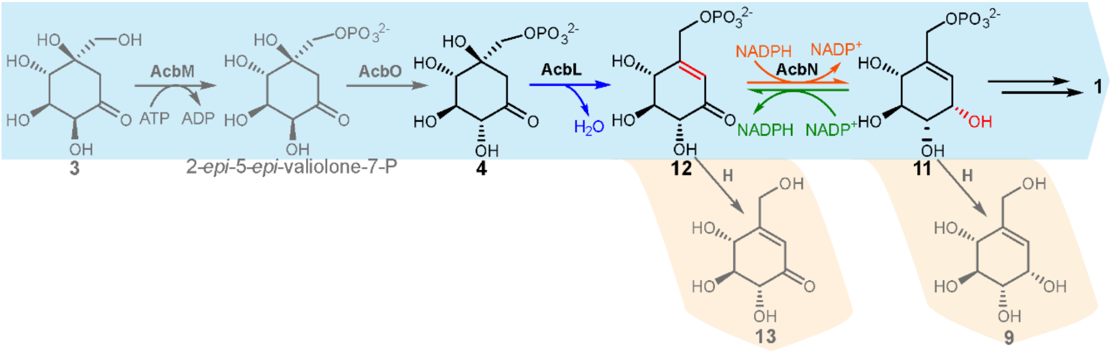

Supplementary Figure 33. Schematic illustration of the AcbL- and AcbN-catalyzed conversions. AcbL catalyzes the dehydration of **4** to **12**, AcbN catalyzes the reversible conversion between **12** and **11**, and the involved hydrolyases (H) catalyze the dephosphorylation of biosynthetic intermediates **11** and **12** to shunt products **9** and **13**, respectively. H: AcbJ, ACPL_8310, ACPL_2834, ACPL_7709, etc.

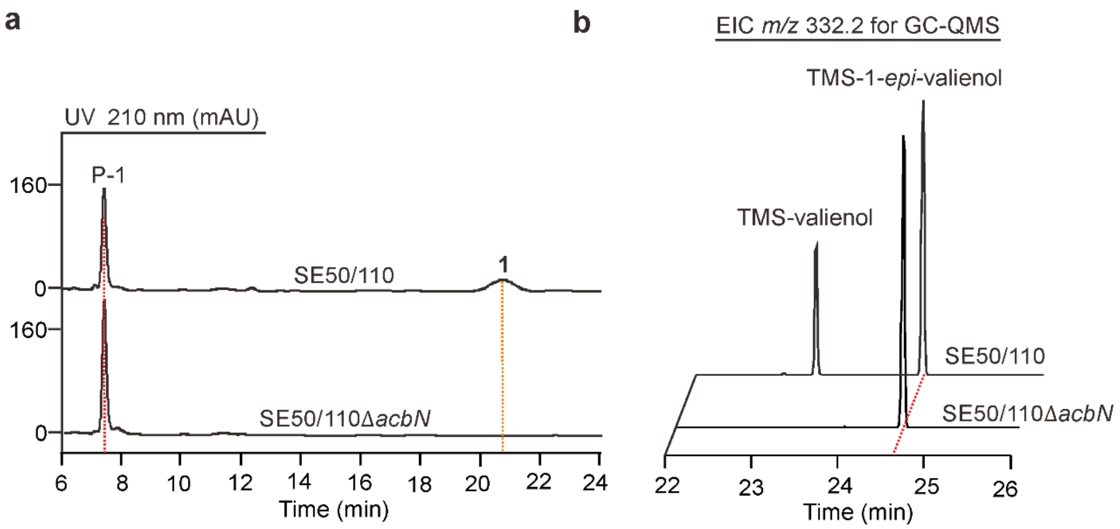

Supplementary Figure 34. Inactivation of *acbN* in *Actinoplanes* sp. SE50/110. a HPLC profiles of SE50/110 and SE50/110Δ*acbN*. **b** GC-QMS analysis of the fermentation product of SE50/110 and SE50/110Δ*acbN*. The chromatograms show the extraction of unique product ion *m/z*=332.2 of TMS-1-*epi*-valienol or TMS-valienol. TMS is the abbreviation of trimethyl silicyl.

a

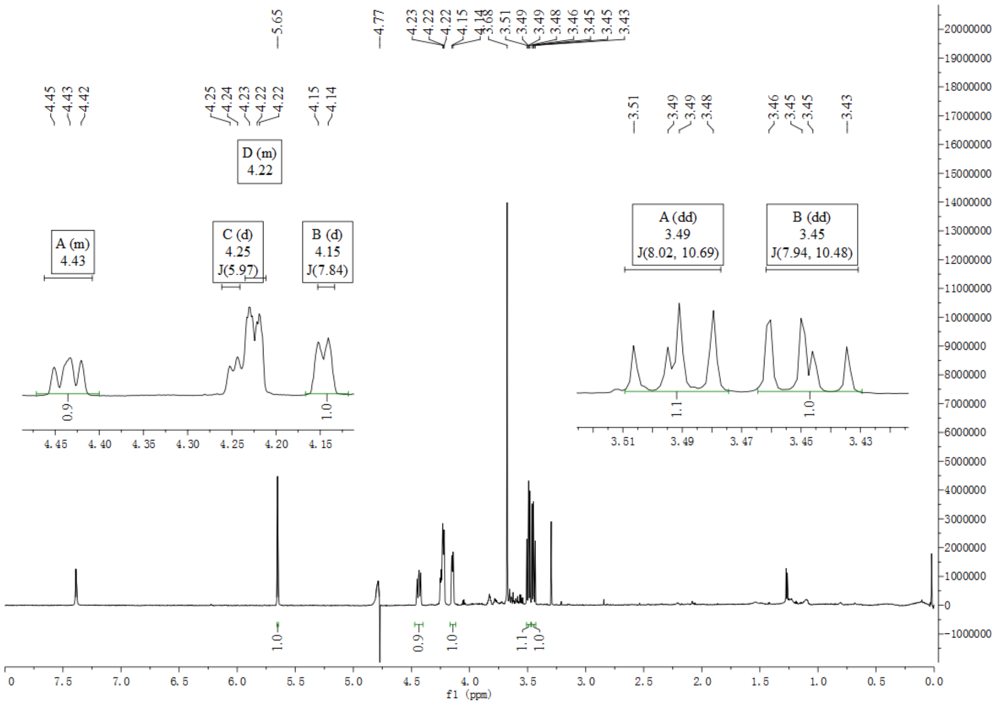

b

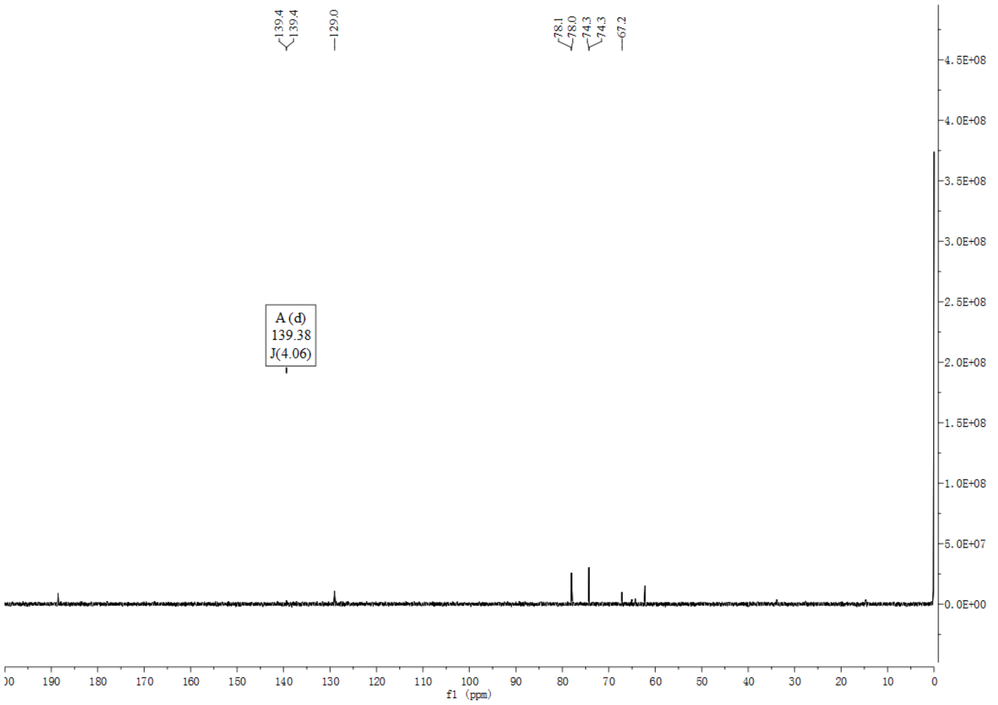

Supplementary Figure 35. ^{1}H and ^{13}C NMR spectra of 10. a ^{1}H NMR spectrum of **10**. **b** ^{13}C NMR spectrum of **10**. The NMR spectra were collected in D_2O at 700 (^{1}H NMR) and 175 MHz (^{13}C NMR) on Bruker Avance NEO 700 spectrometer (16.44 T). **10** was obtained through the catalysis by cell-free extract of mutant QQ-3 using **12** as substrate.

a

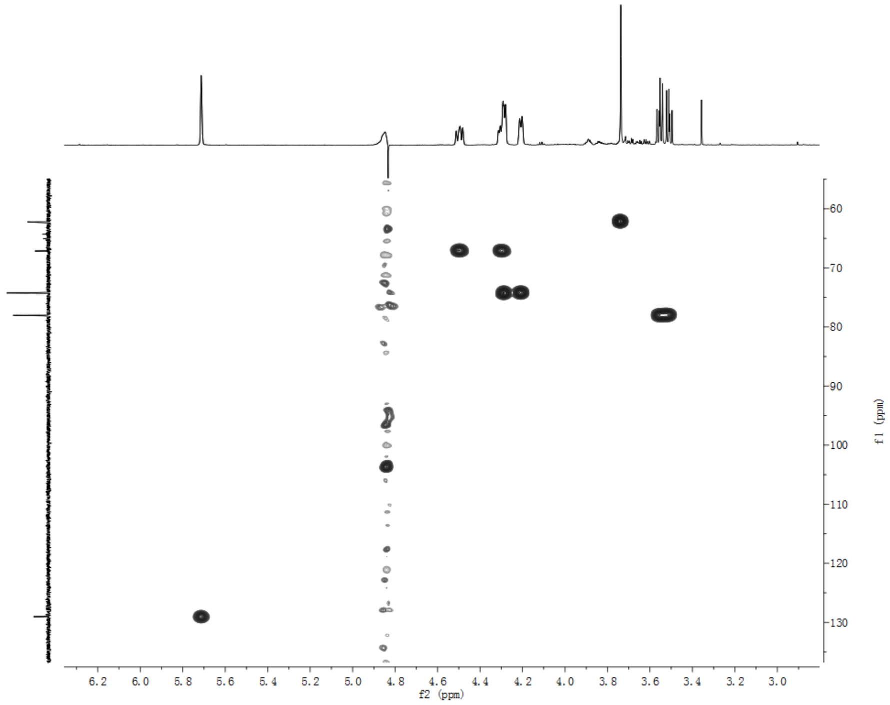

b

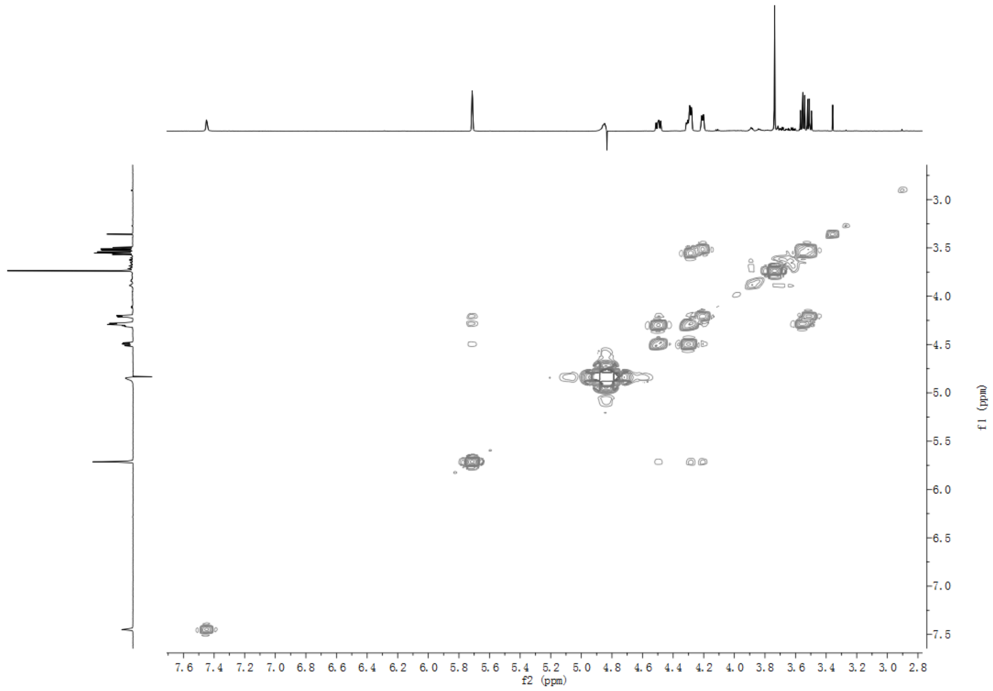

Supplementary Figure 36. HSQC and ^{1}H-^{1}H COSY spectra of 10. a HSQC spectrum of **10**. **b** ^{1}H-^{1}H COSY spectrum of **10**. The NMR spectra were collected in D_2O at 700 (^{1}H NMR) and 175 MHz (^{13}C NMR) on Bruker Avance NEO 700 spectrometer (16.44 T).

a

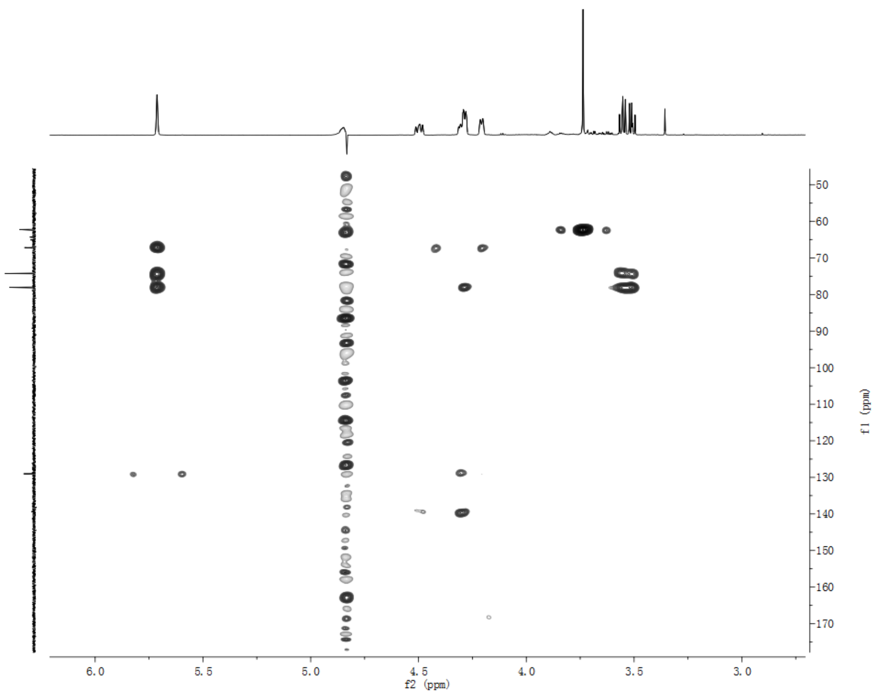

b

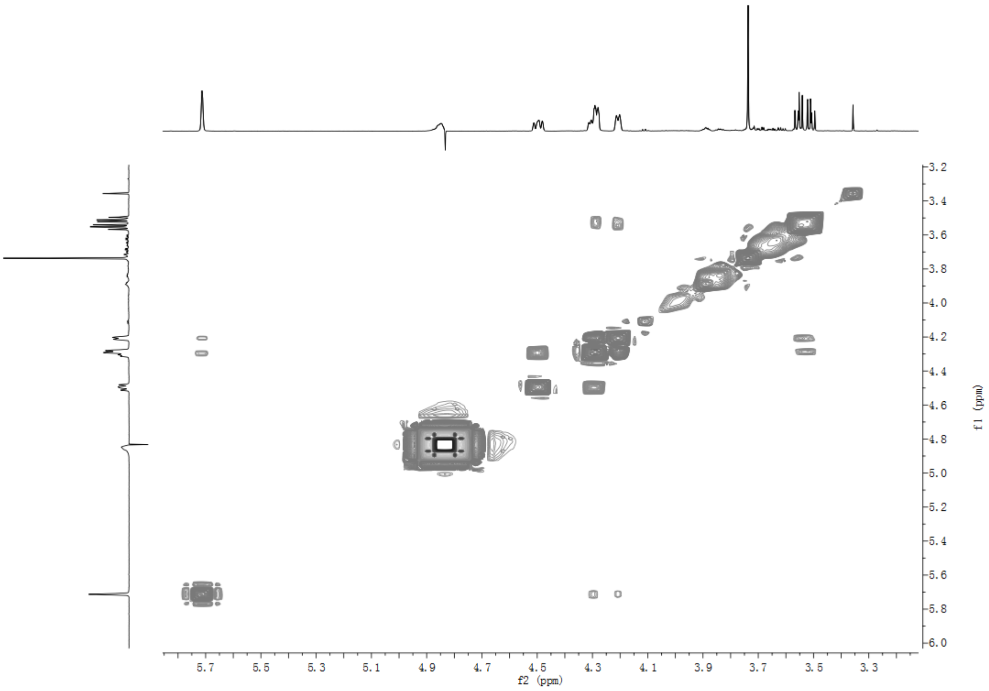

Supplementary Figure 37. HBMC and NOESY spectra of 10. a HBMC spectrum of **10**. **b** NOESY spectrum of **10**. The NMR spectra were collected in D_2O at 700 (^{1}H NMR) and 175 MHz (^{13}C NMR) on Bruker Avance NEO 700 spectrometer (16.44 T).

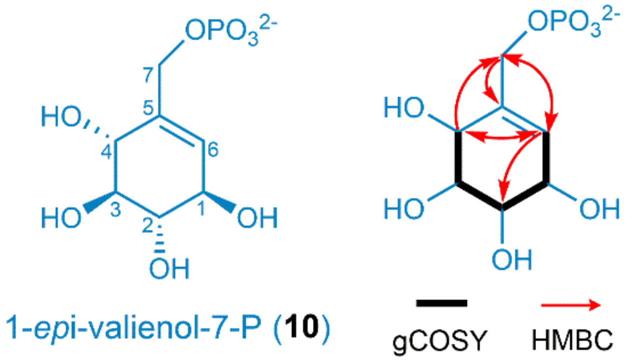

Supplementary Figure 38. Key 2D NMR correlations for 10. For more details, see also Supplementary Fig. 35-37.

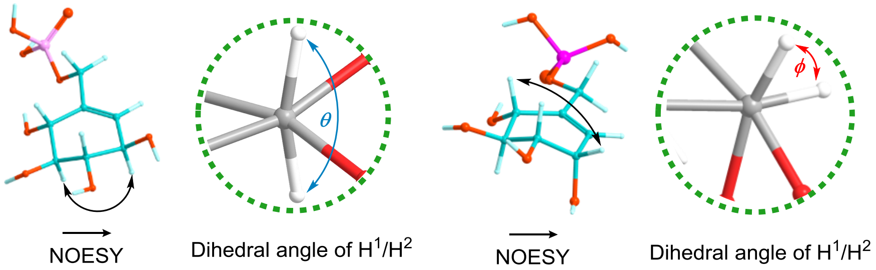

Supplementary Figure 39. Illustration of the C-1 hydroxyl group orientations of 10 and 11. Until now, the utmost important issue is the relative configuration of the C-1 hydroxyl group because it is the only one variation between **10** and **11**. To address this structural difference, calculation of dihedral angles in a specific region and analysis of NOE signals had been performed. Notably, the diagnostic peaks of H-1/H-3 in **10** and H-1/H-4 in **11** shed lights on the exact relative configuration of C-1 hydroxyl group, namely *β*-orientation in **10** and *α*-orientation in **11**. Meanwhile, the vital details of dihedral angles of H^1/H^2 were established by careful analysis of chemical models (θ = 174.3º in **10**, φ = 43.9º in **11**). According to the Karplus equation[3], *J*-coupling constants (7.8 Hz of $^3J_{H1/H2}$ in **10**, 4.8 Hz of $^3J_{H1/H2}$ in **11**) in ^{1}H NMR spectra had indisputably confirmed the aforementioned C-1 hydroxyl group orientation. For more details, see also Supplementary Fig. 27-30 and Supplementary Fig. 35-38.

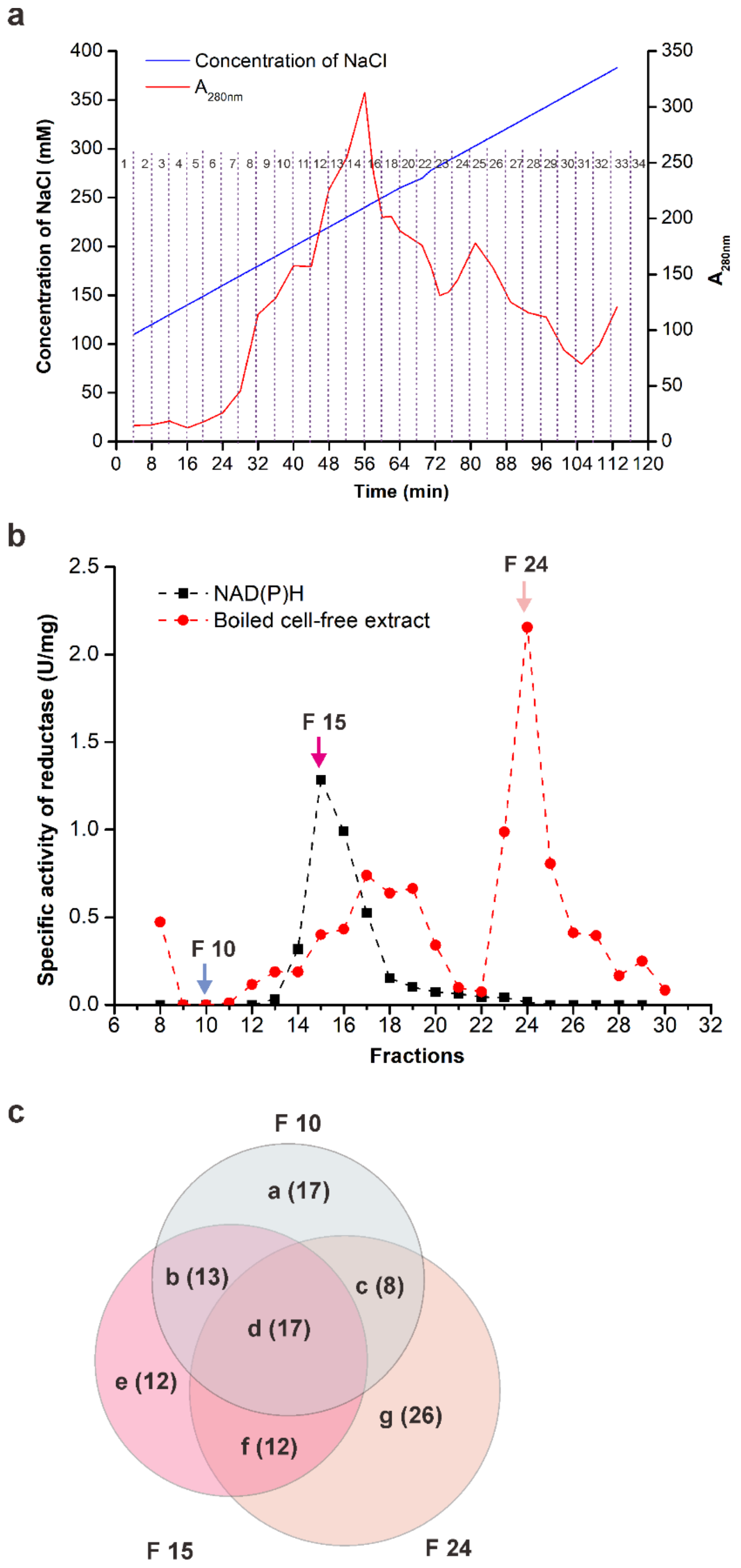

Supplementary Figure 40. Screening of reductases involved in the conversion of 12 to 10. a Fractionation of the total cellular proteins of QQ-3 to 34 fractions by anion exchange column HiTrap Q HP with a linear gradient elution by increasing the NaCl concentrations from 100 to 400 mM. $A_{280\ nm}$

shows the concentrations of total proteins. **b** Tracing of the reductase activity of 8-30 fractions in the presence of NADPH or BCF. F15, F24 and F10 (as control) were identified by Q Exactive™ hybrid quadrupole-Orbitrap mass spectrometer. **c** 54, 64 and 55 oxidoreductases with high reliability and abundance (Unique PepCount⩾4) were selected and classified from F15, F24 and F10, respectively. Among the 12 (group e) and 26 (group g) unique proteins in F15 and F24, 8 and 10 oxidoreductases with higher reliability and transcription were characterized *in vitro*, respectively (Supplementary Table 7). a (17): 17 proteins unique in F10; b (13): 13 proteins in both F10 and F15; c (8): 8 proteins in both F10 and F24; d (17): 17 proteins in all three fractions. e (12): 12 proteins unique in F15; f (12) 12 proteins in both F15 and F24; g (16): 16 proteins unique in F24.

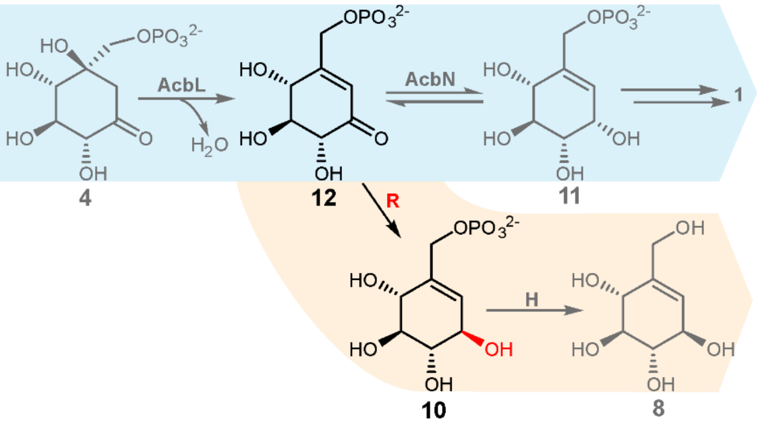

R: ACPL_7966, ACPL_7511, ACPL_5736, ACPL_7667, and etc.

Supplementary Figure 41. Schematic illustration of the biosynthesis of shunt product 8.

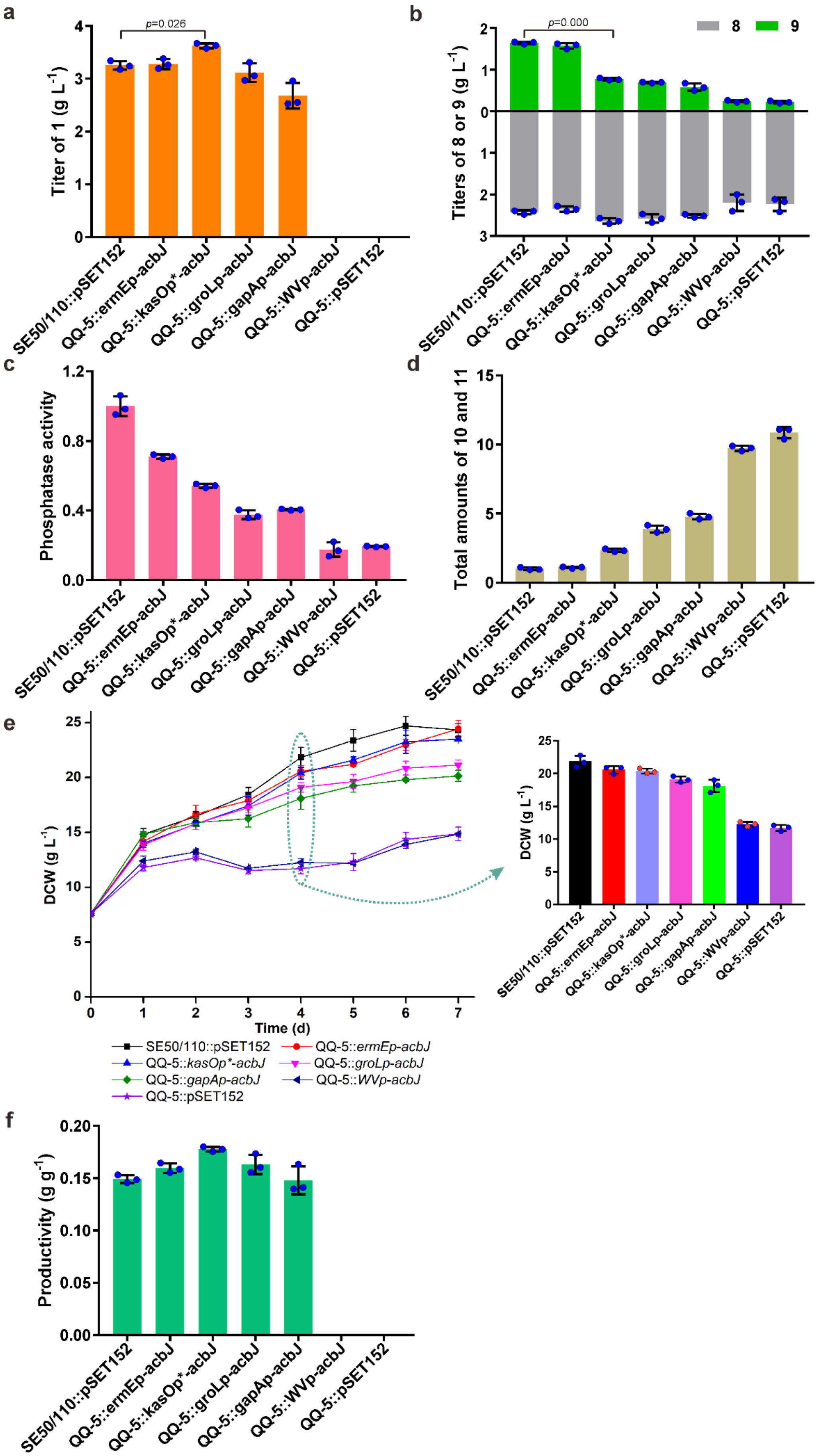
a
p=0.026
Titer of 1 (g L-1)
b
p=0.000
8
9
Titers of 8 or 9 (g L-1)
c
Phosphatase activity
d
Total amounts of 10 and 11
e
DCW (g L-1)
Time (d)
SE50/110::pSET152
QQ-5::ermEp-acbJ
QQ-5::kasOp*-acbJ
QQ-5::groLp-acbJ
QQ-5::gapAp-acbJ
QQ-5::WVp-acbJ
QQ-5::pSET152
f
Productivity (g g-1)

Supplementary Figure 42. Tuning down the transcription of *acbJ* using a series of promoters with less strength. The pSET152-derived plasmids, inserted with *acbJ* under the control of promoters *ermEp**, *kasOp**, *groLp*, *gapAp* or *WVp*, were constructed and introduced into QQ-5. The mutant SE50/110::pSET152 and QQ-5::pSET152 are positive and negative controls, respectively. **a, b** The titers of **1**, **8** and **9** of the involved strains after 4-day fermentation. **c, d** The phosphatase activity and total amounts of endogenous **10** and **11** of the involved strains after 2-day fermentation. **e** The time courses of growth (including the biomass of day 4) of the involved strains. **f** Calculation of the productivity (ratio of titer of **1** and DCW) of **1** after 4-day fermentation. Two-tailed paired t-tests. DCW is the abbreviation of dry cell weight. Error bars, mean ± SD (n=3 biological replicates).

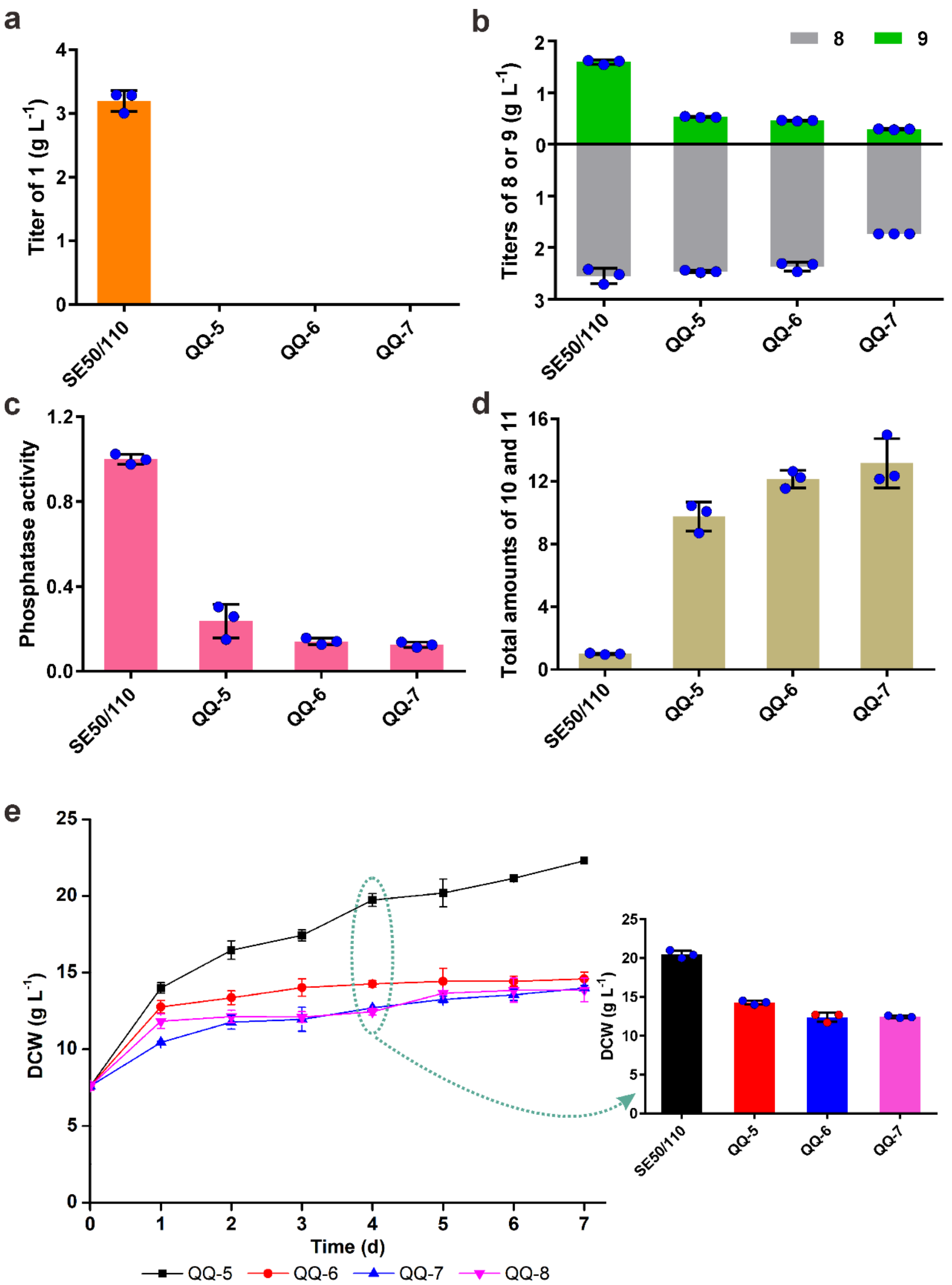

Supplementary Figure 43. Minimization of the *in vivo* phosphatase activities. a, b The amounts of **1**, **8** and **9** of *Actinoplanes* sp. SE50/110 (abbreviated as SE50/1110), QQ-5, QQ-6 and QQ-7 after 4-day fermentation. **c, d** Analysis of the phosphatase activity and total amounts of endogenous **10**

and **11** of the involved strains after 2-day fermentation. **e** Time courses of growth (including the biomass of day 4) of the involved strains. QQ-6: deletion of *ACPL_8310* in QQ-5; QQ-7: down-regulation of the expression of *ACPL_2834* by replacing the native promoter with a weaker promoter *WVp* in QQ-6. DCW is the abbreviation of dry cell weight. Error bars, mean ± SD (n=3 biological replicates). ACPL_2834 is proposed as β-phosphoglucomutase using BLASTP program[4]. We were failed to obtain a gene-deleted mutant after numerous attempts. Alternatively, we replaced its native promoter with a weaker *WVp* promoter to down-regulate its transcription. While successive deletion of *ACPL_8310* and down-regulation of the transcription of *ACPL_2834*, further decreased accumulations of **8** and **9** and phosphatase activity and increased total amounts of endogenous **10** and **11** were observed. However, the deletion and down-regulation of the transcription of *ACPL_7709* (encoding trehalose 6-phosphate phosphatase responsible for trehalose biosynthesis) resulted in low cell growth and titer of **1**. In consideration of the low catalytic activity (Supplementary Fig. 18) and the important role in primary metabolism, the expression level of *ACPL_7709* is not modulated in this study.

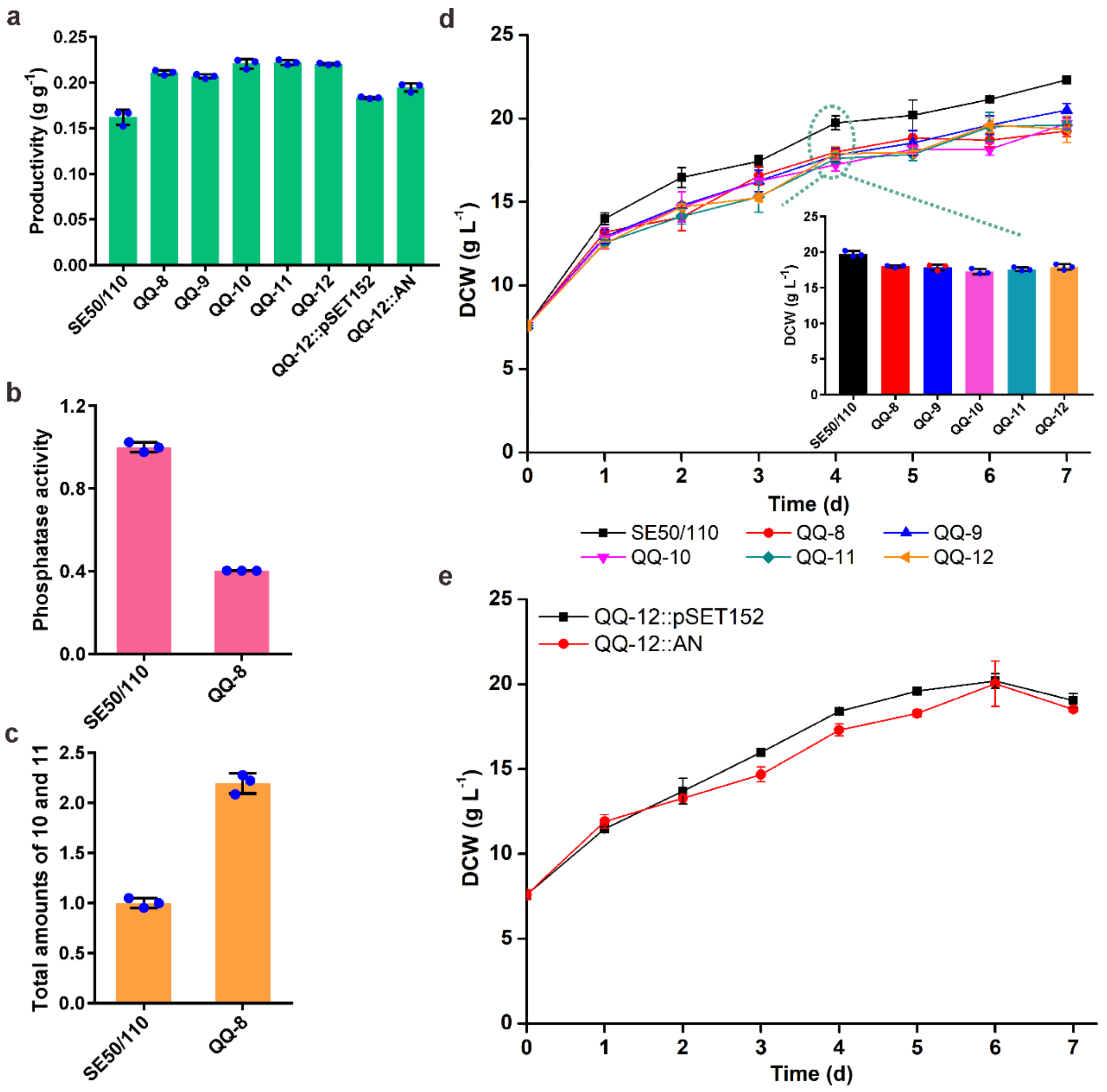

Supplementary Figure 44. Diverting the metabolic flux of shunt products toward 1. a The productivity (ratio of titer of **1** and DCW) of **1** of SE50/110 and the involved mutants. **b, c** Analysis of the phosphatase activity and total amounts of endogenous **10** and **11** of *Actinoplanes* sp. SE50/110 (abbreviated as SE50/110) and QQ-8 after 2-day fermentation. **d** The time courses of growth (including the biomass of day 2) of SE50/110, QQ-8, QQ-9, QQ-10, QQ-11 and QQ-12. **e** The time courses of growth of QQ-12::pSET152 and QQ-12::*AN*. DCW is the abbreviation of dry cell weight. Error bars, mean ± SD (n=3 biological replicates).

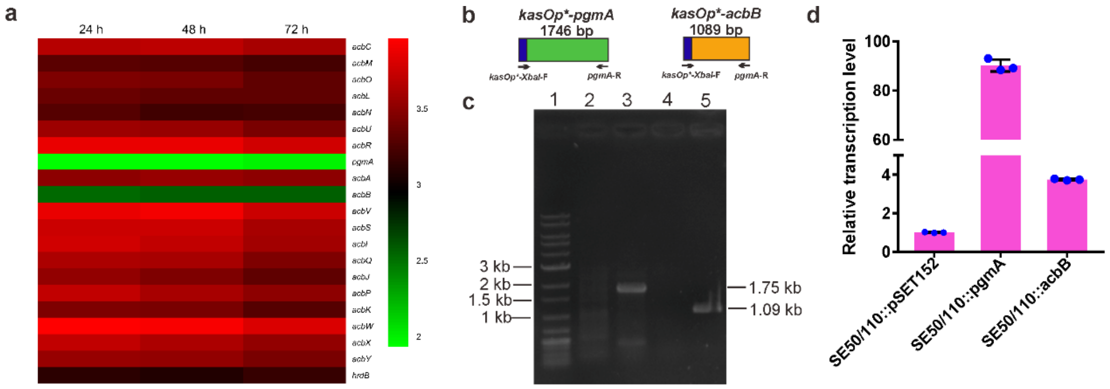

Supplementary Figure 45. Identification of the rate-limiting factors for the production of 1. a Analysis of the transcription of genes involved in the biosynthesis of **1** after fermentation for 24 h, 48 h and 72 h by RNA-seq. The transcription is shown as log10 conversion. Red and green represent high and low transcription, respectively. **b** Construction of the pSET152-derived plasmids containing the cassettes of *kasOp*-pgmA* or *kasOp*-acbB*. **c** Confirmation of the mutants SE50/110::*pgmA* and SE50/110::*acbB* by PCR amplification. Using primers *kasOp*-XbaI*-F/*pgmA*-R and *kasOp*-XbaI*-F/*acbB*-R, a 1.75-kb and 1.09-kb fragments were amplified by using the genomic DNA of SE50/110::*pgmA* and SE50/110::*acbB* as templates, respectively, while the genomic DNA of SE50/110::pSET152 (negative control) gave no amplified product. The PCR products of *kasOp*-pgmA* and *kasOp*-acbB* were further confirmed by DNA sequencing. 1: DNA marker; 2: SE50/110::pSET152 (*kasOp*-XbaI*-F/*pgmA*-R); 3: SE50/110::*pgmA*; 4: SE50/110::pSET152 (*kasOp*-XbaI*-F/*acbB*-R); 5: SE50/110::*acbB*. **d** Analysis of the relative transcription of *pgmA* and *acbB* in the corresponding strains after 2-day fermentation. The average transcription of genes in SE50/110::pSET152 is set to 1 as standard, and the transcription of genes in related mutants is accordingly calculated. Error bars, mean ± SD (n=3 biological replicates).

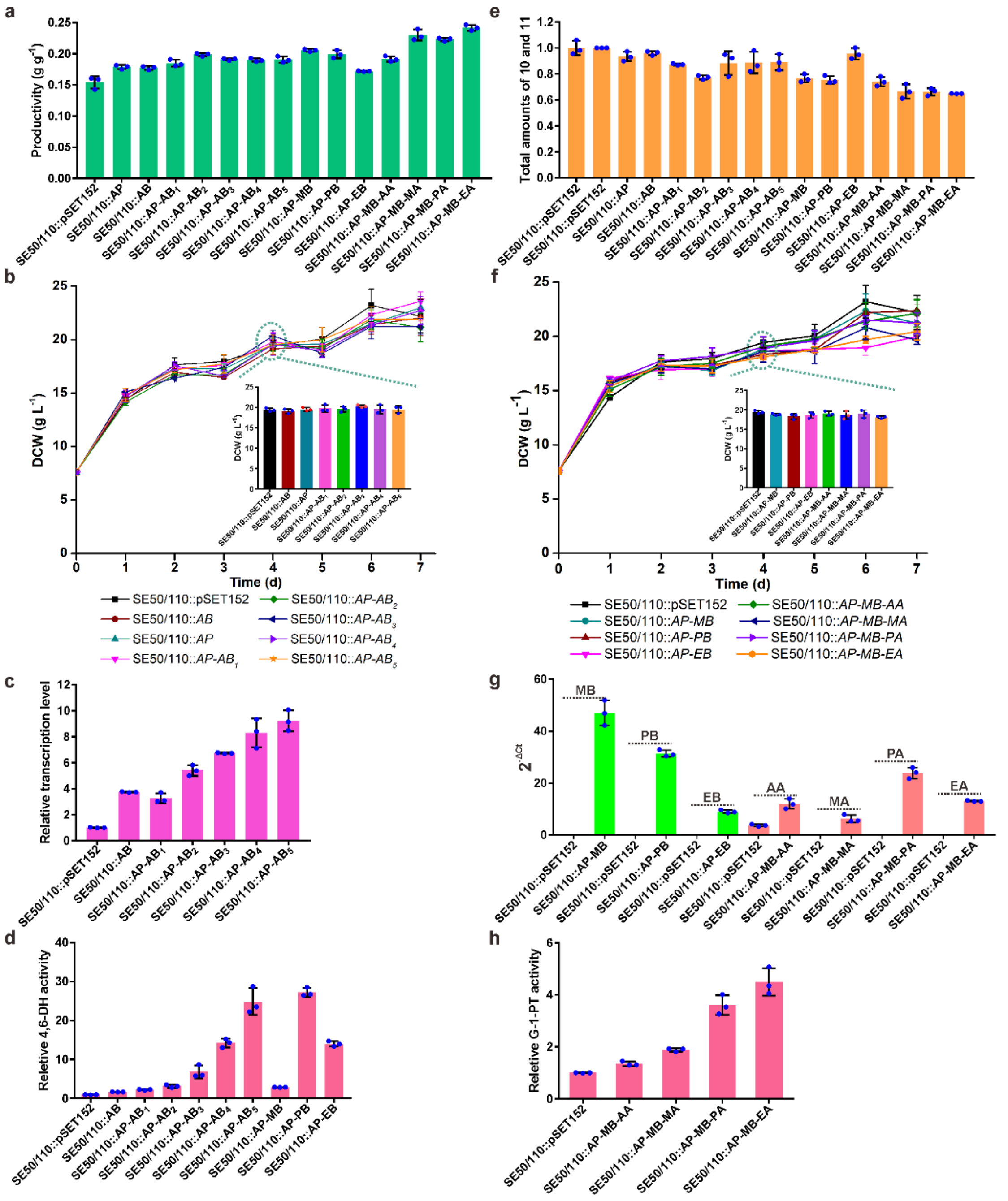

Supplementary Figure 46. Increasing the supply of amino-deoxyhexose moiety. a Calculation of the productivity (ratio of titer of **1** and DCW) of the involved strains after 4-day fermentation. **b** The time courses of growth (including the biomass of day 4) of the involved strains. **c** Analysis of the relative transcription of *acbB* of the mutant carrying the cassettes *AP-AB$_n$* after 2-day fermentation. **d** Analysis of the relative 4,6-DH activity of the mutants carrying the cassettes *AB*, *AP-AB$_n$*, *AP-MB*, *AP-PB* or *AP-EB* after 2-day fermentation. **e** Analysis of the total amounts of endogenous **10** and **11** of the

involved strains after 2-day fermentation. **f** The time courses of growth (including the biomass of day 4) of the involved strains. **g** Analysis of the transcription of genes *MB*, *EB*, *PB*, *AA*, *MA*, *EA* and *PA* in the corresponding mutants after 2-day fermentation. The threshold cycle (C_t) of targeted genes and internal control (*hrdB*) were obtained from RT-qPCR for respective cDNA samples, and ΔC_t ($C_{t,target}$ - $C_{t,hrdB}$) was then calculated. The results ($2^{-\Delta\Delta Ct}$) show the relative transcription of corresponding genes compared with *hrdB* in the mutants, indicating obvious transcription of heterogeneous genes in *Actinoplanes* sp. SE50/110. **h** Analysis of the relative G-1-PT activity of the mutants carrying the cassettes *AP-MB-AA*, *AP-MB-MA*, *AP-MB-PA* or *AP-MB-EA* after 2-day fermentation. DCW is the abbreviation of dry cell weight. Error bars, mean ± SD (n=3 biological replicates).

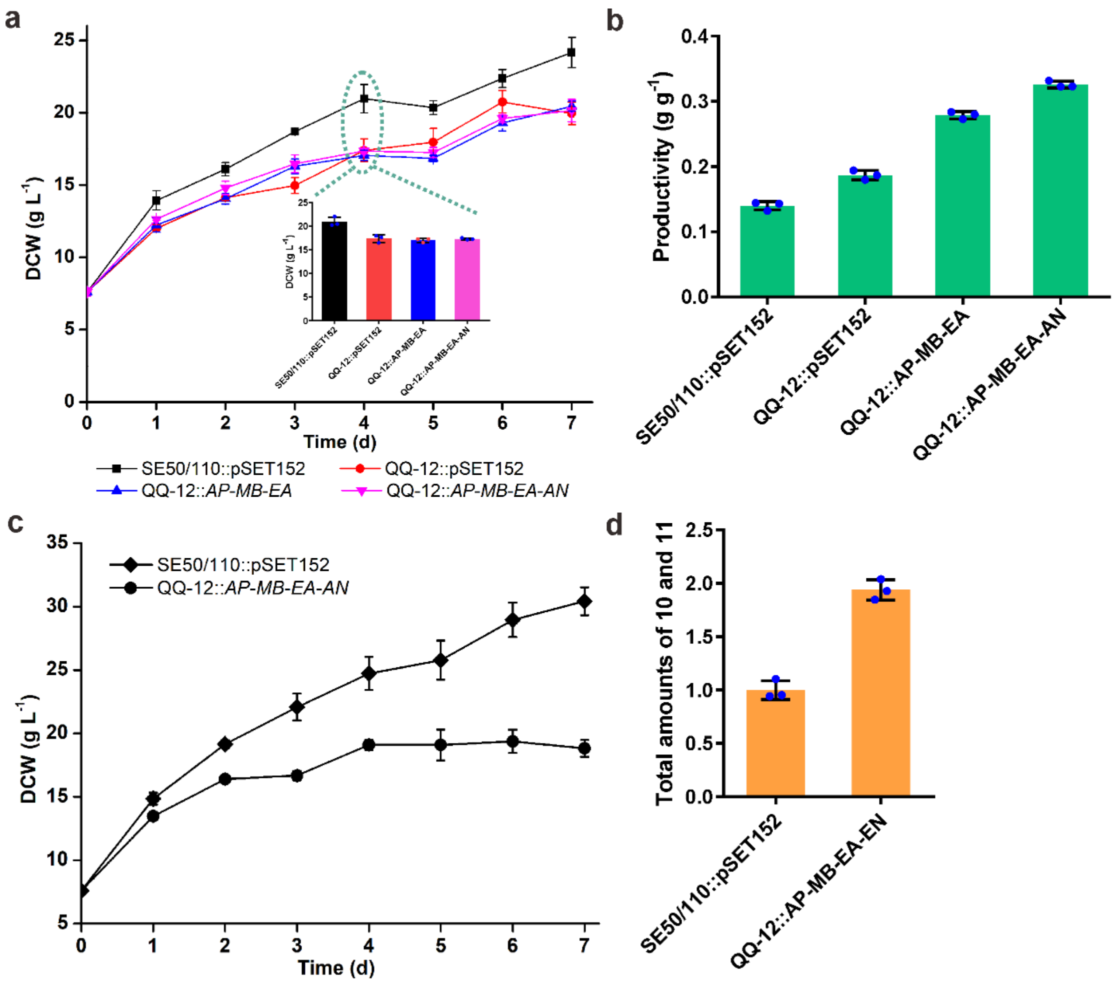

Supplementary Figure 47. Integration of effective engineering strategies. **a** The time courses of growth (including the biomass of day 4) of SE50/110::pSET152 (as control), QQ-12::pSET152 (as control), QQ-12::*AP-MB-EA* and QQ-12::*AP-MB-EA-AN*. **b** Calculation of the productivity (ratio of titer of **1** and DCW) of the involved strains after 4-day fermentation. **c** Time courses of the growth of SE50/110::pSET152 and QQ-12::*AP-MB-EA-EN* during fed-batch fermentation. **d** Analysis of the total amounts of endogenous **10** and **11** of SE50/110::pSET152 and QQ-12::*AP-MB-EA-EN* after 2-day fermentation. DCW is the abbreviation of dry cell weight. Error bars, mean ± SD (n=3 biological replicates).

a

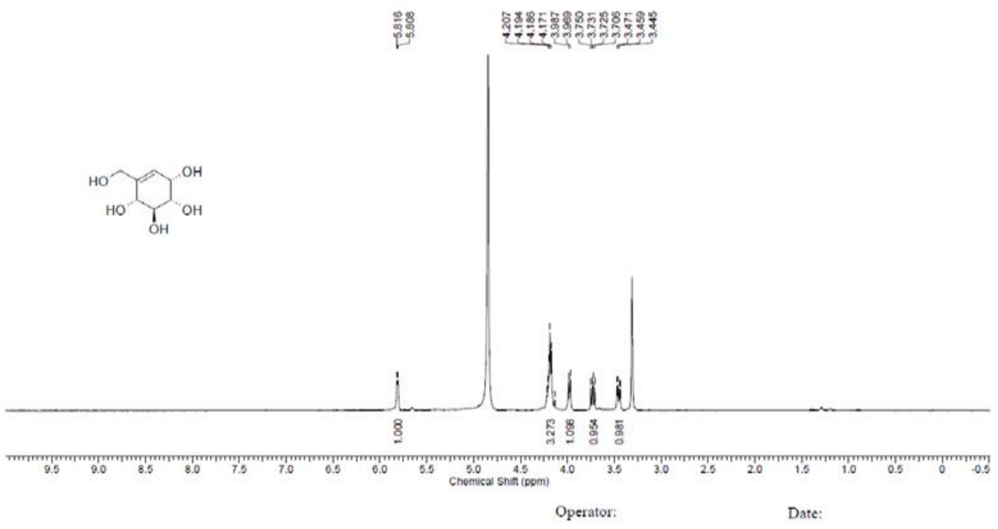

b

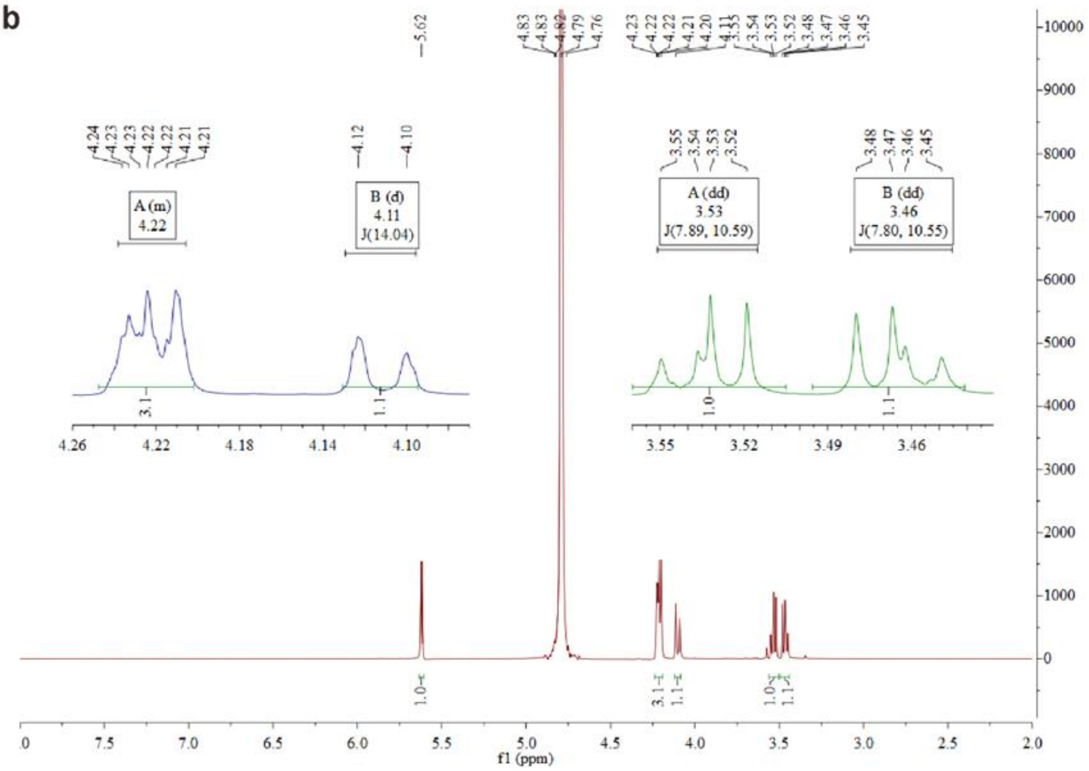

Supplementary Figure 48. ^{1}H NMR spectra of the standards 9 and 8. a ^{1}H NMR spectrum of **9** was provided by WuXi Apptec Co., Ltd. (400 MHz). **b** ^{1}H NMR spectrum of **8** was collected at 600 MHz Nuclear Magnetic Resonance (Avance III600 MHz, Bruker). To confirm the structures of the shunt products, we initially purified **8** and **9** from the fermentation broth of *Actinoplanes* sp. SE50/110. However, to avoid any effects of impurity of the compounds on

following feeding experiment and *in vitro* assay, **9** were chemically synthesized by WuXi Apptec Co., Ltd., and its structure was confirmed by NMR. Meanwhile, **8** was isolated from the fermentation broth of SE50/110Δ*acbN*, which had been proved to only produce **8** (Supplementary Fig. 34), and its structure was also confirmed by NMR. As shown in the ^{1}H NMR spectra, the purity of these compounds is good.

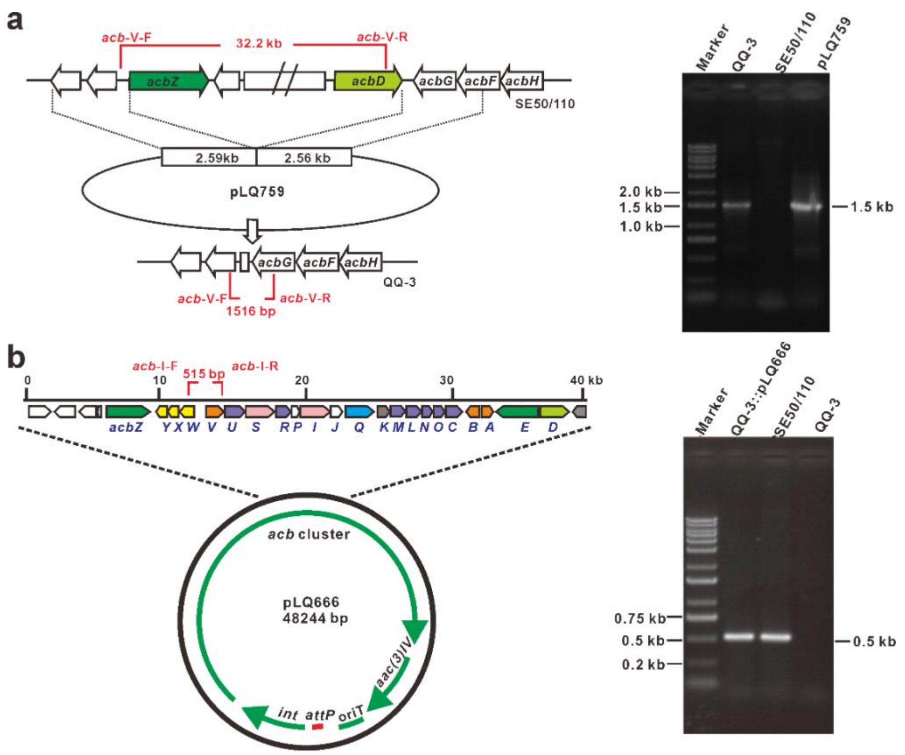

Supplementary Figure 49. Construction and verification of mutants QQ-3 and QQ-3::pLQ666. a Deletion of *acb* cluster (spanning a 32.2-kb region of DNA from *acbZ* to upstream region of *acbD*) in *Actinoplanes* sp. SE50/110. Schematic representation of the deletion of *acb* cluster and confirmation of the mutant QQ-3 by PCR amplification. Using primers *acb*-V-F and *acb*-V-R, a 1.5-kb fragment was amplified using the genomic DNA of QQ-3 or the recombinant plasmid pLQ759 (positive control) as templates, whereas the genomic DNA of *Actinoplanes* sp. SE50/110 (negative control) gave no amplified product. **b** Trans-complementation of *acb* cluster by introducing pLQ666 into mutant QQ-3. The pLQ666 with whole *acb* cluster and cassette of *int-attP-oriT-aac(3)IV* and confirmation of the complemented mutant QQ-3::pLQ666 by PCR amplification. Using primers *acb*-I-F and *acb*-I-R, a 0.5-kb fragment was amplified using the genomic DNA of QQ-3::pLQ666 or *Actinoplanes* sp. SE50/110 (positive control) as templates, whereas the genomic DNA of QQ-3 (negative control) gave no amplified product. Similar results of confirmation of each gene-deleted mutant were obtained in three independent experiments.

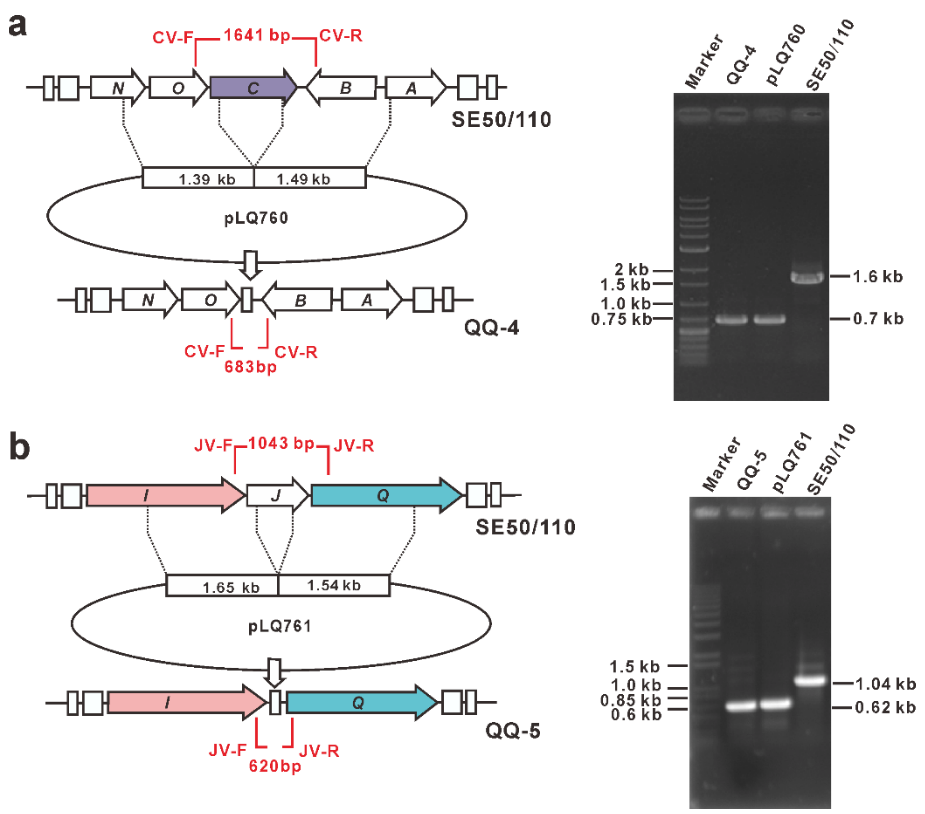

Supplementary Figure 50. Construction and verification of mutants QQ-4 and QQ-5. a Deletion of *acbC* in *Actinoplanes* sp. SE50/110. Schematic representation of the deletion of *acbC* and confirmation of the mutant QQ-4 by PCR amplification. Using primers CV-F and CV-R, a 0.7-kb fragment was amplified using the genomic DNA of QQ-4 or the recombinant plasmid pLQ760 (positive control) as templates, whereas the genomic DNA of *Actinoplanes* sp. SE50/110 (negative control) gave a 1.6-kb amplified product. **b** Deletion of *acbJ* in *Actinoplanes* sp. SE50/110. Schematic representation of the deletion of *acbJ* and confirmation of the mutant QQ-5 by PCR amplification. Using primers JV-F and JV-R, a 0.62-kb fragment was amplified using the genomic DNA of QQ-5 or the recombinant plasmid pLQ761 (positive control) as templates, whereas the genomic DNA of *Actinoplanes* sp. SE50/110 (negative control) gave a 1.04-kb amplified product. Similar results of confirmation of each gene-deleted mutant were obtained in three independent experiments.

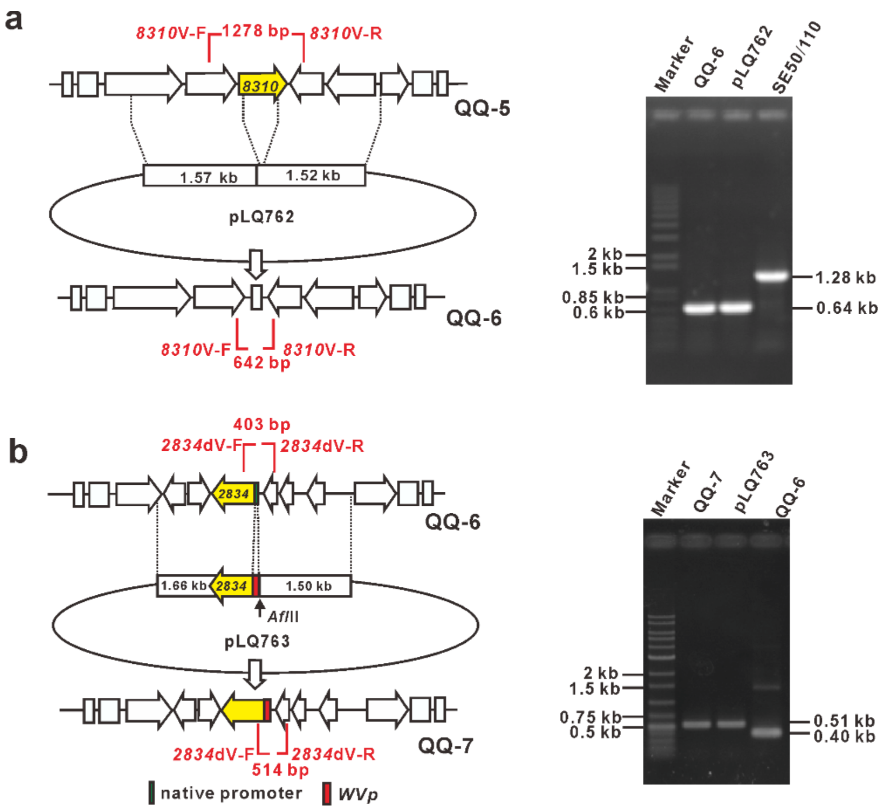

Supplementary Figure 51. Construction and verification of mutants QQ-6 and QQ-7. a Deletion of *ACPL_8310* in QQ-5. Schematic representation of the deletion of *ACPL_8310* and confirmation of the mutant QQ-6 by PCR amplification. Using primers *8310*V-F and *8310*V-R, a 0.64-kb fragment was amplified using the genomic DNA of QQ-6 or the recombinant plasmid pLQ762 (positive control) as templates, whereas the genomic DNA of QQ-5 (negative control) gave a 1.28-kb amplified product. **b** Down-regulation of the expression of *ACPL_2834* in QQ-6. Schematic representation of the replacement of the native promoter of *ACPL_2834* to *WVp* in QQ-6 and confirmation of the mutant QQ-7 by PCR amplification. Using primers *2834*d-V-F and *2834*d-V-R, a 0.51-kb fragment was amplified using the genomic DNA of QQ-7 or the recombinant plasmid pLQ763 (positive control) as templates, whereas the genomic DNA of QQ-6 (negative control) gave a 0.40-kb amplified product. Similar results of confirmation of each gene-deleted mutant were obtained in three independent experiments.

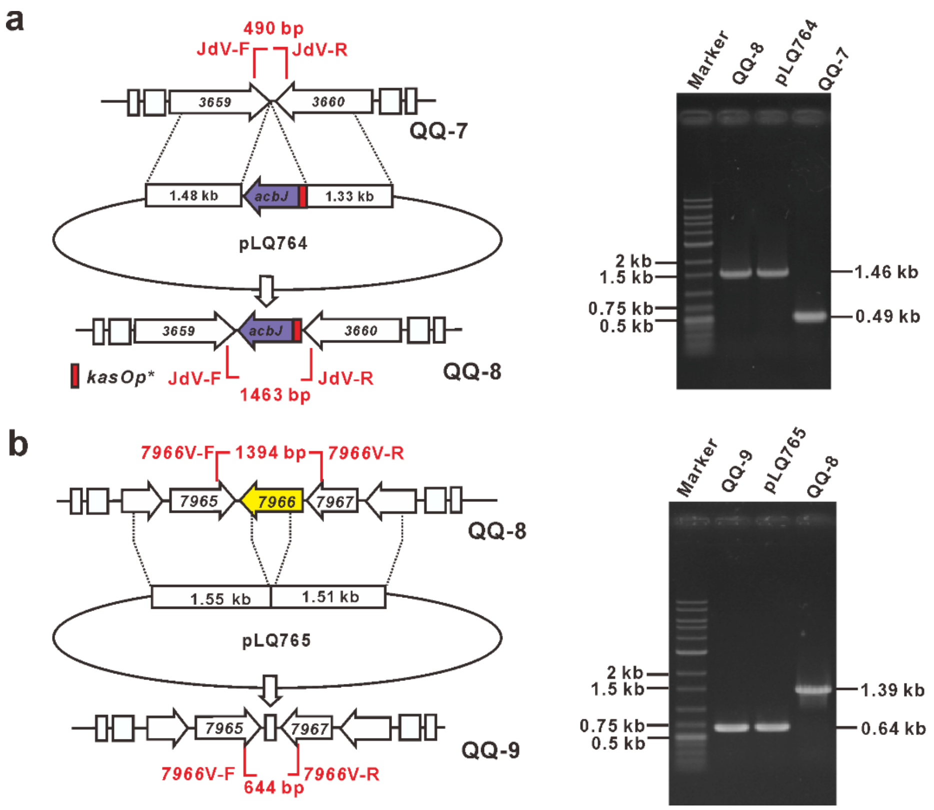

Supplementary Figure 52. Construction and verification of mutants QQ-8 and QQ-9. a Insertion of *kasOp*-acbJ* in the genome of QQ-7. Schematic representation of the introduction of *kasOp*-acbJ* in the genome of QQ-7 and confirmation of the mutant QQ-8 by PCR amplification. Using primers Jd-V-F and Jd-V-R, a 1.46-kb fragment was amplified using the genomic DNA of QQ-8 or the recombinant plasmid pLQ764 (positive control) as templates, whereas the genomic DNA of QQ-7 (negative control) gave a 0.49-kb amplified product. **b** Deletion of *ACPL_7966* in QQ-8. Schematic representation of the deletion of *ACPL_7966* and confirmation of the mutant QQ-9 by PCR amplification. Using primers *7966*V-F and *7966*V-R, a 0.64-kb fragment was amplified using the genomic DNA of QQ-9 or the recombinant plasmid pLQ765 (positive control) as templates, whereas the genomic DNA of QQ-8 (negative control) gave a 1.39-kb amplified product. Similar results of confirmation of each gene-deleted mutant were obtained in three independent experiments.

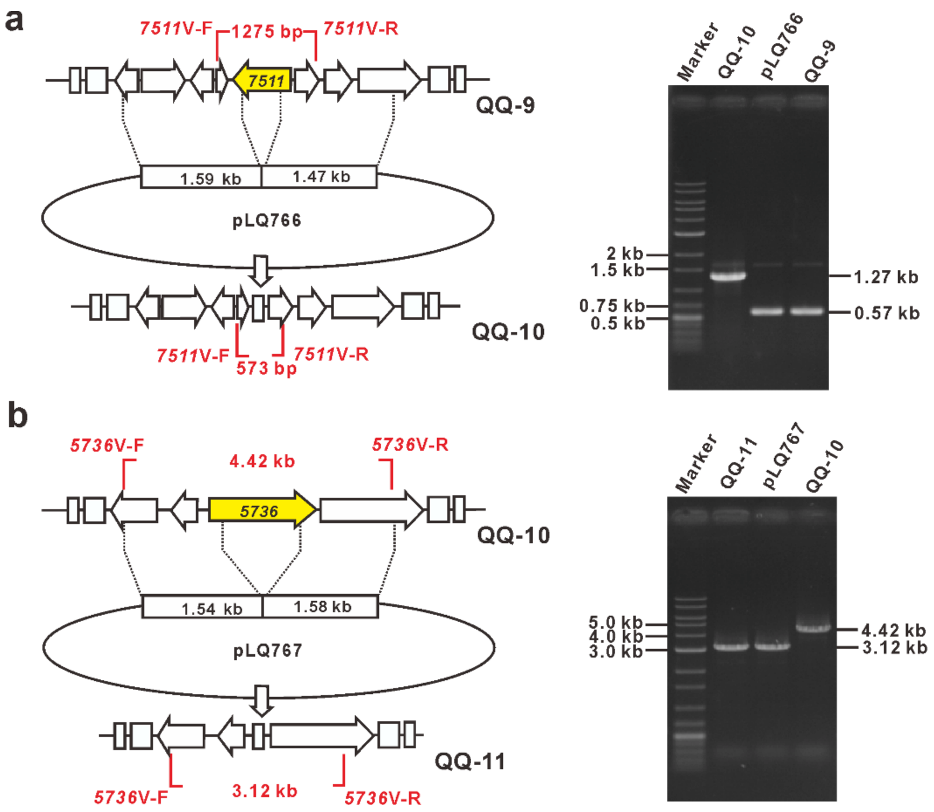

Supplementary Figure 53. Construction and verification of mutants QQ-10 and QQ-11. a Deletion of *ACPL_7511* in QQ-9. Schematic representation of the deletion of *ACPL_7511* and confirmation of the mutant QQ-10 by PCR amplification. Using primers *7511*V-F and *7511*V-R, a 0.57-kb fragment was amplified using the genomic DNA of QQ-10 or the recombinant plasmid pLQ768 (positive control) as templates, whereas the genomic DNA of QQ-9 (negative control) gave a 1.27-kb amplified product. **b** Deletion of *ACPL_5736* in QQ-10. Schematic representation of the deletion of *ACPL_5736* and confirmation of the mutant QQ-11 by PCR amplification. Using primers *5736*V-F and *5736*V-R, a 3.12-kb fragment was amplified using the genomic DNA of QQ-11 or the recombinant plasmid pLQ767 (positive control) as templates, whereas the genomic DNA of QQ-10 (negative control) gave a 4.42-kb amplified product. Similar results of confirmation of each gene-deleted mutant were obtained in three independent experiments.

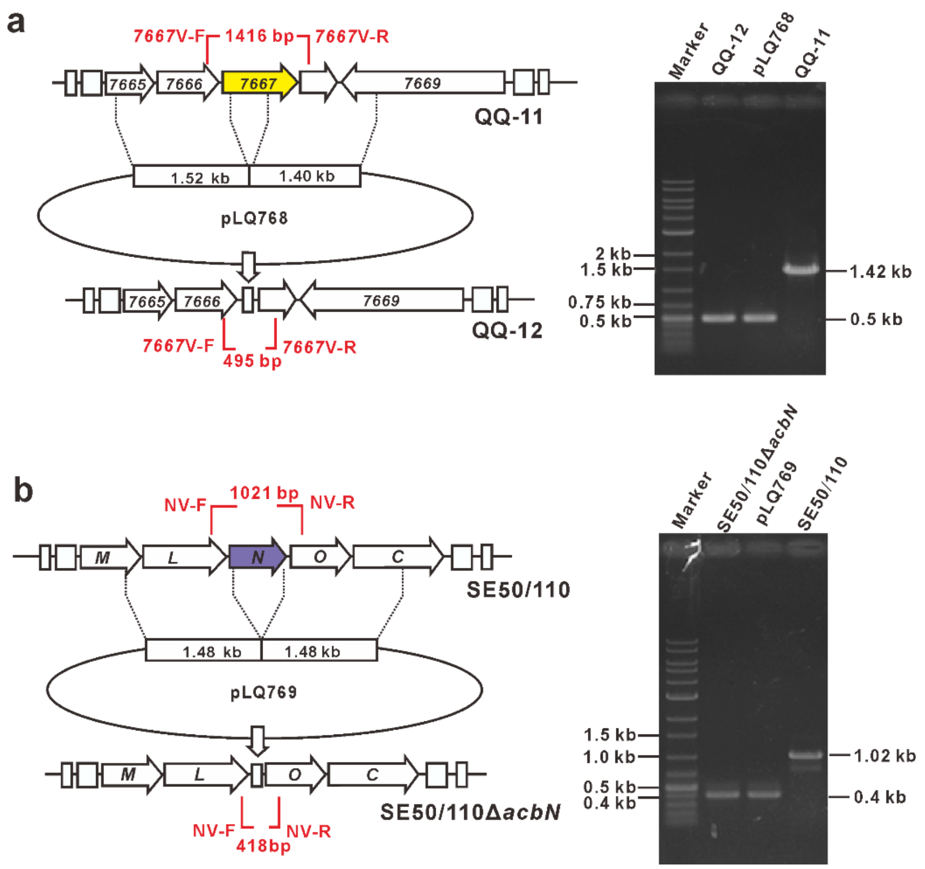

Supplementary Figure 54. Construction and verification of mutants QQ-12 and SE50/110Δ*acbN*. a Deletion of *ACPL_7667* in QQ-11. Schematic representation of the deletion of *ACPL_7667* and confirmation of the mutant QQ-12 by PCR amplification. Using primers *7667*V-F and *7667*V-R, a 0.5-kb fragment was amplified using the genomic DNA of QQ-12 or the recombinant plasmid pLQ768 (positive control) as templates, whereas the genomic DNA of QQ-11 (negative control) gave a 1.42-kb amplified product. **b** Deletion of *acbN* in *Actinoplanes* sp. SE50/110. Schematic representation of the deletion of *acbN* and confirmation of the mutant SE50/110Δ*acbN* by PCR amplification. Using primers NV-F and NV-R, a 0.4-kb fragment was amplified using the genomic DNA of SE50/110Δ*acbN* or the recombinant plasmid pLQ769 (positive control) as templates, whereas the genomic DNA of *Actinoplanes* sp. SE50/110 (negative control) gave a 1.02-kb amplified product. Similar results of confirmation of each gene-deleted mutant were obtained in three independent experiments.

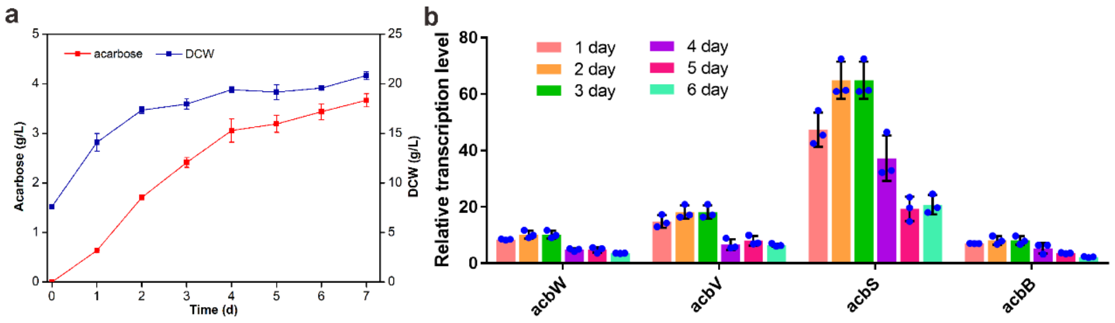

Supplementary Figure 55. Monitor of the parameters during the fermentation of *Actinoplanes* sp. SE50/110. a Time courses of growth and titer of **1** of *Actinoplanes* sp. SE50/110; **b** The transcription of *acbW*, *acbV*, *acbS* and *acbB*. Error bars, mean ± SD (n=3 biological replicates).

Supplementary Table 1. Proteins encoded by *acb* cluster of *Actinoplanes* sp. SE50/110 and their putative functions[2].

Gene_ID	Proteins	AA	(Putative) functions
ACPL_3680	AcbC	398	2-*epi*-5-*epi*-valiolone synthase
ACPL_3676	AcbM	359	2-*epi*-5-*epi*-valiolone 7-kinase
ACPL_3679	AcbO	270	2-*epi*-5-*epi*-valiolone-7-P 2-epimerase
ACPL_3677	AcbL	366	cyclitol dehydrogenase
ACPL_3678	AcbN	251	cyclitol oxidoreductase
ACPL_3668	AcbU	492	kinase (1-*epi*-valienol-7-P 1-kinase)
ACPL_3670	AcbR	363	1-*epi*-valienol-1,7-diP 1-adenylyltransferase
ACPL_3682	AcbA	278	glucose-1-P thymidylyltransferase (G-1-PT)
ACPL_3681	AcbB	320	dTDP-glucose 4,6-dehydratase (4,6-DH)
ACPL_3667	AcbV	431	dTDP-4-keto-6-deoxy-glucose 4-aminotransferase
ACPL_3669	AcbS	709	glycosyltransferase
ACPL_3672	AcbI	734	glycosyltransferase
ACPL_3674	AcbQ	698	acarbose 4-α-glucanotransferase
ACPL_3673	AcbJ	283	Haloacid dehalogenase-like (HAD) superfamily hydrolase
ACPL_3671	AcbP	195	NUDIX hydrolase
ACPL_3675	AcbK	299	acarbose 7-kinase
ACPL_3684	AcbD	724	acarviose transferase
ACPL_3683	AcbE	1033	acarbose resistant α-amylase
ACPL_3663	AcbZ	1075	α-amylase
ACPL_3666	AcbW	357	ABC transporter ATP-binding protein
ACPL_3665	AcbX	267	ABC transporter permease protein
ACPL_3664	AcbY	267	ABC transporter permease protein

AA: The length of protein sequences. The functions of AcbC, AcbM, AcbO, AcbK, AcbA, AcbB and AcbV have been demonstrated by *in vitro* enzymatic assays. The functions of other proteins are putative.

Supplementary Table 2. ^{1}H and ^{13}C NMR data for 1-*epi*-valienol and valienol in D_2O.

Position	1-*epi*-valienol (**8**)		valienol (**9**)	
	δ_H (δ in ppm, J in Hz)	δ_C (δ in ppm)	δ_H (δ in ppm, J in Hz)	δ_C (δ in ppm)
1	4.22, overlapped	71.3, CH	4.29, t (4.8)	68.9, CH
2	3.46, dd (10.6, 7.8)	75.1, CH	3.59, dd (10.7, 4.2)	73.5, CH
3	3.53, dd (10.6, 7.9)	75.5, CH	3.71, dd (10.7, 7.9)	75.3, CH
4	4.22, overlapped	71.8, CH	4.09, d (7.9)	75.0, CH
5	-	138.3, C	-	145.0, C
6	5.62, s	124.9, CH	5.86, dd (5.3, 1.2)	124.9, CH
7	a, overlapped<br>b, 4.10, d (14.0)	60.9, CH_2	a, 4.24, d (14.3)<br>b, 4.15, d (14.2)	64.1, CH_2

^{1}H and ^{13}C NMR data were recorded at 600 and 150 MHz, respectively.

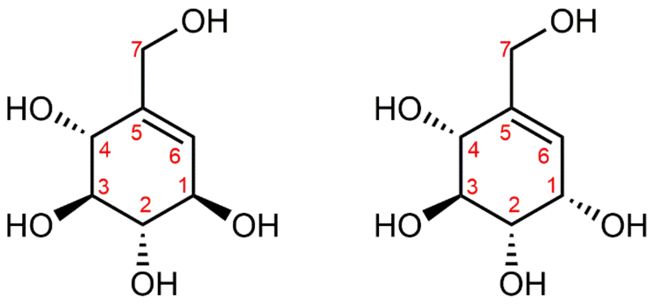

1-*epi*-valienol (**8**)   valienol (**9**)

Supplementary Table 3. ^{1}H and ^{13}C NMR data for 1-*epi*-valienol-7-P and valienol-7-P in D_2O.

Position	1-*epi*-valienol-7-P (**10**)		valienol-7-P (**11**)	
	δ_H (δ in ppm, J in Hz)	δ_C (δ in ppm, J in Hz)	δ_H (δ in ppm, J in Hz)	δ_C (δ in ppm, J in Hz)
1	4.18, overlapped	74.2, CH	4.29, t (4.8)	69.9, CH
2	3.50, dd (10.5, 7.9)	78.0, CH	3.62, dd (10.7, 4.2)	74.4, CH
3	3.54, dd (10.5, 7.8)	78.2, CH	3.72, dd (12.0, 6.5)	76.1, CH
4	4.30, d (7.6)	74.4, CH	4.13, d (7.8)	75.6, CH
5	-	139.3, C, **d (6.5)**	-	143.6, C, **d (6.5)**
6	5.67, s	129.1, CH	5.95, d (5.4)	127.4, CH
7	a, 4.46,m<br>b, 4.18, overlapped	67.2, CH_2, **d (4.5)**	a, 4.53, **dd (13.2, 8.5)**<br>b, 4.38, **dd (13.3, 6.8)**	68.5, CH_2, **d (4.8)**

^{1}H and ^{13}C NMR data were recorded at 600 and 150 MHz, respectively.

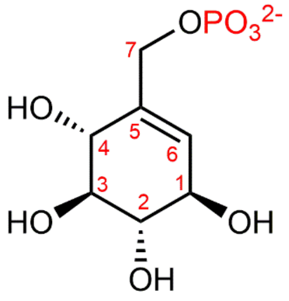

1-*epi*-valienol-7-P (**10**)

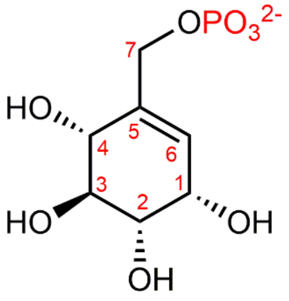

valienol-7-P (**11**)

Supplementary Table 4. Phosphatase candidates probably involved in the dephosphorylation of 10 and 11.

Gene ID	Gene	Putative function	Identity with AcbJ[a]	Transcription[b] (FPKM)	Categories
ACPL_3673	*acbJ*	HAD family hydrolase	100%	3333.3	Hydrolases showing higher similarity with AcbJ
ACPL_8310		HAD family phosphatase	31.93%	66.0	
ACPL_8309		HMP-PP phosphatase	34.04%	43.1	
ACPL_6858		unknown	30.21%	12.5	
ACPL_7709	*otsB*	HAD family hydrolase (trehalose-6-P phosphatase)	15.79%	591.0	
ACPL_2834		HAD-superfamily hydrolase (phosphoglucomutase)	16.96%	216.7	Genes having higher transcription in the HAD hydrolase family
ACPL_1897		HAD-hydrolase	16.90%	91.8	
ACPL_560		HAD-hydrolase	14.84%	72.0	
ACPL_7310		HAD-hydrolase (*N*-acylneuraminate-9-phosphatase)	14.84%	32.7	

[a] Alignment of amino acid sequences of the phosphatase candidates and AcbJ, respectively, by ClustalX 2.0.11.

[b] The transcription of the candidate genes analyzed by RNA-seq.

Supplementary Table 5. ^{1}H and ^{13}C NMR blended data for valienol-7-Pand valienone-7-P in D_2O.

Position	valienol-7-P (**11**)		valienone-7-P (**12**)	
	δ_H (δ in ppm, *J* in Hz)	δ_C (δ in ppm, *J* in Hz)	δ_H (δ in ppm, *J* in Hz)	δ_C (δ in ppm, *J* in Hz)
1	4.26, t (4.8)	68.9, CH	-	202.6, C
2	3.58, dd (10.7,4.3)	73.5, CH	4.23, d (11.0)	79.0, CH
3	3.68, overlapped	75.1, CH	3.75, dd (11.0, 8.6)	79.8, CH
4	4.11, d (7.9)	74.6, CH	4.53, d (8.8)	74.6, CH
5	-	143.0, C, d (5.8)	-	166.3, C, d (6.6)
6	5.90, d (5.4)	126.3, CH	6.24, s	124.4, CH
7	4.49, dd (13.2, 8.3)<br>4.31, dd (13.3, 6.6)	67.4, CH_2, d (4.3)	4.67, dd (17.7, 7.2)<br>4.61, dd (17.3, 7.2)	66.3, CH_2, d (3.8)

^{1}H and ^{13}C NMR data were recorded at 700 and 175 MHz, respectively.

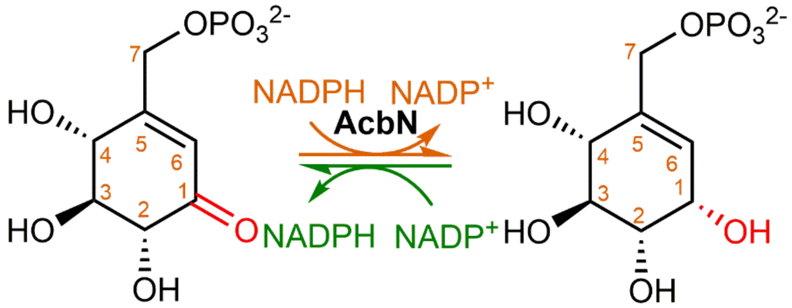

12 **11**

Supplementary Table 6. ^{1}H and ^{13}C NMR data for 1-*epi*-valienol-7-P in D_2O.

Position	δ_H (δ in ppm, J in Hz)	δ_C NMR (δ in ppm, J in Hz)
1	4.15, d (7.8)	74.3, CH
2	3.49, dd (8.4, 10.7)	78.0, CH
3	3.45, dd (7.9, 10.5)	78.0, CH
4	4.22, m	74.3, CH
5	-	**139.4, C, d (4.06)**
6	5.71, s	129.0, CH
7	4.43, m<br>4.25, overlapped	67.2, CH_2

^{1}H and ^{13}C NMR data were recorded at 700 and 175 MHz, respectively.

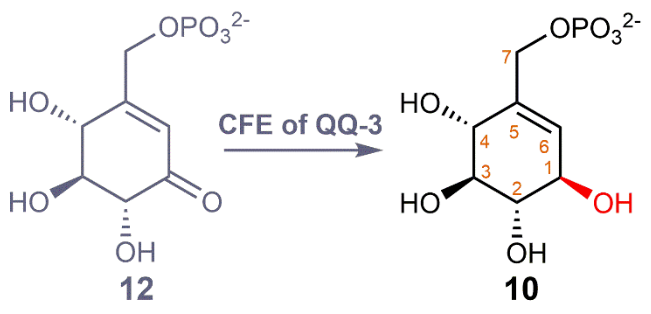

CFE: Cell-free extract

Supplementary Table 7. Oxidoreductase candidates probably involved in the conversion of 12 to 10.

Categories	Gene ID	Putative function	Unique PepCount	Transcription (FPKM)	Supplied in reaction mixture	Reaction[a]	Specific activity (U/mg)
Selected from group (e)	ACPL_7366	3-isopropylmalate dehydrogenase	12	226.72	NADPH	N	
	ACPL_7966	gfo/Idh/MocA family oxidoreductase	11	14.41	NADPH	Y	0.17 ± 0.013
	ACPL_7511	inositol 2-dehydrogenase	11	22.64	NADPH	Y	0.20 ± 0.03
	ACPL_706	3-oxoacyl-[acyl-carrier protein] reductase	10	36.75	NADPH	N	
	ACPL_6412	*N*-acetyl-γ-glutamyl-phosphate reductase	10	25.30	NADPH	N	
	ACPL_2464	betaine-aldehyde dehydrogenase	6	14.44	NADPH	N	
	ACPL_1863	glucose-6-phosphate 1-dehydrogenase	4	280.44	NADPH	Y	0.02 ± 0.00
	ACPL_5736	glucose-6-phosphate 1-dehydrogenase	4	12.45	NADPH	Y	1.49 ± 0.06
Selected from Group (g)	ACPL_2126	acyl-CoA dehydrogenase fadE4	23	27.83	BCF	N	
	ACPL_7667	acyl-CoA dehydrogenase	22	49.49	BCF	Y	0.053 ± 0.002
	ACPL_7021	acyl-CoA dehydrogenase	17	14.85	BCF	N	
	ACPL_2420	acyl-CoA dehydrogenase	16	66.34	BCF	N	
	ACPL_1809	pyridoxamine 5'-phosphate oxidase	8	66.75	BCF	N	
	ACPL_5569	acyl-CoA oxidase	8	17.00	BCF	N	
	ACPL_183	acyl-CoA dehydrogenase	8	118.80	BCF	N	
	ACPL_4412	glycine dehydrogenase	7	106.36	BCF	Y	0.020 ± 0.002
	ACPL_5740	acyl-CoA dehydrogenase	5	21.09	BCF	Y	0.059 ± 0.004
	ACPL_8197	molybdopterin oxidoreductase	5	139.62	BCF	N	

[a] reduction of **12** to **10**. “Y”, has activity; “N”, without activity.

Supplementary Table 8. Strains used in this study.

Strains	Features and functions	Sources or references
Actinoplanes **sp.**		
SE50/110	High yield mutant derived from SE50	American Type Culture Collection
QQ-3	Deletion of whole *acb* cluster (32.2 kb) in SE50/110	This work
QQ-3::pLQ666	Complementation of *acb* cluster with pLQ666 in QQ-3	This work
QQ-4	In-frame deletion of *acbC* in SE50/110	This work
QQ-5	In-frame deletion of *acbJ* in SE50/110	This work
SE50/110::pSET152	Integration of pSET152 in SE50/110	This work
QQ-5::pSET152	Integration of pSET152 in QQ-5	This work
QQ-5:: *acbJ*	Complementation of *acbJ* with pSET152-*kasOp*-acbJ*	This work
QQ-6	Deletion of *ACPL_8310* in QQ-5	This work
QQ-7	Down-regulation of the expression of *ACPL_2834* in QQ-6	This work
QQ-4::*valC*	Expression of *valC* in QQ-4	This work
QQ-8	Introduction of *kasOp*-acbJ* in the chromosome of QQ-7	This work
QQ-9	Deletion of *ACPL_7966* in QQ-8	This work
QQ-10	Deletion of *ACPL_7511* in QQ-9	This work
QQ-11	Deletion of *ACPL_5736* in QQ-10	This work
QQ-12	Deletion of *ACPL_7667* in QQ-11	This work
SE50/110Δ*acbN*	In-frame deletion of *acbN* in SE50/110	This work
QQ-12::pSET152	Integration of pSET152 in QQ-12	This work
QQ-12::*AN*	Expression of *acbN* under the control of *ermEp** in QQ-12	This work
QQ-5:: *ermEp*-acbJ*	Expression of *acbJ* under the control of *ermEp** in QQ-5	This work
QQ-5::*groLp -acbJ*	Expression of *acbJ* under the control of *groLp* in QQ-5	This work
QQ-5::*gapAp-acbJ*	Expression of *acbJ* under the control of *gapAp* in QQ-5	This work
QQ-5::*WVp-acbJ*	Expression of *acbJ* under the control of *WVp* in QQ-5	This work
SE50/110::*AP*	Expression of *pgmA* in SE50/110	This work
SE50/110::*AB*	Expression of *acbB* in SE50/110	This work
SE50/110::$AP\text{-}AB_1$	Co-expression of *pgmA* and *acbB* in SE50/110	This work
SE50/110:: $AP\text{-}AB_2$	Co-expression of *pgmA* and two copies of *acbB* in SE50/110	This work
SE50/110:: $AP\text{-}AB_3$	Co-expression of *pgmA* and three copies of *acbB* in SE50/110	This work
SE50/110:: $AP\text{-}AB_4$	Co-expression of *pgmA* and four copies of *acbB* in SE50/110	This work
SE50/110:: $AP\text{-}AB_5$	Co-expression of *pgmA* and five copies of *acbB* in SE50/110	This work

SE50/110::*AP-MB*	Co-expression of *pgmA* and *rmlB* from *M. smegmatis* in SE50/110	This work
SE50/110::*AP-PB*	Co-expression of *pgmA* and *rmlB* from *P. aeruginosa* in SE50/110	This work
SE50/110::*AP-EB*	Co-expression of *pgmA* and *rfbB* from *E. coli* in SE50/110	This work
SE50/110::*AP-MB-AA*	Co-expression of *AP-MB* and *acbA* from *Actinoplanes* sp. in SE50/110	This work
SE50/110::*AP-MB-MA*	Co-expression of *AP-MB* and *rmlA* from *M. smegmatis* in SE50/110	This work
SE50/110::*AP-MB-PA*	Co-expression of *AP-MB* and *rmlA* from *P. aeruginosa* in SE50/110	This work
SE50/110::*AP-MB-EA*	Co-expression of *AP-MB* and *rfbA* from *E. coli* in SE50/110	This work
QQ-12::*AP-MB*	Expression of *AP-MB* in QQ-12	This work
QQ-12::*AP-MB-EA*	Expression of *AP-MB-EA* in QQ-12	This work
QQ-12::*AP-MB-EA-AN*	Co-expression of *AP-MB-EA* and *acbN* in QQ-12	This work
E. coli		
DH10B	F^- *mcrA* Δ(mrr-*hsd*RMS-*mcr*BC) φ80*lacZ*ΔM15 Δ*lac*X74 *rec*A1 *end*A1 *ara*D139 Δ (*ara*, *leu*)7997 *gal*E15 *gal*K λ - *rsp*L *nup*G	Invitrogen
ET12567(pUZ8002)	F^- dam-13::Tn9 dcm-6 *hsd*M *hsd*R zjj-202::Tn10 *rec*F143 *gal*K2 *gal*T22 *ara*-14 *lac*Y1 xyl-5 *leu*B6 thi-1 *ton*A31 *rps*L136 hisG4 tsx-78 mtl-1 *gln*V44 Cml^R Kan^R	[5]
BL21 (DE3)	F^- ompT hsdS(r_B^- m_B^-) gal dcm(DE3)	Sangon Biotech

Supplementary Table 9. Plasmids used in this study.

Plasmids	Features and functions	Sources or references
pLQ752	*rep* pIJ101, *aac(3)IV*, *oriT, codA*	[6]
PLQ759	pLQ752-derived plasmid for *acb* cluster deletion	This work
PLQ760	pLQ752-derived plasmid for *acbC* in-frame deletion	This work
PLQ761	pLQ752-derived plasmid for *acbJ* in-frame deletion	This work
PLQ762	pLQ752-derived plasmid for *ACPL_8310* in-frame deletion	This work
PLQ763	pLQ752-derived plasmid for downregulation of *ACPL_2834* under the control of *WVp*	This work
PLQ764	pLQ752-derived plasmid for down-regulation of *acbJ* under the control of *kasOp**	This work
PLQ765	pLQ752-derived plasmid for *ACPL_7966* deletion	This work
PLQ766	pLQ752-derived plasmid for ACPL_7511 deletion	This work
PLQ767	pLQ752-derived plasmid for ACPL_5736 deletion	This work
PLQ768	pLQ752-derived plasmid for ACPL_7667 deletion	This work
PLQ769	pLQ752-derived plasmid for *acbN* deletion	This work
pSET152	Ø31 *int*, *attP*, *oriT*, *aac(3)IV*	[5]
pSET152-M	Inserting the MCS (*Xba*I, *Bam*HI, *Afl*II, *Bgl*II, *Kpn*I, *Nde*I, *Sca*I, *Mfe*I, *Eco*RV, *Not*I) in pSET152	This work
PLQ666	Fosmid with a whole *acb* cluster and component of *int-attP-oriT-aac(3)IV* from pSET152	[6]
pSET152-*kasOp*-valC*	pSET152-derived plasmid for expression of *valC* under the control of *kasOp**	This work
pSET152-*ermEp*-acbN*	pSET152-derived plasmid for expression of *acbN* under the control of *ermEp**	This work
pSET152-*ermEp*-acbJ*	pSET152-derived plasmid for expression of *acbJ* under the control of *ermEp**	This work
pSET152-*kasOp*-acbJ*	pSET152-derived plasmid for expression of *acbJ* under the control of *kasOp**	This work
pSET152-*groLp-acbJ*	pSET152-derived plasmid for expression of *acbJ* under the control of *groLp*	This work
pSET152-*gapAp-acbJ*	pSET152-derived plasmid for expression of *acbJ* under the control of *gapAp*	This work
pSET152-*WVp-acbJ*	pSET152-derived plasmid for expression of *acbJ* under the control of *WVp*	This work
pSET152-*AP*	pSET152-derived plasmid for expression of *pgmA* under the control of *kasOp**	This work
pSET152-*AB*	pSET152-derived plasmid for expression of *acbB* under the control of *kasOp**	This work
pSET152-*AP-AB*$_1$	pSET152-derived plasmid for co-expression of *pgmA* and *acbB*	This work
pSET152-*AP-AB*$_2$	pSET152-derived plasmid for co-expression of *pgmA* and two copies *of acbB*	This work
pSET152-*AP-AB*$_3$	pSET152-derived plasmid for co-expression of *pgmA* and three copies *of acbB*	This work
pSET152-*AP-AB*$_4$	pSET152-derived plasmid for co-expression of *pgmA* and four copies *of acbB*	This work

pSET152-*AP-AB*$_5$	pSET152-derived plasmid for co-expression of *pgmA* and five copies *of acbB*	This work
pSET152-*AP-MB*	pSET152-derived plasmid for co-expression of *pgmA* and *rmlB* from *M. smegmatis*	This work
pSET152-*AP-PB*	pSET152-derived plasmid for co-expression of *pgmA* and *rmlB* from *P. aeruginosa*	This work
pSET152-*AP-EB*	pSET152-derived plasmid for co-expression of *pgmA* and *rfbB* from *E. coli*	This work
pSET152-*AP-MB-AA*	pSET152-derived plasmid for co-expression of *AP-MB* and *acbA* from SE50/110	This work
pSET152-*AP-MB-MA*	pSET152-derived plasmid for co-expression of *AP-MB* and *rmlA* from *M. smegmatis*	This work
pSET152-*AP-MB-PA*	pSET152-derived plasmid for co-expression of *AP-MB* and *rmlA* from *P. aeruginosa*	This work
pSET152-*AP-MB-EA*	pSET152-derived plasmid for co-expression of *AP-MB* and *rfbA* from *E. coli*	This work
pSET152-*AP-MB-EA-AN*	pSET152-derived plasmid for co-expression of *AP-MB-EA* and *acbN* from SE50/110	This work
PET-30a	Expression vector, pBR322 replicon, P_{T7} , His_6 Tag, Km^R	Takara
pET-30a-*valC*	pET30a-derived plasmid for expression of ValC in BL21(DE3)	This work
pET-30a-*acbJ*	pET30a-derived plasmid for expression of AcbJ in BL21(DE3)	This work
pET-30a-*ACPL_8310*	pET30a-derived plasmid for expression of ACPL_8310 in BL21(DE3)	This work
pET-30a-*ACPL_2834*	pET30a-derived plasmid for expression of ACPL_2834 in BL21(DE3)	This work
pET-30a-*ACPL_7709*	pET30a-derived plasmid for expression of ACPL_7709 in BL21(DE3)	This work
pET-30a-*ACPL_3673*	pET30a-derived plasmid for expression of ACPL_3673 in BL21(DE3)	This work
pET-30a-*ACPL_8309*	pET30a-derived plasmid for expression of ACPL_8309 in BL21(DE3)	This work
pET-30a-*ACPL_6858*	pET30a-derived plasmid for expression of ACPL_6858 in BL21(DE3)	This work
pET-30a-*ACPL_560*	pET30a-derived plasmid for expression of ACPL_560 in BL21(DE3)	This work
pET-30a-*ACPL_7310*	pET30a-derived plasmid for expression of ACPL_7310 in BL21(DE3)	This work
pET-30a-*ACPL_1897*	pET30a-derived plasmid for expression of ACPL_1897 in BL21(DE3)	This work
pET-30a-*ACPL_6648*	pET30a-derived plasmid for expression of ACPL_6648 in BL21(DE3)	This work
pET-30a-*acbM*	pET30a-derived plasmid for expression of AcbM in BL21(DE3)/pGro7	This work
pET-30a-*acbO*	pET30a-derived plasmid for expression of AcbO in BL21(DE3)/pGro7	This work
pET-30a-*acbL*	pET30a-derived plasmid for expression of AcbL in BL21(DE3)/pGro7	This work
pET-30a-*acbN*	pET30a-derived plasmid for expression of AcbN in BL21(DE3)/pGro7	This work
pET-30a-*ACPL_7366*	pET30a-derived plasmid for expression of ACPL_7366 in BL21(DE3)	This work
pET-30a-*ACPL_7966*	pET30a-derived plasmid for expression of ACPL_7966 in BL21(DE3)	This work
pET-30a-*ACPL_7511*	pET30a-derived plasmid for expression of ACPL_7511 in BL21(DE3)	This work
pET-30a-*ACPL_706*	pET30a-derived plasmid for expression of ACPL_706 in BL21(DE3)	This work

pET-30a-*ACPL_6412*	pET30a-derived plasmid for expression of ACPL_6412 in BL21(DE3)	This work
pET-30a-*ACPL_2464*	pET30a-derived plasmid for expression of ACPL_2464 in BL21(DE3)	This work
pET-30a-*ACPL_1863*	pET30a-derived plasmid for expression of ACPL_1863 in BL21(DE3)	This work
pET-30a-*ACPL_5736*	pET30a-derived plasmid for expression of ACPL_5736 in BL21(DE3)	This work
pET-30a-*ACPL_2126*	pET30a-derived plasmid for expression of ACPL_2126 in BL21(DE3)	This work
pET-30a-*ACPL_7667*	pET30a-derived plasmid for expression of ACPL_7667 in BL21(DE3)	This work
pET-30a-*ACPL_7021*	pET30a-derived plasmid for expression of ACPL_7021 in BL21(DE3)	This work
pET-30a-*ACPL_2420*	pET30a-derived plasmid for expression of ACPL_2420 in BL21(DE3)	This work
pET-30a-*ACPL_1809*	pET30a-derived plasmid for expression of ACPL_1809 in BL21(DE3)	This work
pET-30a-*ACPL_5569*	pET30a-derived plasmid for expression of ACPL_5569 in BL21(DE3)	This work
pET-30a-*ACPL_183*	pET30a-derived plasmid for expression of ACPL_183 in BL21(DE3)	This work
pET-30a-*ACPL_4412*	pET30a-derived plasmid for expression of ACPL_4412 in BL21(DE3)	This work
pET-30a-*ACPL_5740*	pET30a-derived plasmid for expression of ACPL_5740 in BL21(DE3)	This work
pET-30a-*ACPL_8197*	pET30a-derived plasmid for expression of ACPL_8197 in BL21(DE3)	This work
pET-30a-*ACPL_149*	pET30a-derived plasmid for expression of ACPL_149 in BL21(DE3)	This work
pET-30a-*ACPL_6241*	pET30a-derived plasmid for expression of ACPL_6241 in BL21(DE3)	This work
pET-30a-*ACPL_2491*	pET30a-derived plasmid for expression of ACPL_2491 in BL21(DE3)	This work
pET-30a-*ACPL_2175*	pET30a-derived plasmid for expression of ACPL_2175 in BL21(DE3)	This work

Supplementary Table 10. Compounds involved in this study and their analytic methods.

Compounds	ID	HPLC-TOF/MS			HPLC-QQQ/MS					GC-QMS		
		Negative ion model			Polarity	Precursor ion	Product ion	Fragmentor	Collision energy	Retention time (min)	Mass spectra	Unique ion
		$[M-H]^-$	$[M+Cl]^-$	$[M+H_3PO_4]^-$								
acarbose	**1**	644.2407	680.2174	742.2176	positive	646.3	304.1	118	25			
2-*epi*-5-*epi*-valiolone	**3**	191.0561	227.0328	289.0330								
5-*epi*-valiolol-7-P	**4**	271.0224	306.9991									
dTDP-4-keto-6-deoxy-D-glucose	**6**	545.0579			negative	545.1	321.4	118	22			
1-*epi*-valienol	**8**	175.0612	211.0379	273.0381						24.6	73.1, 147.0, 191.1, 265.1, 332.2	332.2
valienol	**9**	175.0612	211.0379	273.0381						23.3	73.1, 147.0, 191.1, 265.1, 332.2	332.2
1-*epi*-valienol-7-P	**10**	255.0275	291.0042		negative	255.1	78.9	134	26			
valienol-7-P	**11**	255.0275	291.0042		negative	255.1	78.9	134	26			
valienone-7-P	**12**	253.0119	288.9886		negative	253.0	96.9	91	14			
valienone	**13**	173.0455	209.0222	271.0224						24.6	73.1, 147.0, 204.1, 258.1, 357.1	258.1
dTDP-glucose		563.0685			negative	563.1	320.8	134	25			

Supplementary References

1 Zhang, C. S. *et al*. Biosynthesis of the C_7-cyclitol moiety of acarbose in *Actinoplanes* species SE50/110. 7-*O*-phosphorylation of the initial cyclitol precursor leads to proposal of a new biosynthetic pathway. *J. Biol. Chem*. **277**, 22853-22862 (2002).

2 Wehmeier, U. F. & Piepersberg, W. Biotechnology and molecular biology of the α-glucosidase inhibitor acarbose. *Appl. Microbiol. Biotechnol.* **63**, 613-625 (2004).

3 Giuseppe, B., Paolo, D., Luigi, G. P. & Raffaele, R. Determination of relative configuration in organic compounds by NMR spectroscopy and computational methods. *Chem. Rev.* **107**, 3744-3779 (2007).

4 Altschul, S. F., *et al*. Gapped BLAST and PSI-BLAST: a new generation of protein database search programs. *Nucleic Acids Res*. **25**, 3389-3402 (1997).

5 Paget, M. S. B., Chamberlin, L., Atrih, A., Foster, S. J., & Buttner, M. J. Evidence that the extracytoplasmic function sigma factor sigma^{E} is required for normal cell wall structure in *Streptomyces coelicolor* A3(2). *J. Bacteriol.* **181**, 204-211 (1999).

6 Zhao, Q., Xie, H., Peng, Y., Wang, X. & Bai, L. Improving acarbose production and eliminating the by-product component C with an efficient genetic manipulation system of *Actinoplanes* sp. SE50/110. *Synth. Syst. Biotechnol.* **2**, 302-309 (2017).
