# Supplementary material for: A severe leakage of intermediates to shunt products in acarbose biosynthesis
Source: Nat Commun. 2020 Mar 19;11:1468. doi: 10.1038/s41467-020-15234-8 (PMC7081202; doi:10.1038/s41467-020-15234-8)
Supplement: Supplementary file 3 — Description of Additional Supplementary Files [file 41467_2020_15234_MOESM3_ESM.docx]

**Description of Additional Supplementary Files**

File Name: Supplementary Data 1
Description: Primers used in this study.

File Name: Supplementary Data 2

Description: The nucleotide sequence of promoter WVp.

File Name: Supplementary Data 3

Description: The nucleotide sequence of promoter gapAp.

File Name: Supplementary Data 4

Description: The nucleotide sequence of promoter groLp.
